# Solvent co-intercalation in layered cathode active materials for sodium-ion batteries

**DOI:** 10.1038/s41563-025-02287-7

**Published:** 2025-07-18

**Authors:** Yanan Sun, Gustav Åvall, Shu-Han Wu, Guillermo A. Ferrero, Annica Freytag, Pedro B. Groszewicz, Hui Wang, Katherine A. Mazzio, Matteo Bianchini, Volodymyr Baran, Sebastian Risse, Philipp Adelhelm

**Affiliations:** 1 Institut für Chemie, Humboldt-Universität zu Berlin, Berlin, Germany; 2 Joint Research Group Operando Battery Analysis (CE-GOBA), Helmholtz-Zentrum Berlin für Materialien und Energie (HZB), Berlin, Germany; 3 SEEL Swedish Electric Transport Laboratory, Gothenburg, Sweden; 4 SE-ASPIN, Helmholtz-Zentrum Berlin für Materialien und Energie (HZB), Berlin, Germany; 5 Department of Radiation Science and Technology, Delft University of Technology, Delft, Netherlands; 6 Bavarian Center for Battery Technology (BayBatt), Bayreuth, Germany; 7 Deutsches Elektronen-Synchrotron (DESY), Hamburg, Germany

**Keywords:** Batteries, Batteries

## Abstract

Solvent co-intercalation, that is, the combined intercalation of ions and solvent molecules into electrode materials, is an additional but much less explored lever for modifying the properties of metal-ion battery electrodes (metal = Li, Na, Mg, etc.). Knowledge on solvent co-intercalation is relatively scarce and largely limited to graphite anodes, for which in sodium-ion batteries, the co-intercalation of glyme molecules is fast and highly reversible. The use of co-intercalation for cathode active materials (CAMs) remains much less explored. Here we investigate for a series of sodium-layered sulfide CAMs (Na_*x*_MS_2_, M = Ti, V, Cr and mixtures) under which conditions solvent co-intercalation occurs and how this process impacts the phase behaviour, electrode breathing, redox potential and cycle life compared to ‘Na^+^-only’ intercalation. Co-intercalation is a complex process that can, for example, cause opposing fluxes, meaning that solvents intercalate into the CAMs while sodium ions simultaneously deintercalate. Co-intercalation leads to layered structures that can include different amounts of confined solvated ions, ions and unbound solvent molecules. It is an approach to designing structurally diverse, layered materials with potential applications for batteries and beyond.

## Abstract (web summary)

Solvent co-intercalation into graphite anodes for sodium-ion batteries is common; however, intercalation into cathodes is much less explored. Here, using operando experiments as well as theory, solvent co-intercalation in a range of layered sulfides is investigated.

## Main

Lithium-ion and sodium-ion batteries (LIBs, SIBs) typically rely on intercalation reactions, where lithium or sodium ions are stored in the layered structures of the electrodes and exchanged between them during charging and discharging^1–4^. The electrodes are separated by a liquid electrolyte in which the ions are solvated, that is, the ions carry a solvation shell. Intercalation of the ions from the electrolyte into the electrode hence requires desolvation^5^. Likewise, solvation occurs when the ions deintercalate from the electrodes. In some cases, incomplete stripping of the solvation shell allows solvent molecules to also intercalate into the electrodes^6^. This process is referred to as co-intercalation (ions and solvents jointly intercalate) and is typically seen as being detrimental because it leads to degradation of the electrodes^7,8^. In current LIBs, the solid-electrolyte interphase (SEI) prevents the co-intercalation of solvents^9^. Nevertheless, co-intercalation reactions can exhibit high reversibility and rapid kinetics, often enduring for thousands of cycles^10,11^. A prominent example is the co-intercalation of Na^+^ and diglyme (2G) into graphite electrodes^12–14^. It is important to realize that the co-intercalation of solvents provides a unique opportunity for designing electrode reactions^15^. For example, as the solvents become part of the electrode reaction itself, solvent co-intercalation allows a targeted modification of the electrode potential over few hundred millivolts depending on the type of co-intercalated solvent(s)^16^. Another advantage is that the charge-transfer resistance during intercalation, which is normally dominated by the energy needed to strip the solvation shell^17^, can be minimized or even bypassed, favouring high energy efficiency reactions and high rate capability^18^. Notably, despite their much larger size, the diffusion of ‘solvated Na^+^’ in graphite is faster than the diffusion of Li^+^ (refs. ^19,20^). A drawback of solvent co-intercalation is that the larger size of the solvated ion leads to larger electrode breathing, and in the case of graphite, a decrease in specific capacity by more than two-thirds compared with conventional intercalation and hence energy density. The need for excess electrolyte is considered a practical challenge too but electrolyte and electrode optimization is possible to minimize the amount of electrolyte required, for example, by bypassing the irreversible electrolyte consumption during SEI formation.

Although most co-intercalation studies focus on graphite anodes, co-intercalation in cathode active materials (CAMs) has received very little attention, and understanding of its properties and feasibility in batteries remains limited^21^. Interestingly, co-intercalation was recently demonstrated by Ferrero et al. and Park et al. for layered titanium disulfide (TiS_2_) which is able to take up Na^+^ along with 2G molecules^18,22^. Tchitchekova et al. showed co-intercalation in TiS_2_ for Mg^2+^ and Ca^2+^ with propylene carbonate (PC), but high temperatures ≥100 °C are required and cell cycling remains challenging^23^. Although these studies encourage the exploration of new electrode reactions, TiS_2_ is not a true CAM because it lacks sodium (or other alkali earth ions) from the start, which would be required from a commercial perspective to allow cell assembly in the discharged state^24^.

Therefore, this study addresses solvent co-intercalation reactions for a series of Na_*x*_MS_2_ (where M = Ti, V, Cr or mixtures) compounds using different solvents (2G, PC and an ethylene carbonate (EC)/dimethyl carbonate (DMC) mixture). The co-intercalation of Na^+^ in CAMs for SIBs is demonstrated, and a reaction mechanism indicating an opposite flux of ions and solvents is identified. Guided by theoretical calculations and experimental validation, an interlayer binding energy–interlayer free volume model is proposed to predict solvent co-intercalation in layered CAMs, where the co-intercalation behaviour is governed by phase structure, sodium content, transition metal/anion species and solvent properties.

## Co-intercalation investigation in P2-Na_*x*_TiS_2_

**Fig. 1:**
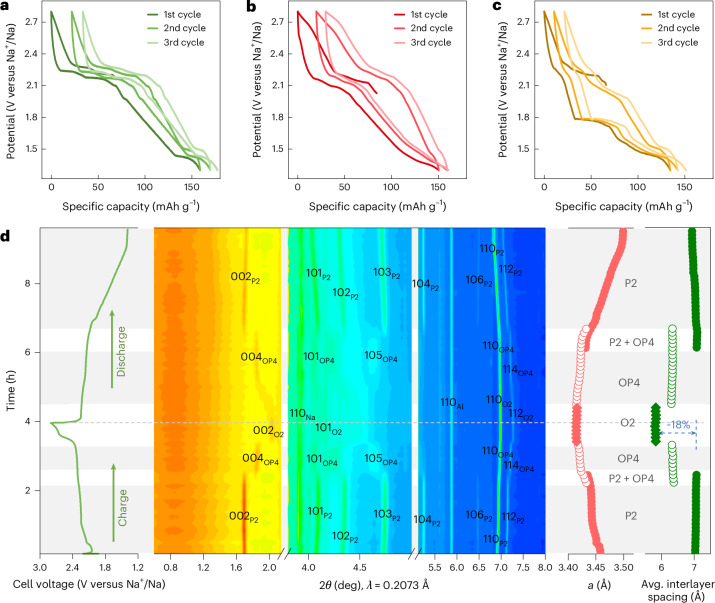
Electrochemical behaviour of P2-Na_*x*_TiS_2_. **a**–**c**, Voltage profiles of P2-Na_*x*_TiS_2_ in different electrolytes (1 M NaPF_6_ in EC/DMC (**a**), 0.5 M NaPF_6_ in PC (**b**) and 1 M NaPF_6_ in 2G (**c**)) for the first three cycles at 0.1 C. Different electrochemical behaviours are observed with the three studied electrolytes. Three-electrode measurements in Swagelok-type cells were carried out to minimize the influence of counter-electrode polarization. **d**, Operando XRD of P2-Na_*x*_TiS_2_ in 1 M NaPF_6_ in EC/DMC electrolyte during the first cycle at 0.125 C. Right side, pink symbols, corresponding *a* lattice parameter; right side, green symbols, corresponding average interlayer spacing; grey dahsed line, fully desodiated state. A typical intercalation reaction was detected when using the EC/DMC-based electrolyte. A reversible phase transition of P2–OP4–O2 is demonstrated in layered sulfide cathodes for the first time. Source data

Due to the elemental abundance of titanium, P2-type Na_*x*_TiS_2_ (*P*6_3_/*mmc* space group) synthesized by a high-temperature solid-state method was chosen as the prototype structure for detailed investigation (Supplementary Figs. 1–3 and Supplementary Tables 1 and 2). Defined potential steps are visible in the voltage profile with the EC/DMC-based electrolyte, while in the PC- and 2G-based electrolytes, the voltage plateaus smear out and the profiles become more sloping (Fig. 1a–c and Supplementary Fig. 4). Notably, an additional pair of plateaus located at 2.02 V (desodiation) and 1.77 V (sodiation) appears with 2G. The long-term cycling stability and high current density rate capability of P2-Na_*x*_TiS_2_ in 2G is better than that in EC/DMC (Supplementary Figs. 5 and 6). Interestingly, the appearance of this additional pair of plateaus (2.02/1.77 V versus Na^+^/Na) in 2G is very reversible and is maintained even after 2,000 cycles, while almost all plateaus with EC/DMC degrade gradually upon cycling. Additionally, a much narrower voltage gap (128 mV) after 200 cycles is found using 2G (Supplementary Fig. 7). In comparison, the PC-based electrolyte leads to an inferior capacity retention upon long-term cycling. The reaction mechanisms with the three electrolytes were therefore studied using synchrotron operando X-ray diffraction (XRD).

Figure 1d shows the operando XRD results for P2-Na_x_TiS_2_ in the EC/DMC-based electrolyte using an in-house designed operando cell (Supplementary Figs. 8 and 9). Charging to 2.3 V and 2.45 V versus Na^+^/Na resulted in two well-defined increases in the position of the 002/004 peak (2*θ* shifted from 1.69° to 1.83° and then to 2.04°), which is related to the successive formation of OP4 and O2 phases, as corroborated by Rietveld refinement results (Supplementary Figs. 10 and 11 and Supplementary Tables 3 and 4). Both the *a* lattice parameter and the average interlayer spacing (∼18%) decrease during the first desodiation process. Given the small overall changes in lattice parameters, a conventional intercalation mechanism seems likely.

**Fig. 2:**
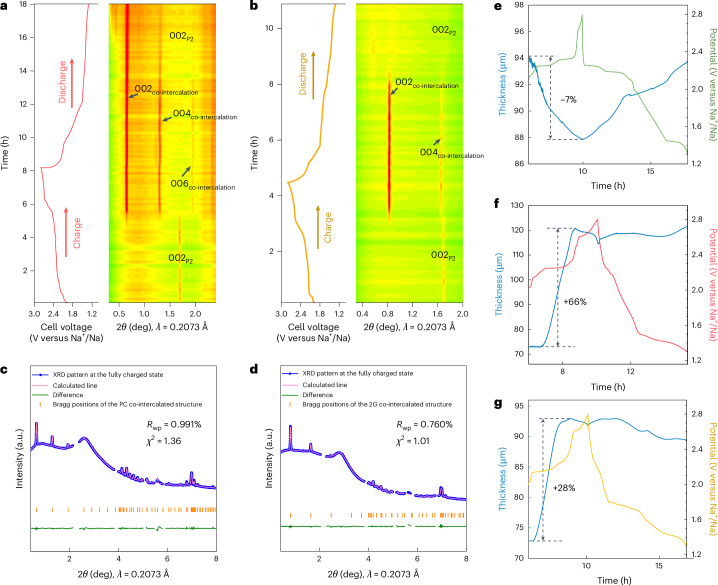
Large lattice expansion due to co-intercalation in PC- and 2G-based electrolytes. **a**,**b**, Operando XRD of P2-Na_*x*_TiS_2_ in 0.5 M NaPF_6_ in PC (**a**) and 1.0 M NaPF_6_ in 2G (**b**) electrolytes for the first cycle at 0.125 C. Compared to the EC/DMC electrolyte (Fig. 1d), a much larger expansion of the structure is observed in the PC and 2G electrolytes. **c**,**d**, Le Bail refinement of the XRD pattern in the fully desodiated state in the first cycle for PC-based (**c**) and 2G-based (**d**) electrolytes (aluminium and sodium peaks omitted). **e**–**g**, Three-electrode operando electrochemical dilatometry results for P2-Na_*x*_TiS_2_ in EC/DMC-based (**e**), PC-based (**f**) and 2G-based (**g**) electrolytes for the first cycle at 0.1 C. The blue curves show the thickness of the P2-Na_*x*_TiS_2_ electrode during cycling. Charging (desodiation) in the EC/DMC electrolyte leads to a minor contraction of the electrode, whereas a large expansion is found for the PC and 2G electrolytes. Source data

In contrast to EC/DMC, the phase behaviour of P2-Na_*x*_TiS_2_ cycled in PC- and 2G-based electrolytes is different. During the first desodiation process, the pristine P2 phase is maintained until the cell is desodiated to 2.43 V and 2.47 V versus Na^+^/Na for PC (Fig. 2a and Supplementary Fig. 12) and 2G (Fig. 2b and Supplementary Fig. 13), respectively. After this, the P2 phase disappears and a new phase emerges with the 002 peaks shifting to smaller angles, which is indicative of a substantial interlayer expansion. A Le Bail refinement of the XRD pattern in the fully oxidized state of the first cycle produces a perfect match with an expanded structure in both the PC (Fig. 2c) and 2G (Fig. 2d) cases. At these extreme interlayer spacings with PC and 2G electrolytes, which are 163% (18.3880 Å) and 106% (14.3458 Å) larger than the pristine P2 phase (6.9794 Å), respectively, PC- or 2G-solvated ions have ample space to fit into the structure (Supplementary Figs. 12c and 13c). Ex situ scanning electron microscopy (SEM) and XRD further confirm the formation of cracks and an expanded structure for the cases of PC and 2G (Supplementary Figs. 14–18), which is typical for a solvent co-intercalation mechanism. During subsequent sodiation, the expanded structure is maintained in both PC and 2G electrolytes, with only partial reappearance of the original P2 phase. However, the reversibility using 2G is better, indicating a greater structural flexibility. Furthermore, the 002-peak position using 2G (0.83° 2*θ*) is higher than when using PC (0.65° 2*θ*), indicating that the co-intercalation of PC molecules causes a 30% larger expansion than when 2G is used.

Operando electrochemical dilatometry (ECD) continuously probes the thickness change of the entire electrode and, as a complement to XRD, is sensitive to the formation of, or any change in, amorphous phases^25,26^. Using EC/DMC (Fig. 2e), the electrode thickness continuously decreases/increases during desodiation/sodiation with a change in the single-digit range, which is typical for intercalation reactions^27,28^. A quite different behaviour is found using PC (Fig. 2f) and 2G (Fig. 2g); in these electrolytes the electrode substantially expands during desodiation. We were initially very surprised at this behaviour because, during desodiation, sodium ions leave the structure and one would therefore expect a contraction rather than a substantial expansion of the electrode. The electrode expansion peaks before the end of desodiation at around 2.5 V versus Na^+^/Na and amounts to 66% and 28% for PC and 2G, respectively. Upon further desodiation to higher potentials, the electrode thickness slightly decreases again, that is, the electrode expands and contracts during the first desodiation process, indicating a very complex behaviour. Subsequently during the sodiation/desodiation process (Supplementary Fig. 19), the electrode remains expanded in PC and 2G, which aligns well with the operando XRD findings. These observations are understood to arise from solvent co-intercalation processes that occur in cells with electrolytes containing PC or 2G, where solvents are unexpectedly entering the structure as the ions are exiting during the initial desodiation.

## Mechanism of co-intercalation in layered sulfide cathodes

**Fig. 3:**
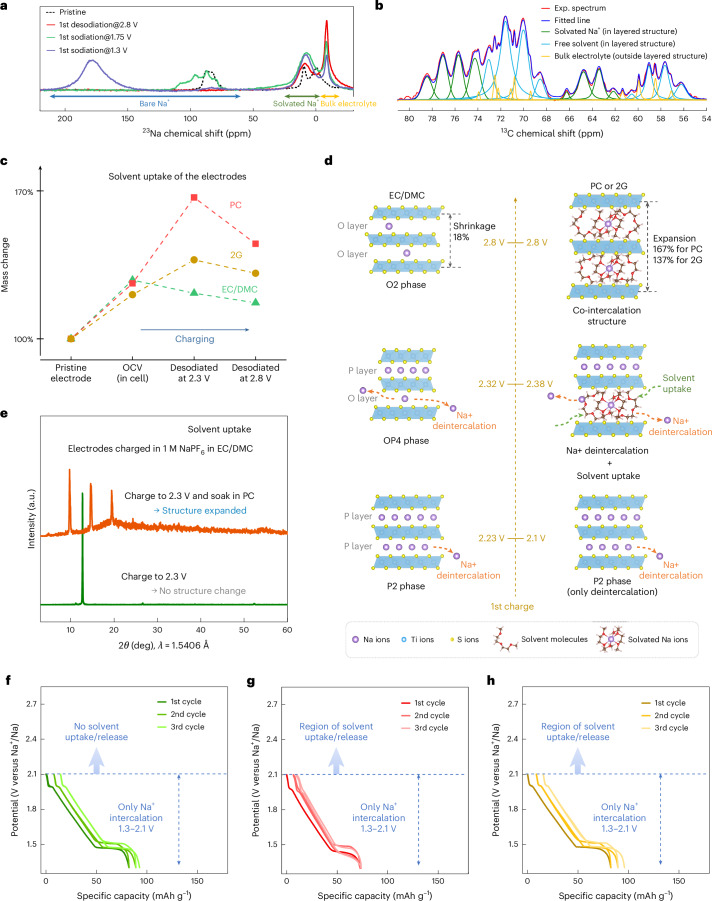
Mechanism of co-intercalation in layered sulfide cathodes. **a**, ^23^Na solid-state NMR spectra (20 kHz) of P2-Na_*x*_TiS_2_ at different SoCs in 1 M NaPF_6_ in 2G electrolyte. Paramagnetic interaction with Ti^3+^ centres result in signals between +50 and +200 ppm for intercalated Na^+^, with the remaining environments related to solvated Na^+^ in the interlayer space (+7 ppm) and bulk electrolyte (−8.6 ppm). **b**, ^13^C solid-state NMR spectrum of the P2-Na_*x*_TiS_2_ of the first desodiation at 2.8 V versus Na^+^/Na. Red, experimental spectrum; blue, sum of fitted lines; light blue, free solvent in the layered structure; orange, bulk electrolyte outside layered structure; green, solvated Na^+^ in layered structure. **c**, Relative mass of the P2-Na_*x*_TiS_2_ electrodes in the pristine state (before cell assembly), at OCV (electrodes are wetted), and after desodiation to 2.3 V and 2.8 V versus Na^+^/Na in the different electrolytes. **d**, Schematic diagram of solvent uptake and (de-)co-intercalation in P2-Na_*x*_TiS_2_ during charging. For EC/DMC, only deintercalation of Na^+^ occurs. For PC or 2G, Na^+^ deintercalation also takes place but simultaneous solvent intercalation occurs as soon as the sodium content in P2-Na_*x*_TiS_2_ becomes sufficiently low. The intercalated solvent prefers to interact with sodium ions in the layered structure, forming solvated sodium ions. Towards the end of desodiation, de-cointercalation, that is, combined deintercalation of Na^+^ and solvents, takes place. **e**, XRD patterns for a P2-Na_*x*_TiS_2_ electrode desodiated using an EC/DMC electrolyte (green line) and after subsequently soaking this electrode in PC for 48 h (orange line). The measurements show that the expansion is caused by an uptake of specific solvents such as PC and occurs by chemical diffusion. **f**–**h**, Voltage profiles of P2-Na_*x*_TiS_2_ in EC/DMC (**f**), PC (**g**) and 2G (**h**) electrolytes in a reduced voltage window of 1.3–2.1 V versus Na^+^/Na at 0.1 C (three-electrode measurements in Swagelok-type cells). The identical shapes demonstrate that solvent intercalation does not take place for high sodium contents in Na_*x*_TiS_2_ (*x* > 0.6). Source data

Detailed information on how the intercalated species (Na^+^ and 2G) interact with each other and with the host structure was obtained by ^23^Na and ^13^C solid-state NMR measurements at different states of charge (SoCs) and using P2-Na_*x*_TiS_2_ as host. The ^23^Na NMR spectra (Fig. 3a and Supplementary Figs. 20 and 21) provide evidence for both solvated Na^+^ (at +7 ppm)^20^ and bare Na^+^ (+50 to +200 ppm) occupying the interlayer space. The signal at –8.6 ppm is due to Na^+^ from the bulk electrolyte^29^. The peak area of solvated Na^+^ reaches a maximum at 1.75 V and remains similar at 1.30 V, suggesting saturation of co-intercalated Na^+^. After saturation, sodiation proceeds via bare Na^+^ intercalation, with pronounced shifts attributed to through-space pseudocontact effects^30^ from Ti^3+^ paramagnetic centres in Na_*x*_TiS_2_. This interaction also influences the ^13^C NMR spectra (Fig. 3b, Supplementary Figs. 22–26 and Supplementary Tables 5–8), revealing three distinct 2G environments: bulk electrolyte outside the layered structure, a paramagnetic shift assigned to solvated Na^+^ in the interlayer space, and broadened resonances assigned to free 2G solvent in the interlayer space. Following the initial co-intercalation of solvent, there are, at any SoC, always Na^+^, solvated Na^+^ and free solvents in the structure.

To investigate this further, the mass of the electrodes was monitored (Fig. 3c and Supplementary Fig. 27). Before any electrochemical driving force was applied, the increase in electrode mass was similar for all three electrolytes, attributable to electrode wettability at OCV. During desodiation, the trend for EC/DMC is as expected, that is, desodiation leads to a continuous reduction of the electrode mass. For 2G and especially for PC, however, a substantial mass increase is found after desodiation to 2.3 V versus Na^+^/Na, followed by a slight decrease upon further desodiation to 2.8 V versus Na^+^/Na. This change is well in line with the operando ECD and XRD results discussed above, that is, solvents diffuse into the crystal structure leading to a substantial increase in mass, while a slight decrease in electrode thickness was also observed in ECD when reaching high redox potentials. Overall, this suggests that three stages occur during desodiation (as illustrated in Fig. 3d and Supplementary Fig. 28): in stage 1, conventional deintercalation of Na^+^ occurs. Stage 2 begins when the sodium content drops below a critical threshold, at which point Na^+^ deintercalation continues concurrently with solvent intercalation. This defines a ‘co-intercalation with opposite flux’ mechanism, where both Na^+^ and solvent molecules coexist within the structure. In stage 3, both species are extracted (de-cointercalation), although a fraction of solvent may remain trapped within the host lattice.

Because free solvent molecules are electrically neutral, there is no electrochemical driving force for them to enter the P2-Na_*x*_TiS_2_ structure. Instead, it is likely that a chemical process or simply absorption is the cause. To prove this, the electrode desodiated to 2.3 versus Na^+^/Na in EC/DMC was disassembled from the cell and soaked in pure PC solvent for 2 days. This procedure surprisingly results in a substantial mass increase (150%) and an expanded structure, as evidenced by the corresponding XRD pattern (Fig. 3e). This confirms that the expansion of the P2-Na_*x*_TiS_2_ lattice and the electrode during desodiation in PC and 2G electrolytes is indeed the result of the chemical uptake of these solvents.

Notably, stage 2 only emerges when the cell is desodiated above a threshold potential. A reduced voltage window of 1.3–2.1 V versus Na^+^/Na is applied to test whether solvent uptake at lower voltages can be excluded. The identical voltage behaviours and ex situ XRD results (Fig. 3f–h and Supplementary Figs. 29 and 30) confirm that only sodium (de)intercalation takes place in the low-voltage regime in the three electrolytes, in contrast to the solvent uptake of PC and 2G when reaching a sufficiently high redox potential. Thus, a low sodium content (*x* < 0.6 in P2-Na_*x*_TiS_2_) is crucial for enabling the uptake of PC or 2G solvent to expand the structure (as illustrated in Fig. 3d and supported by Supplementary Fig. 31).

## Theoretical considerations

**Fig. 4:**
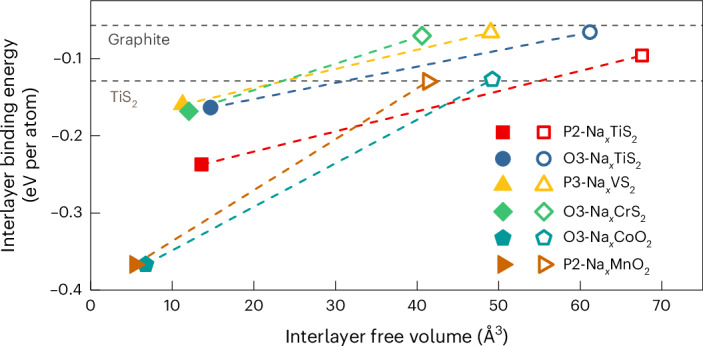
Interlayer binding energy and interlayer free volume of a variety of layered sulfides and oxides. The calculated interlayer binding energy and interlayer free volume of the optimized structures in their fully (*x* = 1, full symbols) and partially (*x* = 0.25, hollow symbols) sodiated states. The layered structures of the sulfide cathodes are much more open and easier to expand than those of the layered oxides. Moreover, the structures with low sodium content are much easier to expand and contain a larger degree of empty space for solvent uptake. The interlayer binding energies for graphite and TiS_2_ are added for comparison. Source data

Density functional theory (DFT) was used to further investigate the mechanism. Structural optimization using the Vienna Ab initio Simulation Package (VASP) was carried out on P2-Na_0.25_TiS_2_, expanded in the *c* direction to match the XRD observations, with a solvation shell placed between TiS_2_ layers (Supplementary Fig. 32). However, during structural optimization, the structure contracts as the solvents break away from the solvated ion and instead begin to coordinate to the sodium layer, that is, the solvents begin to coordinate to Na^+^ from the interior of the sodium layer, revealing that a single solvation shell is not sufficient to generate a stable expanded structure as observed in operando XRD. Thus, more solvent is required in the interlayer to maintain the expanded structure, and we have previously shown that there is a large amount of free solvent present in materials where solvent co-intercalation occurs^20,24,29,31^. However, in contrast to previously studied anodes, there is no electrochemical driving force for the solvent to enter the bulk of the material during a desodiation process. Rather, this process appears to occur by simple diffusion of solvent into the structure which subsequently expands. For such a process to occur, materials with weakly bound layers and large voids, that is, materials that have large interlayer free volumes and are easy to expand, should be prime candidates for solvent uptake. The interlayer binding energy and the interlayer free volume are thus proposed as vital parameters to study co-intercalation. Computing the interlayer binding energy and the interlayer free volume for several layered sulfides, and for two-layered oxide cathodes, shows that in general the layered sulfides (in both sodium-deficient and sodium-replete cases) are much easier to expand and contain more free volume than the layered oxides (Fig. 4 and Supplementary Fig. 33). Crucially, however, all the structures become much easier to expand and contain more free volume at low sodium content. The sodium-deficient layered sulfides require very low interlayer energies of 0.066–0.096 eV per atom to expand, comparable to graphite (0.057 eV per atom), and have very large interlayer free volumes. In particular, the free volume for P2-Na_0.25_TiS_2_ increases by more than 700% compared with P2-NaTiS_2_ and has the largest free volume. In comparison, TiS_2_, characterized by a higher interlayer energy of 0.129 eV per atom, exhibited no chemical uptake of PC or 2G solvents (Supplementary Figs. 34–37)^18^.

## Co-intercalation in other layered cathodes

**Fig. 5:**
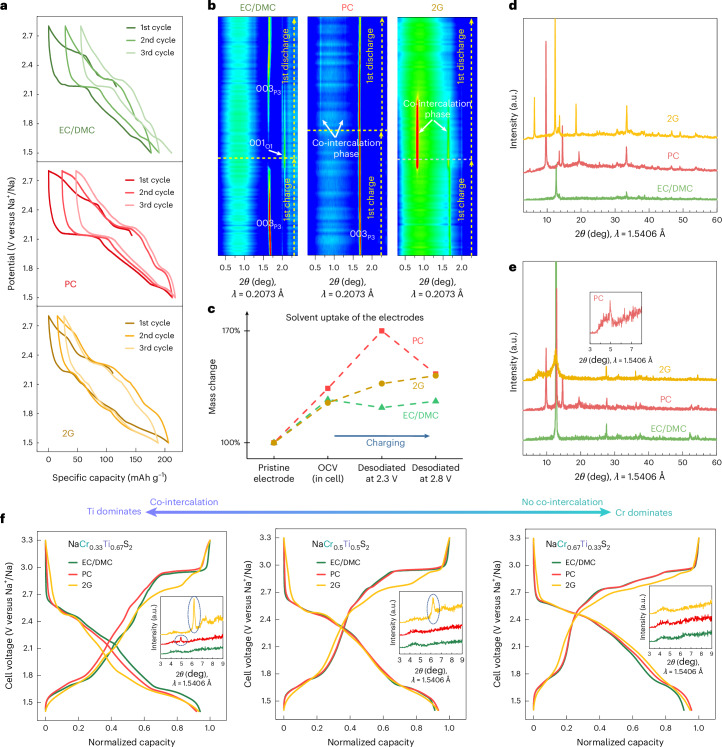
Co-intercalation in other layered sulfides. **a**, Voltage profiles of P3-Na_*x*_VS_2_ in EC/DMC, PC and 2G electrolytes at 0.1 C showing different behaviours. Three-electrode measurements in Swagelok-type cells were used to minimize the influence of counter-electrode polarization. **b**, Results from operando XRD of P3-Na_*x*_VS_2_ electrodes cycled in EC/DMC, PC and 2G at 0.125 C. Similar to P2-Na_*x*_TiS_2_, a large lattice expansion indicative of solvent intercalation occurs with PC and 2G. **c**, Relative mass of P3-Na_*x*_VS_2_ electrodes in the pristine state (before cell assembly), at OCV (electrodes are wetted), and after desodiation to 2.3 V and 2.8 V versus Na^+^/Na in the different electrolytes. The large mass increase for PC and 2G indicates solvent intercalation. **d**,**e**, Ex situ XRD patterns of O3-Na_*x*_TiS_2_ (**d**) and NaTi_0.5_V_0.5_S_2_ (**e**) for the first discharge at 2.19 V versus Na^+^/Na (sodiation) for different electrolytes. Inset **e**: enlarged region of 3°–8° of the XRD pattern of NaTi_0.5_V_0.5_S_2_ in the PC-based electrolyte. Both compounds form an expanded structure when using PC or 2G. **f**, Normalized voltage profiles (second cycle) and ex situ XRD patterns at 2.19 V versus Na^+^/Na (sodiation) in insets for NaCr_*y*_Ti_1−*y*_S_2_ (*y* = 0.33, 0.5 or 0.67) in different electrolytes. Blue circles in insets: the peak of the co-intercalation phase. The co-intercalation behaviour changes with the dominance of different transition metals (titanium or chromium). Source data

A generalization of the concept was explored by studying a series of layered sulfides, Na_*x*_MS_2_ (where M = Ti, V, Cr or mixtures). The P3-type Na_*x*_VS_2_, O3-type Na_*x*_TiS_2_ and P3-NaTi_0.5_V_0.5_S_2_ show similar characteristic features in their voltage profiles and phase behaviours during cycling, that is, co-intercalation occurs with the PC- and 2G-based electrolytes, but not when using EC/DMC (Fig. 5a–e and Supplementary Figs. 38–41). Notably, P3-Na_*x*_VS_2_ demonstrates improved cycling stability with the co-intercalation reaction (Supplementary Fig. 42). The interlayer spacing of various co-intercalated CAMs depends more on the intercalated solvent, as shown in Supplementary Fig. 43, than on the type of layered structure/compound. Upon solvent uptake, the interlayer forces between transition metal layers are weakened as the solvent molecules occupy the interlayer space. However, the voltage hysteresis of the layered CAMs with co-intercalation depends on both the electrolyte solvent and the type of the layered structure (Supplementary Figs. 44–46). The P3- or O3-type layered sulfides show a similar degree of voltage hysteresis for intercalation and co-intercalation processes. Equilibration measurements over a period of 160 h showed that there is no notable difference in equilibration voltage or self-discharge between the electrodes being intercalated and co-intercalated (Supplementary Fig. 47). Notably, an exception is found for the O3-type Na_*x*_CrS_2_, where no co-intercalation is found with the three different electrolytes (EC/DMC, PC and 2G; Supplementary Fig. 48). This is also the sulfide that has the lowest interlayer free volume in its sodium-deficient structure (Fig. 4). It therefore seems that the transition metal has a major influence on the ability for solvent intercalation to occur. In fact, we find that for mixed layered sulfides containing more than one transition metal, the behaviour depends on the ratio of the transition metals. For a series of NaCr_*y*_Ti_1−*y*_S_2_ compounds with y = 0.33, 0.5 and 0.67 (Fig. 5f and Supplementary Figs. 49 and 50), PC or 2G co-intercalation occurs when titanium dominates (*y* = 0.33), while only intercalation takes place when chromium dominates (*y* = 0.67). For an equal content of titanium and chromium (*y* = 0.50), however, solvent co-intercalation takes place in the case of 2G but not with EC/DMC or PC. Overall, the results indicate that solvent intercalation can occur in many (but not all) layered sulfides and depends on the type of solvent, the type of transition metal(s), and the sodium content, providing a large chemical playground for further studies. A summary of the co-intercalation behaviour of layered sulfide cathodes is given in Table 1. In comparison, the representative layered oxides, NaMnO_2_, P2-Na_0.7_CoO_2_, O3-NaCoO_2_ and NaNiO_2_, show only an intercalation reaction for the three different solvents (Supplementary Figs. 51–54). These observations are in good agreement with the DFT results shown in Fig. 4, which indicate that co-intercalation may well occur (next to graphite) in a variety of layered sulfides, while the lattice of layered oxides is probably too dense and rigid to allow for such a process. Layered oxides, even those with low sodium contents, exhibit relatively high binding energies (even higher than or comparable to TiS_2_) and in some cases the interlayer free volume is too low to favour the occurrence of the co-intercalation reaction. However, layered sulfide cathodes are capable of 2G or PC co-intercalation.

**Table 1:** Ability for co-intercalation reactions of layered sulfide cathodes

Compounds	Phase	Co-intercalation		
		EC/DMC	PC	2G
Na_*x*_TiS_2_	P2	×	✓	✓
Na_*x*_VS_2_	P3	×	✓	✓
Na_*x*_TiS_2_	O3	×	✓	✓
NaTi_0.5_V_0.5_S_2_	P3	×	✓	✓
Na_*x*_CrS_2_	O3	×	×	×
NaCr_0.67_Ti_0.33_S_2_	O3/P3	×	×	×
NaCr_0.5_Ti_0.5_S_2_	O3/P3	×	×	✓
NaCr_0.33_Ti_0.67_S_2_	O3/P3	×	✓	✓

The main solvent properties responsible for promoting solvent co-intercalation are the stability of the solvation shell (desolvation free energy), the size of the solvation shell and the oxidative/reductive stability of the electrolyte. The more stable the solvation shell, the higher the activation barrier for stripping the solvation shell and hence an increased likelihood of solvent co-intercalation^32^. Considering size effects, solvents that can form small solvation shells (such as water), or solvents with the ability to wrap around the cation (such as glymes), are more likely to co-intercalate as less energy is needed to expand the interlayer space of the layered structure (Supplementary Fig. 55)^24^. Finally, the electrolyte needs to be sufficiently stable to avoid decomposing before the electrode reaction is completed. Overall, layered sulfides show favourable properties for solvent co-intercalation because their redox potential is typically in a safe region (1.3–3.5 V) where many solvents are still stable and because the interlayer energy is relatively weak compared with, for example, layered oxides. The fact that PC can co-intercalate in some layered sulfides while EC cannot appears surprising considering their chemical similarity. However, it should be noted that this difference in behaviour has been known for many years in the context of lithium intercalation into graphite. Several possible explanations have been presented, such as the methyl group lending more flexibility to the PC molecule compared with EC^9,33,34^. However, it should also be noted that EC is solid at room temperature and is therefore typically mixed with other linear solvents such as DMC. This results in a less stable solvation shell, making co-intercalation in an EC-based electrolyte less likely. More importantly, the two solvents differ in their ability to form a stable SEI layer on graphite, with EC forming an SEI layer that effectively prevents solvent co-intercalation^35^.

## Conclusions

Solvent co-intercalation can be used to tune the properties of some layered CAMs for Na-ion batteries and depends on the specific CAM–solvent combination. For Na_*x*_MS_2_ (M = Ti, V, Cr or mixtures), intercalation occurs in EC/DMC electrolytes, while for M = Ti and V, co-intercalation occurs in PC and 2G electrolytes. Notably, while co-intercalation in graphite anodes typically lowers the specific capacity (around 110 mAh g^−1^ for co-intercalation compared with, for example, 372 mAh g^−1^ for lithium intercalation), the penalty for the CAMs studied here is very small. The process of co-intercalation in sulfide CAMs is found to be very complex and depends on the specific combination of CAMs, solvents and the SoC of the electrode. The interlayer binding energy and the interlayer free volume can be used as descriptors to predict whether co-intercalation can take place. Initial charging causes a mechanism with an opposite flux, meaning that when Na^+^ deintercalates, solvent molecules intercalate and expand the lattice once a threshold potential (≥2.1 V versus Na^+^/Na for P2-Na_*x*_TiS_2_) is exceeded. Depending on the SoC, the layered structure contains confined solvated ions, ions and unbound (‘free’) solvent molecules. Overall, this study shows that co-intercalation of ions and solvents into layered structures provides an alternative and versatile approach for designing materials.

## Methods

### Materials synthesis

The appropriate amounts of sodium sulfide (Na_2_S; Alfa Aesar), sulfur (Sigma Aldrich) and elemental transition metal (titanium, Alfa Aesar, 325 mesh, 99%; vanadium, abcr, 325 mesh, 99.5%; chromium, Sigma Aldrich, 100 mesh, 99.5%) were well ground and mixed with a mortar and pestle in an argon-filled glovebox (MBRAUN) with an oxygen and water content of <0.1 ppm. The mixture was then placed in a quartz tube which was sealed under vacuum and heated slowly to a target temperature in a muffle furnace (Nabertherm). After being kept at the target temperature for a few hours/days, the quartz tube was slowly cooled down or quenched in air. The target temperature for preparing P2-Na_*x*_TiS_2_ and P3-Na_*x*_VS_2_ was 750 °C, whereas that for O3-Na_*x*_TiS_2_ was 430 °C. The NaTi_*y*_V_1−*y*_S_2_, NaCr_*y*_Ti_1−*y*_S_2_ and Na_*x*_CrS_2_ were prepared according to previous literature methods^36,37^. After transferring the quartz tube into the glovebox, the quartz tube was broken and the obtained sample was ground. For the synthesis of layered oxide compounds, Na_2_O_2_ and CoO or NiO_2_ were used as precursors, and the compounds were synthesized at high temperatures (450 or 850 °C) in air or in an oxygen atmosphere^38,39^. Because the samples are air- and moisture-sensitive, all the processes are carried out in an argon atmosphere unless otherwise noted, and the quartz tubes were predried before use.

### Electrochemistry

Electrode preparation was completed in an argon-filled glovebox. Slurries consisting of 70:20:10 (wt%) mixtures of active material, C65 carbon black (MTI) and polyvinylidene fluoride (MTI) were cast onto carbon-coated aluminium foil (MTI) using a 250-μm-thick doctor blade (mtv messtechnik). The prepared film was then dried overnight at 60 °C under vacuum in a glass oven (BÜCHI, B585). The mass loading of the active material was between 0.8 and 2.6 mg cm^−2^. For electrolyte preparation, sodium hexafluorophosphate (NaPF_6_; E-lyte, >99%) was used directly without drying. EC (Sigma Aldrich, 99%), DMC (Sigma Aldrich, ≥99%), PC (Sigma Aldrich, 99.7%) and 2G (Sigma Aldrich, 99.5%) were dried with a mixture of 3-Å and 4-Å molecular sieves (Carl Roth) before use. The prepared electrolytes were stirred for at least 6 h before application.

Two-electrode measurements were done in coin cells (CR2032; MTI). For cell assembly, the prepared electrodes were used as the working electrodes, while 12-mm-diameter sodium (BASF) discs were used as the counter-electrodes. Two 16-mm-diameter glass microfibre filters (Whatman, GF/A) were used as separators. Three-electrode measurements were performed using Swagelok-type cells with sodium as both counter and reference electrodes and the prepared 12-mm-diameter electrodes as working electrode. All cells were assembled in an argon-filled glovebox with an oxygen and water content of <0.1 ppm. Galvanostatic cycling with potential limitation (GCPL) tests were performed with a BCS battery testing system (BioLogic) and a Neware battery testing system. All GCPL tests were performed with two-electrode coin cells unless the use of three-electrode cells is explicitly stated. The 1-C rate corresponds to a specific current of 0.2 A g^−1^. All electrochemical characterization measurements were performed at room temperature in a temperature-controlled room.

### Characterization

High-temperature in situ XRD was conducted on a Stoe StadiP diffractometer using a Stoe ST2K furnace. The diffractometer is equipped with monochromatic Mo Kα_1_ (*λ* = 0.709319 Å) radiation and with a MYTHEN 1K detector. A mixture of the reactants in an appropriate ratio was sealed in a 1-mm-diameter quartz capillary, and heated in the ST2K furnace at a rate of 1 °C min^−1^. Simultaneously, the in situ XRD patterns were collected. Patterns were recorded in the 2*θ* range of 2°–37° with a step size of 0.015° for 10 min at each temperature. The furnace has a rocking motion to improve powder averaging.

Ex situ XRD patterns of the electrodes were collected at room temperature on a Bruker D2 Phaser diffractometer equipped with Cu Kα radiation (*λ* = 1.5406 Å) at a voltage of 30 kV and a current of 10 mA, in step-scan mode with a step size of 0.02° (2*θ*) and step time of 0.1–1.0 s in a 2*θ* range of 3°–80°. The morphological information of the active materials was collected from scanning electron microscopy (SEM; Thermo Fisher Scientific, Phenom Pharos Desktop SEM). Inductively coupled plasma optical emission spectroscopy (ICP-OES) was performed with a Thermo Fisher system (ICAP 7000) using QTEGRA software.

Synchrotron radiation operando XRD was measured at the P02.1 beamline at DESY with a photon energy of 60 keV (photon wavelength, ∼0.2073 Å)^40^. All operando experiments were tested using an in-house designed two-electrode operando cell with 12-mm-diameter electrodes as working electrodes and 12-mm-diameter metallic sodium discs as counter-electrodes. Mylar foil with a thickness of 23 μm was used as the window material. Data were collected in transmission geometry using the VATEX CT4343 detector at a distance of 1,150 mm. Calibration was done using LaB_6_ (NIST 660c) powder. Data integration was done using pyFAI software^41^. For the synchrotron radiation ex situ XRD measurements, all powder materials were sealed in capillaries, mounted on a brass pin secured in a special holder, and loaded on a high-speed spinner. An open-source software General Structure Analysis System-II (GSAS II) was used for the Rietveld and Le Bail refinement^42^.

Operando ECD was measured with a three-electrode ECD-3-nano cell from EL-CELL. Metallic sodium was used as counter and reference electrodes, while 10-mm electrodes were used as working electrodes. About 250 μl of electrolyte was employed in each operando ECD cell. GCPL tests were performed on an SP-50 battery test station (BioLogic).

Single-pulse NMR spectra were acquired at room temperature on a Bruker Avance 400 NMR spectrometer (^23^Na and ^13^C Larmor frequencies of 105.86 and 100.63 MHz, respectively). NMR parameters used for magic-angle spinning (MAS) NMR data collection are shown in Supplementary Table 9. Zirconia rotors (2.5 mm) were spun at 20 kHz. NaF was used as a reference for ^23^Na (7.4 ppm) and adamantane powder for ^13^C (29.5 ppm). Samples for MAS NMR were prepared by disassembling the cycled coin cells in an argon-filled glovebox and filling the rotor with the cycled material (coin cells were cycled with powder materials instead of electrode sheets). The cycled material was not washed before being packed into the NMR rotors.

Solution-state ^13^C (not decoupled) NMR spectra were collected on a Bruker 500 MHz NMR spectrometer, on a 1 M NaPF_6_ sample in 2G and d_6_-THF.

The mass change experiments were conducted following the procedure developed by Åvall et al.^24^ Similar electrodes were cycled at constant current in coin cells to a specific potential and held for a minimum of 6 h. The cells were taken to an argon-filled glovebox and were disassembled. Immediately after the cell was opened, the electrodes were carefully peeled from the separator and placed in a weighing boat to measure the mass on a Pioneer PX225D balance with an accuracy of 0.01 mg.

### Simulation and theoretical calculation

Complete solvation shells of [Na:EC_6_]^+^, [Na:PC_6_]^+^ and [Na:2G_2_]^+^ were optimized using the Gaussian 16 suite at the B3LYP/6-311-G(d,p) level of theory, with the SMD implicit solvent model, and a subsequent frequency calculation was carried out to ensure the optimized structure is a local minimum in the energy landscape^43–47^. These optimized solvation shells were placed in the interlayer of Na_0.25_TiS_2_ with an interlayer distance set to the interlayer distance found from XRD. Structural optimization was then carried out with VASP 5.2 using the Perdew–Burke–Ernzerhof functional, with van der Waals dispersion correction through the Tkatchenko–Scheffler method, and a plane-wave energy cut-off of 540 eV (refs. ^48–53^). The ionic positions and unit cell vectors were allowed to relax and the energies and charge densities were computed with a 111 *Γ*-centred *k*-point mesh. Due to the large number of atoms in the structures, converged structures using a larger number of *k*-points could not be obtained. Instead, one of the solvation shells was removed and only a single layer was expanded, which allowed convergence of structures with a 444 *Γ*-centred *k*-point mesh.

The starting structures of the layered sulfides (Na_*x*_TiS_2_ (P2), Na_*x*_TiS_2_ (O3), Na_*x*_VS_2_ (P3) and Na_*x*_CrS_2_ (O3)) and oxides (Na_*x*_CoO_2_ (O3) and Na_*x*_MnO_2_ (P2)) were taken from the materials database^54^, with *x* = 1 or 0.25. Four possible starting locations for the sodium cations in NaTiS_2_ were investigated with respect to *k*-point sampling and energy cut-off (Supplementary Fig. 56). All choices of *k*-point sampling and cut-off energy show that a starting configuration where the sodium cations are not placed directly underneath titanium are preferable, and this structure was chosen as the basic structure used for interlayer energy calculations. Moreover, the energy converged with respect to *k*-point sampling and plane-wave energy cut-off using an 884 *Γ*-centred *k*-point mesh with a cut-off of 500 eV. Similarly, the convergence with respect to *k*-point sampling and energy cut-off was tested for the expanded NaTiS_2_ structure, yielding the same result. Consequently, an energy cut-off of 500 eV and an 884 *Γ*-centred *k*-point mesh was chosen for all further calculations. For the partially sodiated structures, the shortest distance between sodium ions inside the unit cell was chosen because these produced structures with the lowest energies. The following VASP-recommended, projector-augmented wave potentials where used: C, O, Na_pv, S, Ti_sv, V_sv, Cr_pv, Mn_pv, Co.

The interlayer binding energy *E*_IB_ was computed as

$${E}_{\mathrm{IB}}=\frac{{E}_{\mathrm{opt}}-{E}_{\exp }}{n}$$

where *E*_opt_ is the energy of the optimized unit cell, *E*_exp_ is the energy where the interlayer distance of one layer of the optimized unit cell was increased by 20 Å, and *n* is the number of atoms in the unit cell. The free volume inside the optimized structures was computed as

$${V}_{\mathrm{free}}=d\sqrt{{\rm{||}}\vec{a}\times \vec{b}{\rm{||}}}-{V}_{\mathrm{Na}}-m{V}_{\mathrm{S/O}}$$

where *d* is the distance between the sulfur/oxygen layers, $\vec{a} \times \vec{b}$ is the cross product of the unit cell vectors $\vec{a}$ and $\vec{b}$, $\sqrt{{||}\vec{a}\times \vec{b}{||}}$ is the area of the parallelogram spanned by $\vec{a}$ and $\vec{b}$, *V*_Na_ is the volume of sodium based on its ionic radius (1.16 Å) and *V*_S/O_ is the volume of a sulfur/oxygen atom based on their ionic radii (1.70 Å and 1.26 Å, respectively)^55^; *m* is 1 for the fully sodiated structures and 4 for the partially sodiated structures, that is, those with 0.25 sodium atoms per transition metal.

## Online content

Any methods, additional references, Nature Portfolio reporting summaries, source data, extended data, supplementary information, acknowledgements, peer review information; details of author contributions and competing interests; and statements of data and code availability are available at 10.1038/s41563-025-02287-7.

## Supplementary information

Supplementary Information Supplementary Figs. 1–56 and Tables 1–9.

## Source data

Source Data Fig. 1 Electrochemical data plotted in Fig. 1a–c and operando XRD data plotted in Fig. 1d.

Source Data Fig. 2 Operando XRD data and refinement results plotted in Fig. 2a–d and operando dilatometry data plotted in Fig. 2e–g.

Source Data Fig. 3 Solid-state NMR data plotted in Fig. 3a, b, mass measurement data plotted in Fig. 3c, XRD data plotted in Fig. 3e, and electrochemical data plotted in Fig. 3f–h.

Source Data Fig. 4 Theoretical calculation data plotted in Fig. 4.

Source Data Fig. 5 Electrochemical data plotted in Fig. 5a, f, XRD data plotted in Fig. 5b, d, e, and mass measurement data plotted in Fig. 5c.

## Data Availability

The data that support the findings of this study are present in the paper and in the Supplementary Information. Computational data are available via figshare at 10.6084/m9.figshare.29143679 (ref. ^56^). Source data are provided with this paper.
