# Supplementary material for: Solvent co-intercalation in layered cathode active materials for sodium-ion batteries
Source: Nat Mater. 2025 Jul 18;24(9):1441–9. doi: 10.1038/s41563-025-02287-7 (PMC12404988; doi:10.1038/s41563-025-02287-7)
Supplement: Supplementary file 1 — Supplementary Figs. 1–56 and Tables 1–9. [file 41563_2025_2287_MOESM1_ESM.pdf]

nature materials

Supplementary information https://doi.org/10.1038/s41563-025-02287-7

Solvent co-intercalation in layered cathode active materials for sodium-ion batteries

In the format provided by the authors and unedited

Table of Contents

Supplementary Figures **2–53**

Supplementary Tables **54–60**

References **61–62**

Supplementary Figures

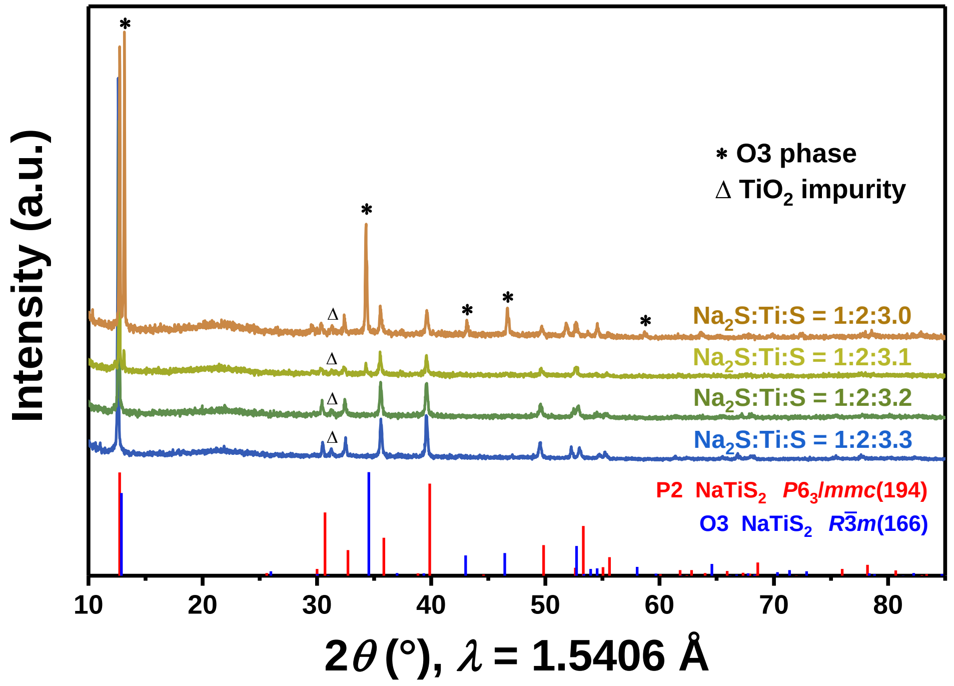

Supplementary Fig. 1 X-ray diffraction (XRD) patterns of the P2-Na_xTiS_2 compounds synthesized by using different molar ratios of the precursors.

The P2-Na_xTiS_2 compound was prepared by solid-state synthesis using Na_2S, Ti, and S as precursors. Due to the volatility of sulfur at high temperature, some optimization of the precursor ratio was required to obtain single-phase P2-Na_xTiS_2. Supplementary Fig. 1 shows XRD patterns after heat treatment of different precursor molar ratios (Na_2S:Ti:S = 1:2:z, z=3.0, 3.1, 3.2, and 3.3) at 750 °C. A secondary O3 phase with *R*-3*m* space group appeared when the molar ratio of S was 3.0 or 3.1. However, when the proportion of S was increased to 3.2 or 3.3, the P2-Na_xTiS_2 compound with a single P2 phase (space group: $P6_3/mmc$) was obtained, indicating that the structure of the final phase is very sensitive to the S content in the precursor mixture. Therefore, in order to successfully synthesize Na_xTiS_2 with a single P2 phase, the sulfur content should be at least 3.2, which exceeds the stoichiometric ratio (Na_2S:Ti:S = 1:2:3). Another sensitive factor for the preparation of layered sulfides is the heat treatment temperature (as is also well known for layered oxides).[1] A more detailed view on how the heat treatment temperature affects the structure was obtained through *in-situ* XRD, see Supplementary Fig. 2a. As can be observed, the formation of intermediate phases already occurs below 400 °C. From around 420 °C, the precursors react to form a mixture of O3 and O'3 layered sulfides with the former being the dominant phase, similar to what occurs in layered oxides.[1,2,3] With a further increase in temperature, the O3 and O'3 phases disappear (at about 510 °C) and the pure P2 phase forms and remains up to the maximum temperature of 800 °C. The results indicate that phase pure P2-Na_xTiS_2 is obtained from 600 °C and that it remains stable to at least 800 °C. Supplementary Fig. 2b shows the crystal structure of Na_xTiS_2 with a P2 phase, where sodium has a prismatic coordination within the sulfur anions framework packed with ABBA stacking. Rietveld refinement (Supplementary Fig. 2c and Supplementary Table 1) reveals that the data

of P2-Na_xTiS_2 can be fitted with a structural model using the $P6_3/mmc$ space group with unit cell parameters of a=b=3.462 Å and c=13.921 Å. The sample also contains a small amount of TiO_2, stemming from some oxygen contamination when evacuating the quartz tube. The inductively coupled plasma optical emission spectroscopy (ICP-OES) result in Supplementary Table 2 confirms the Na content in the pristine P2-Na_xTiS_2 (x=0.94). It should be noted that the P2 phase in the Ti-based layered sulfide is a high-temperature stable and Na-full phase, which is different from the Na-deficient P2-type layered oxide cathodes.[4] In contrast, the initial charge capacities in Fig. 1a–c indicate that a Na content of about x=0.5 is deintercalated during charging. The difference is due to the limited voltage window (not all Na is deintercalated) and due to the presence of an amorphous Na impurity (e.g. sodium polysulfide) as indicated by solid-state NMR.

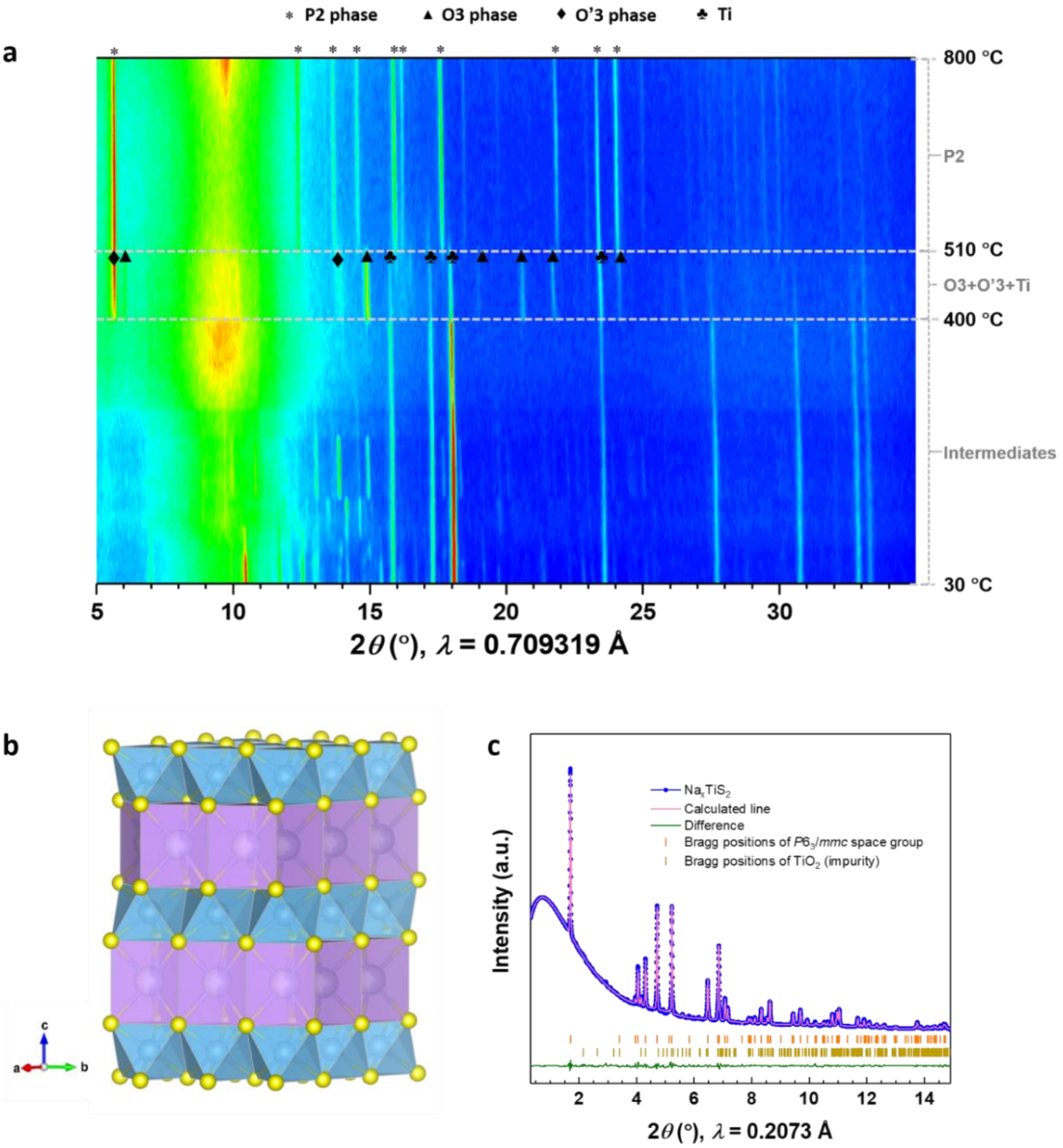

Supplementary Fig. 2 (a) *In-situ* XRD patterns of solid-state synthesis of P2-Na_xTiS_2. The "*", "♦", "▲", and "♣" symbols refer to the characteristic peaks of the P2, O'3, O3, and Ti phases, respectively. (b) Crystal structure of P2-Na_xTiS_2 with $P6_3/mmc$ space group (ICSD 10076). (c) Rietveld refinement of the XRD pattern of the P2-Na_xTiS_2 compound. The blue dot-line, pink line, green line, orange tick bars, and yellow tick bars represent the observed pattern, calculated

line, difference line, Bragg positions of the $P6_3/mmc$ space group, and Bragg positions of TiO_2, respectively.

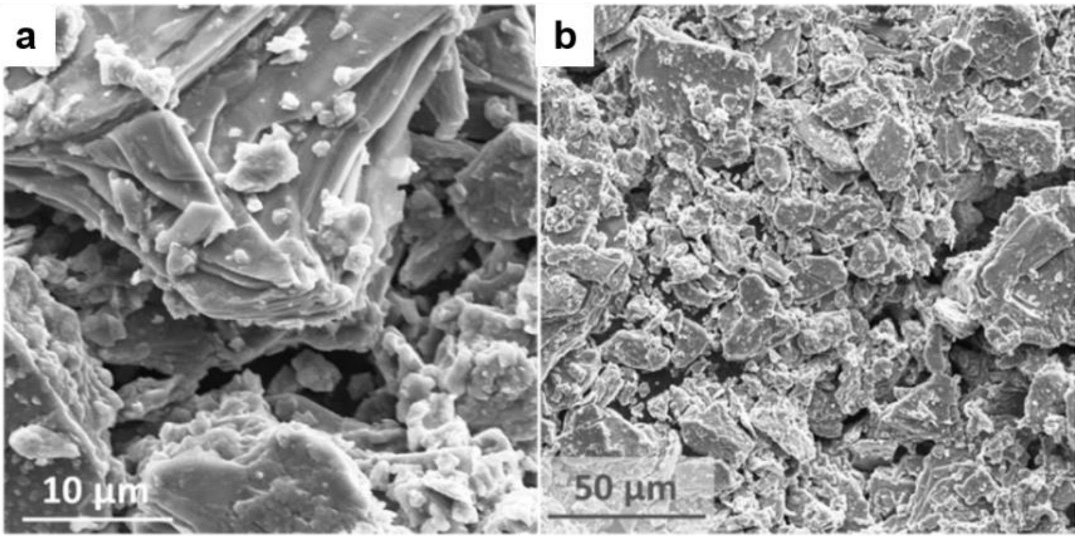

Supplementary Fig. 3 SEM images of P2-Na_xTiS_2. The particle morphology is typical for solid-state synthesis approach, i.e., particles of irregular shapes and sizes are found. The layered morphology can be clearly seen in Fig. a.

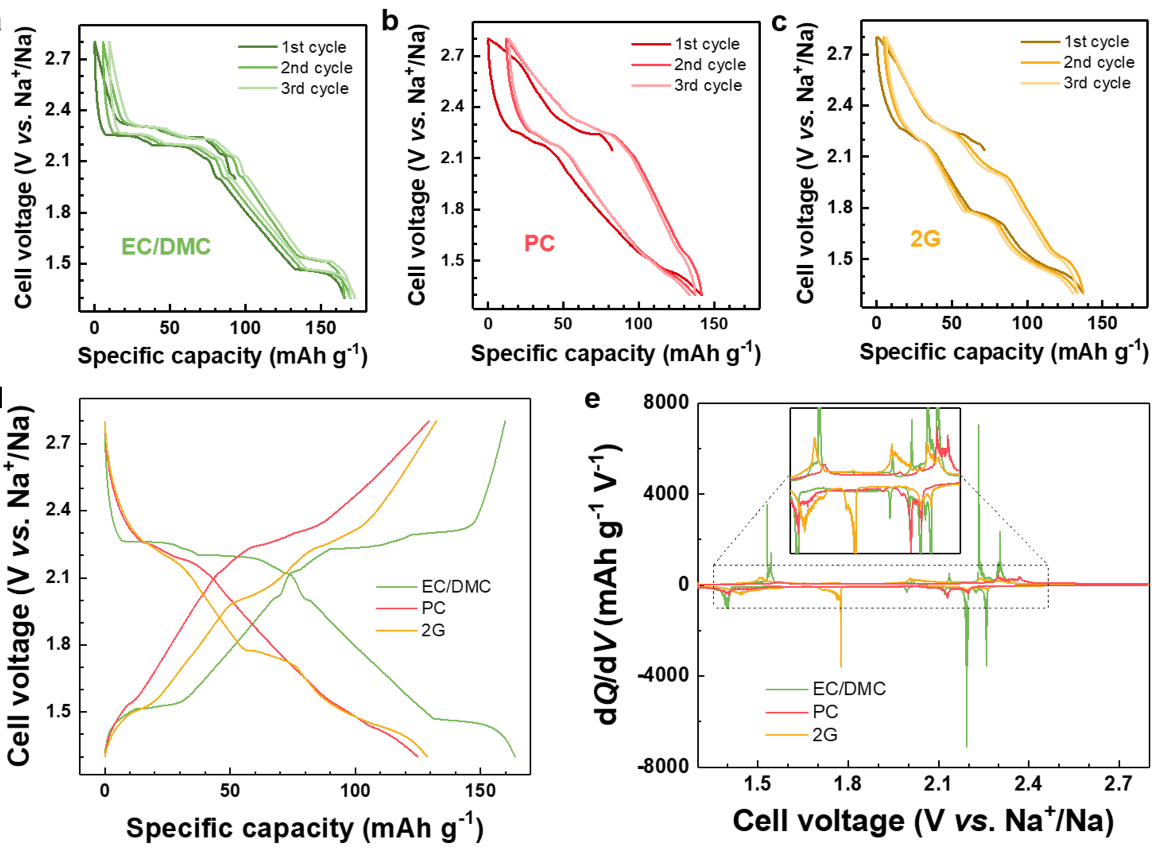

Supplementary Fig. 4 (a–c) Voltage profiles of P2-Na_xTiS_2 in different electrolytes (1 M $NaPF_6$ in EC/DMC, 0.5 M $NaPF_6$ in PC, and 1 M $NaPF_6$ in 2G) for the first three cycles at 0.1C. About 0.85 and 0.7 Na^+ per formula unit can be (de)intercalated in the EC/DMC- and PC (or 2G)-based electrolytes, respectively. Voltage profiles of the P2-Na_xTiS_2 in different electrolytes in the second cycle (d) and the corresponding differential voltage (d*Q*/d*V*) plots (e) at 0.1C with a voltage window of 1.3–2.8 V *vs*. Na^+/Na.

Supplementary Fig. 4a–d shows the voltage profiles for three different electrolyte solutions, 1 M $NaPF_6$ in EC/DMC and 2G, and 0.5 M $NaPF_6$ in PC between 1.3–2.8 V *vs*. Na^+/Na in two-electrode coin cells. A complex multi-step behavior can be observed for all three electrolytes, which is indicative for phase transitions. This is typical for many layered oxides and sulfides and has also been reported for Na_xTiS_2. Here, however, we find that the electrolyte solvent has a major influence on the voltage profile. In the standard 1 M $NaPF_6$ EC/DMC electrolyte, several clearly distinct potential steps are visible in the high voltage region (2.0–2.4 V *vs*. Na^+/Na), as well as a well-defined plateau at lower potentials (1.45 V *vs*. Na^+/Na). When PC is used as the electrolyte solvent instead of EC/DMC, the voltage plateaus become much less distinct, and a more sloping behavior is observed. Moreover, the reversible capacity in the PC-based electrolyte (129 mAh g^{-1}) is lower than that in the EC/DMC electrolyte (168 mAh g^{-1}) during the second cycle. For the 2G-based electrolyte, the electrochemical behavior of P2-Na_xTiS_2 changes again compared to the previous two electrolytes. The profile in 2G resembles that of the PC-based electrolyte, except for an extra pair of plateaus located at 1.77 and 2.02 V *vs*. Na^+/Na for the sodiation and desodiation process, respectively. These extra pair of plateaus were also observed for the TiS_2 electrode in the 2G-based electrolyte in our previous work, and were due to co-intercalation of solvent molecules.[5] About 0.85 and 0.7 Na^+ per formula unit

can be (de)intercalated in the EC/DMC- and PC (or 2G)-based electrolytes, respectively. The slightly lower amount of (de)intercalated Na^+ in PC and 2G might be due to the polarization or/and the solvent co-intercalation. The multi-step behavior can also be seen from the differential voltage (d*Q*/d*V*) plots in Supplementary Fig. 4e. In this plot, the additional redox plateaus for 2G can be seen as peaks at 1.77 V and 2.02 V *vs*. Na^+/Na. Moreover, the plot also underlines the fact that the most well-defined behavior is found for the EC/DMC electrolyte (the more-well defined the redox plateau, the more intense the peaks).

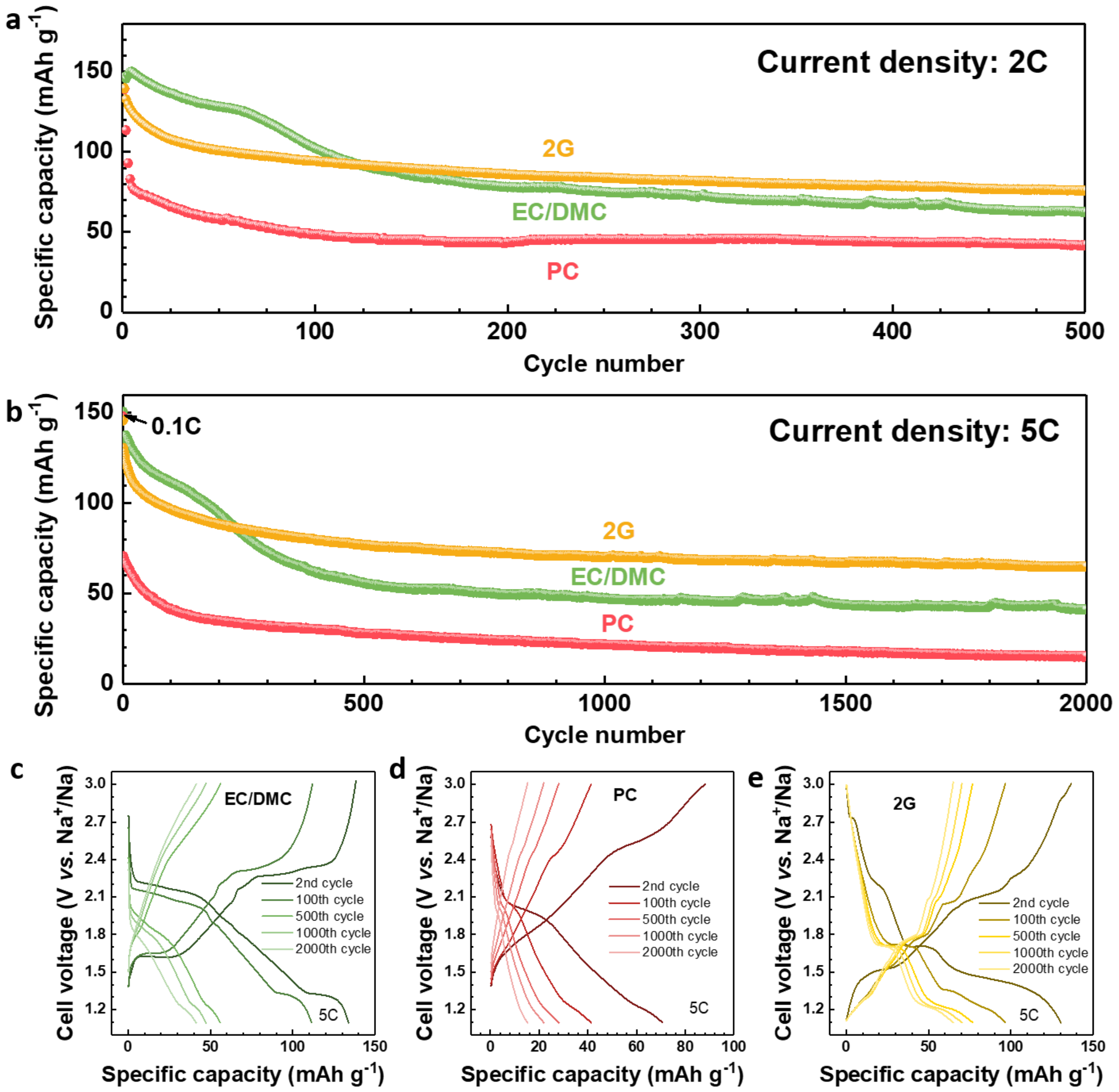

Supplementary Fig. 5 Long-term cycling stabilities of P2-Na_xTiS_2 in different electrolytes at current densities of 2C (a) and 5C (b). (c–e) Voltage profiles of P2-Na_xTiS_2 in different electrolytes for the 2nd, 100th, 500th, 1000th, and 2000th cycles at 5C. 1C corresponds to 0.2 A g^{-1}. The electrode cycled with the 2G-based electrolyte shows much better cycling stability than that in the EC/DMC case.

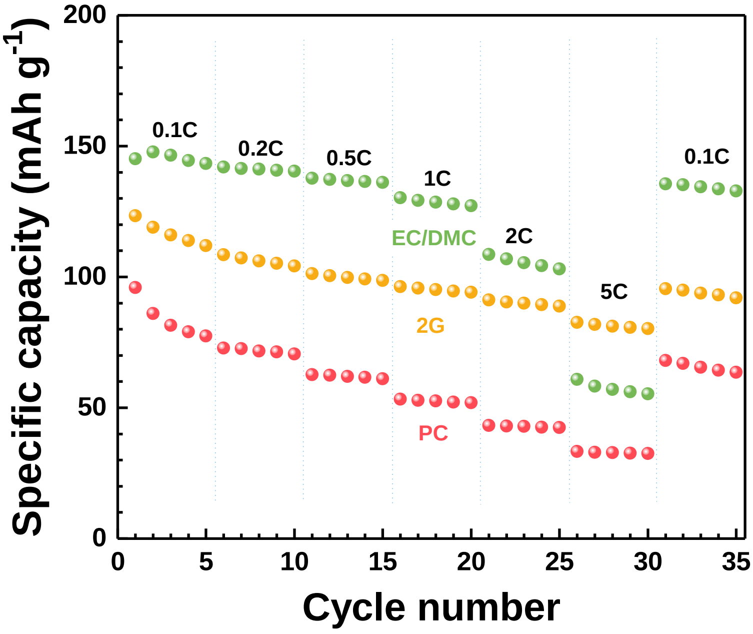

Supplementary Fig. 6 Rate capabilities of P2-Na_xTiS_2 in different electrolytes. The rate capability was conducted at 0.1C, 0.2C, 0.5C, 1C, 2C, 5C, and 0.1C with a voltage window of 1.3–2.8 V *vs*. Na^+/Na. 1C corresponds to 0.2 A g^{-1}.

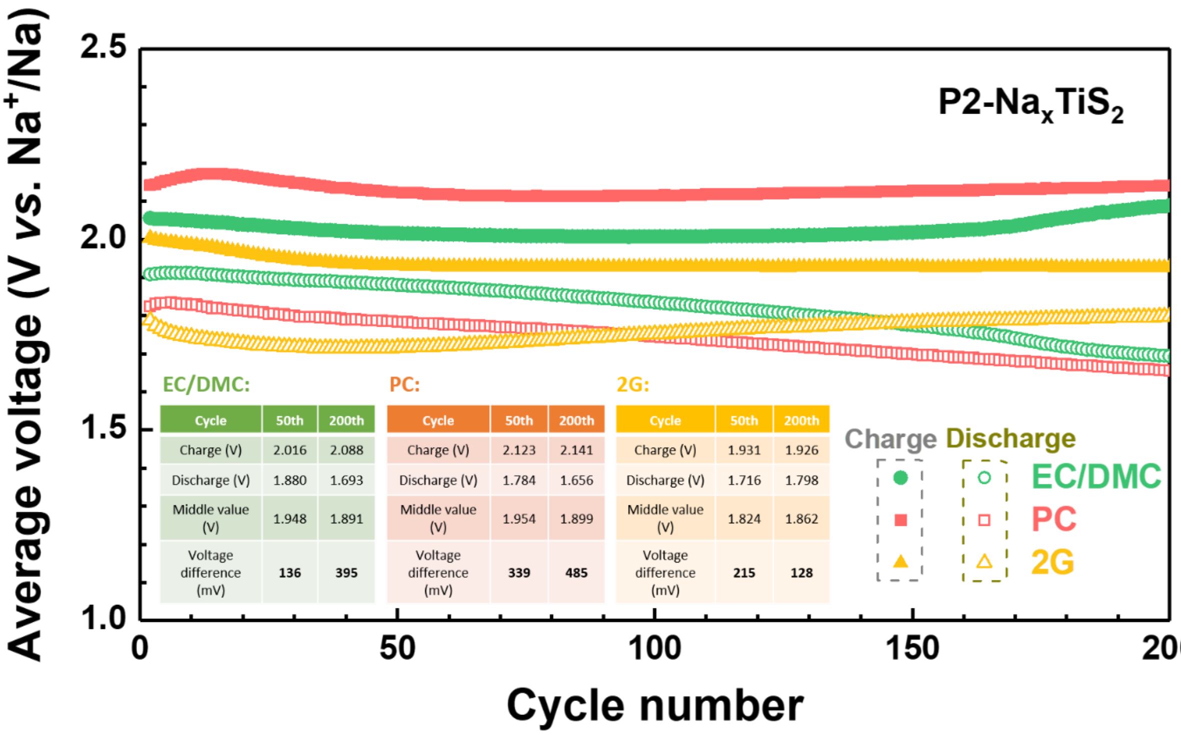

EC/DMC:

Cycle	50th	200th
Charge (V)	2.016	2.088
Discharge (V)	1.880	1.693
Middle value (V)	1.948	1.891
Voltage difference (mV)	136	395

PC:

Cycle	50th	200th
Charge (V)	2.123	2.141
Discharge (V)	1.784	1.656
Middle value (V)	1.954	1.899
Voltage difference (mV)	339	485

2G:

Cycle	50th	200th
Charge (V)	1.931	1.926
Discharge (V)	1.716	1.798
Middle value (V)	1.824	1.862
Voltage difference (mV)	215	128

Supplementary Fig. 7 Charge (solid symbol) and discharge (hollow symbol) average voltage of P2-Na_xTiS_2 in three different electrolytes at 0.1C. The tables inside the figure summarize the charge/discharge voltages, middle voltage value, and the voltage difference between charge and discharge voltages for the 50th and 200th cycles.

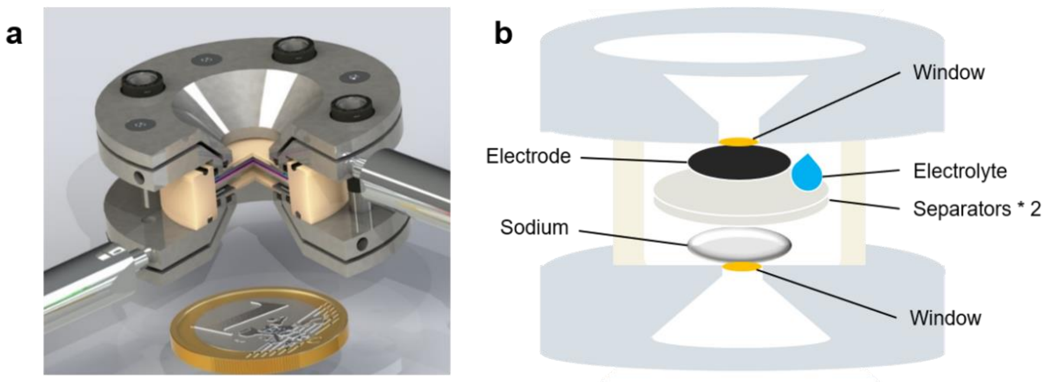

Supplementary Fig. 8 In-house designed cell for *operando* measurements. A Mylar foil (Goodfellow Cambridge Ltd.) with a thickness of 23 µm was used as the window material.

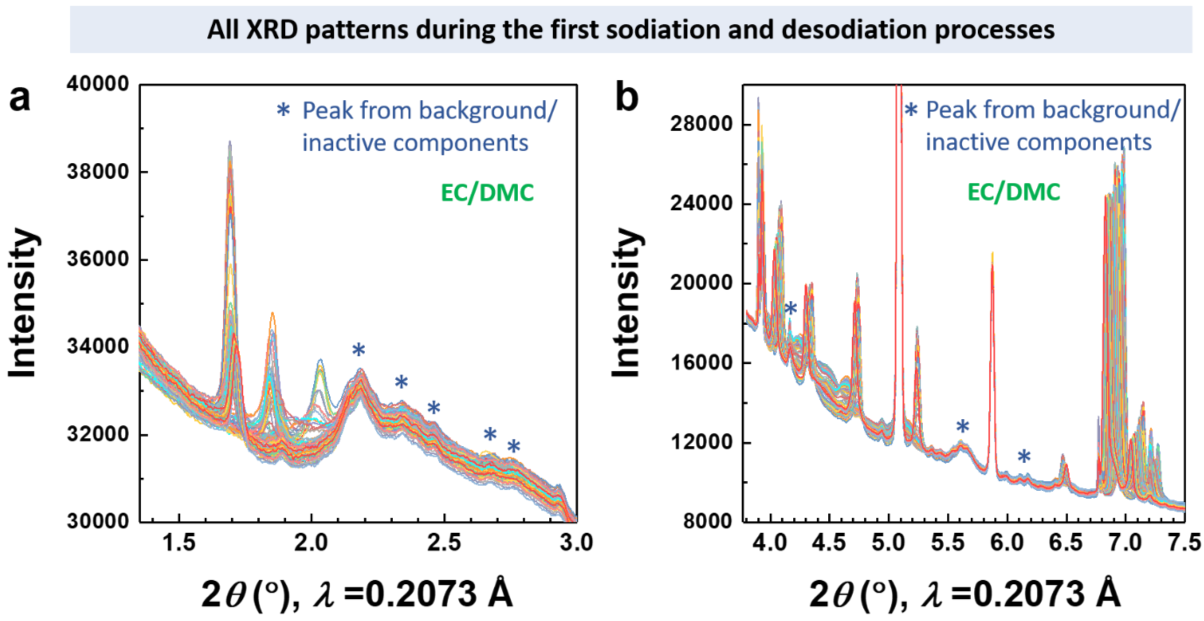

Supplementary Fig. 9 *Operando* XRD patterns of P2-Na_xTiS_2 in 1.0 M $NaPF_6$ in EC/DMC electrolyte for the first cycle. All patterns are included in both figures, where (a) includes the 2θ range between 1.35–3.0º, while (b) includes the 2θ range between 3.5–7.5º.

As shown in Supplementary Fig. 9, peaks assigned to electrochemically inactive components of the operando cell and/or the background are marked with blue stars. These can be traced back to the Mylar foil windows and the glass fiber separators, for example.

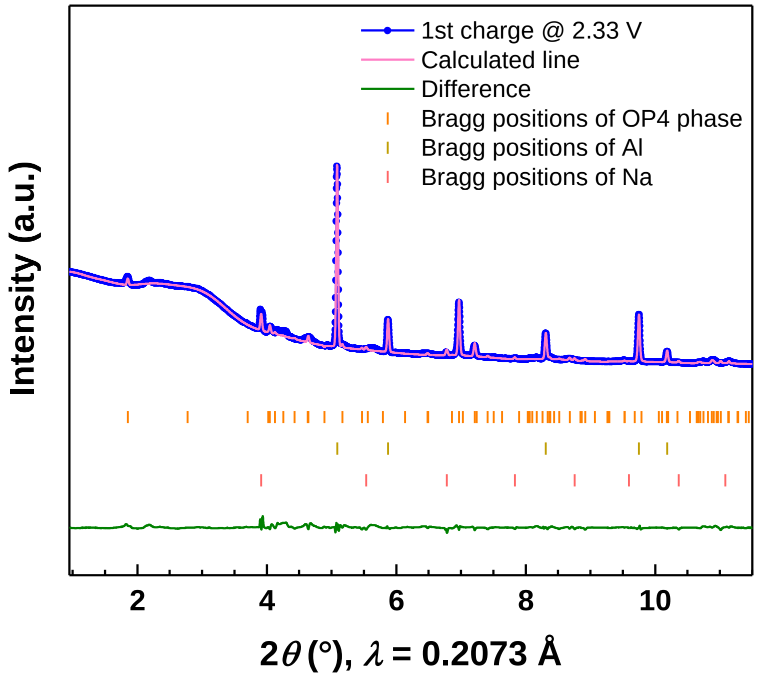

Supplementary Fig. 10 Rietveld refinement of the XRD pattern of the P2-Na_xTiS_2 electrode when desodiated to 2.33 V *vs*. Na^+/Na for the first cycle. The blue dot-line, pink line, green line, and tick bars represent the observed pattern, calculated line, difference line, and Bragg positions, respectively.

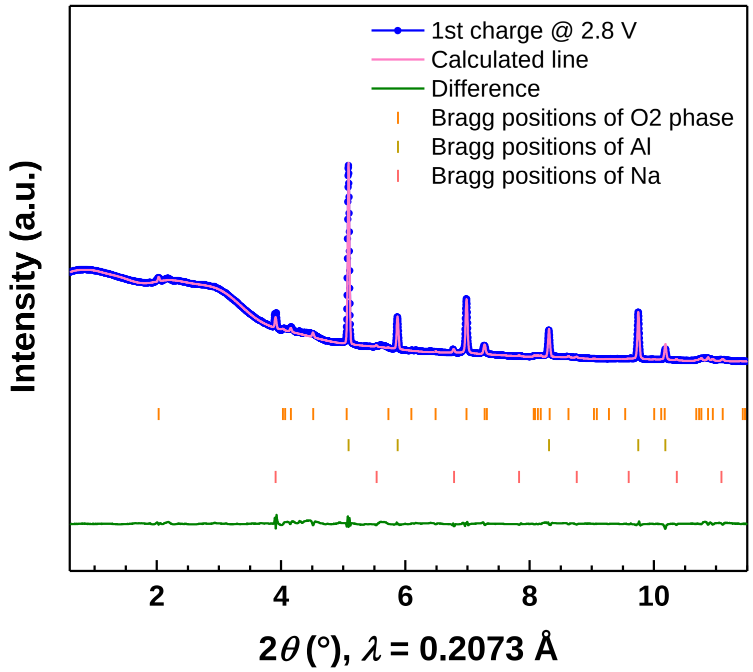

Supplementary Fig. 11 Rietveld refinement of the XRD pattern of the P2-Na_xTiS_2 electrode at fully desodiated state for the first cycle. The blue dot-line, pink line, green line, and tick bars represent the observed pattern, calculated line, difference line, and Bragg positions, respectively.

The sequence of phase transitions occurring during the first desodiation can be summarized as: P2 phase→P2+OP4 phases→OP4 phase→O2 phase. It should be pointed out that the observed phase transitions of P2-Na_xTiS_2 in the EC/DMC-based electrolyte, i.e., P2 phase→P2+OP4 phases→OP4 phase→O2 phase, are quite different from P2-based layered oxides. Mn-containing compounds typically show a direct transition from the P2 phase to either an O2 phase, [6,7,8] or an OP4 (or a "Z") phase,[9,10] or remain as a P2 phase[11] in the high-voltage region. In the P2-Na_xTiS_2, however, the P2 phase experiences an OP4 intermediate before transforming to an O2 phase in the high-voltage region. Although similar findings have been reported for $Na_{0.67}[Ni_{0.17}Mn_{0.5}Fe_{0.33}]O_2$ and $Na_{0.67}[Li_{0.1}Mn_{0.53}Fe_{0.37}]O_2$,[9, 12] where Na ions are (de)intercalated with the formation of a "Z"-phase intergrowth when the P2 transforms to O2, a final O2 phase is not reached in both cases.

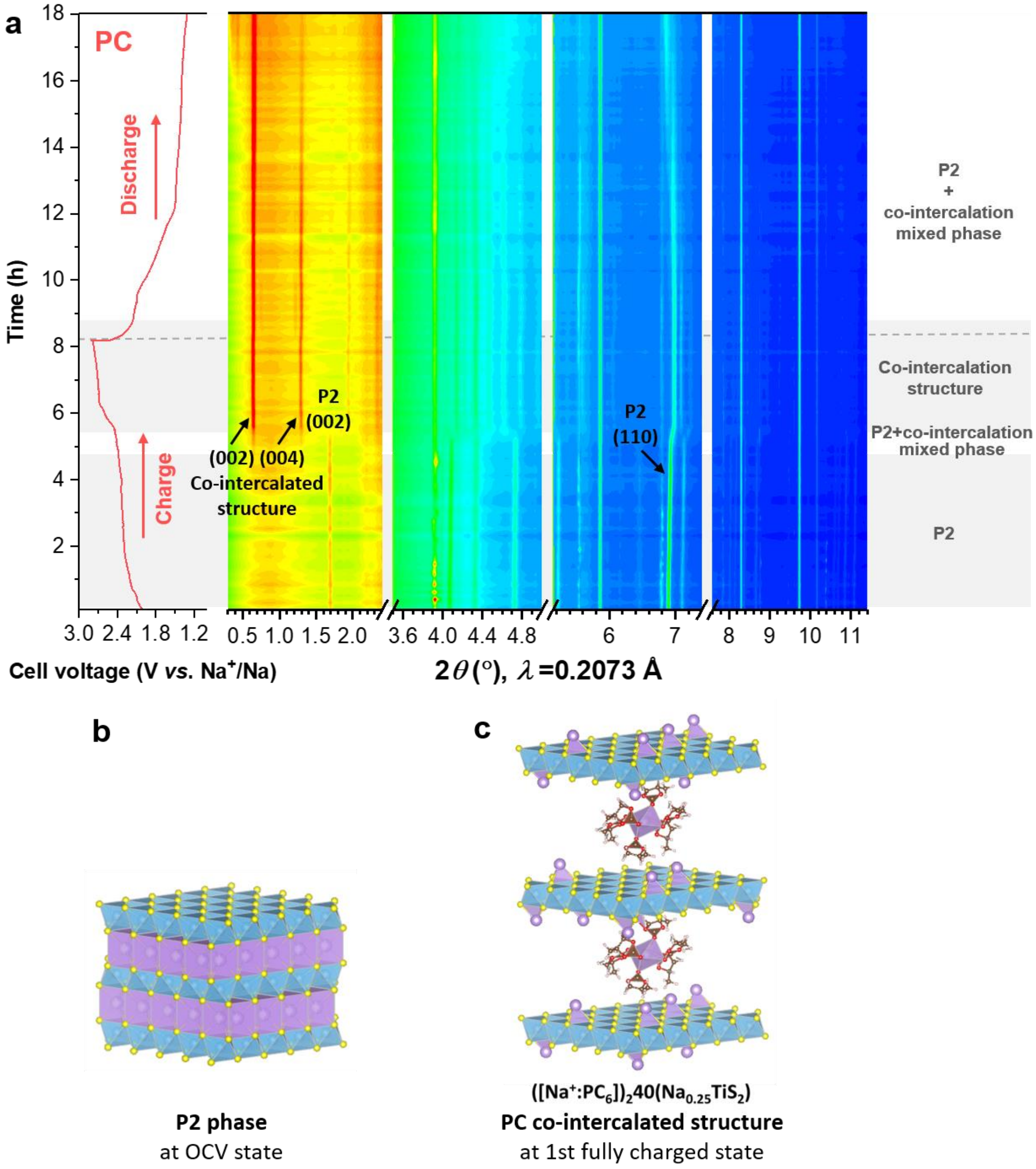

Supplementary Fig. 12 (a) *Operando* XRD of P2-Na_xTiS_2 in 0.5 M $NaPF_6$ in PC electrolyte for the first cycle at 0.125C. A schematic illustration of the P2 phase (b) and PC co-intercalated structure (c). The Na ions are indicated in purple. These can be coordinated to the transition metal sulfur (TMS)-layer or coordinated by solvent molecules. After the cell desodiates to 2.43 V *vs*. Na^+/Na, the P2 phase disappears and a new phase emerges with the characteristic peaks at $2\theta = 0.65°$ and 1.30° 2θ. These low-angle peaks indicate a significant interlayer expansion, which is typically observed when solvent co-intercalation occurs. Co-intercalation of PC molecules into Na_xTiS_2 has not been reported before. At the same time, co-intercalation of PC along with Li^+ is a well-known and undesired process for graphite electrodes, though it occurs at much lower potentials of about 1 V *vs*. Li^+/Li.

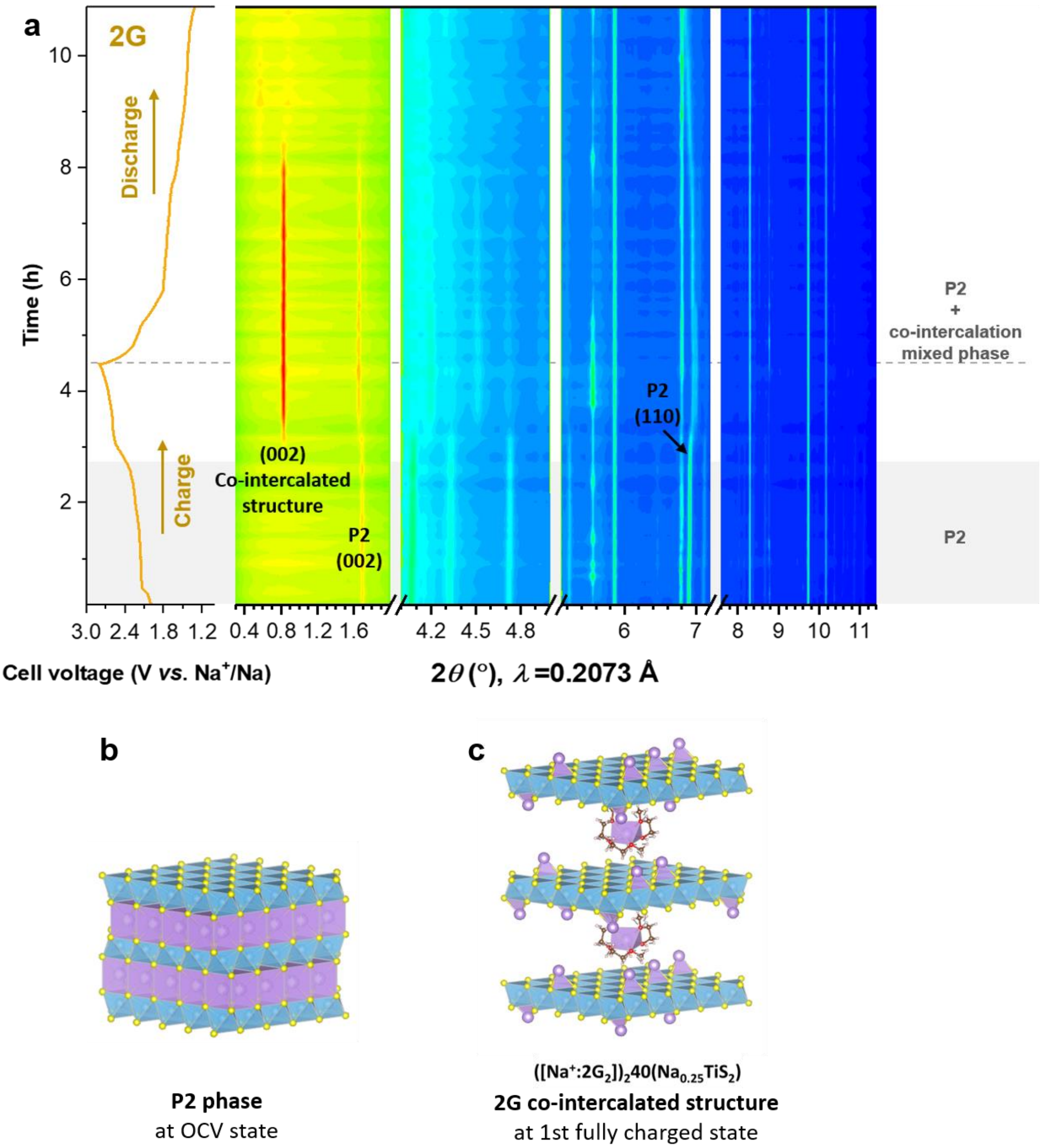

Supplementary Fig. 13 (a) *Operando* XRD of P2-Na_xTiS_2 in 1.0 M $NaPF_6$ in 2G electrolyte for the first cycle at 0.125C. A schematic illustration of the P2 phase (b) and 2G co-intercalated structure (c). The Na ions are indicated in purple. These can be coordinated to the transition metal sulfur (TMS)-layer or coordinated by solvent molecules. For the 2G electrolyte, the phase behavior was also found to differ from the EC/DMC electrolyte. After charging above 2.47 V *vs*. Na^+/Na, an additional peak at $2\theta = 0.83°$ appeared, similar to what has been found for the PC electrolyte. This again indicates a significant expansion of the layered structure. These peaks are maintained during sodiation until a potential of 1.55 V *vs*. Na^+/Na, at which point they disappear, although low-intensity peaks appear at even lower angles.

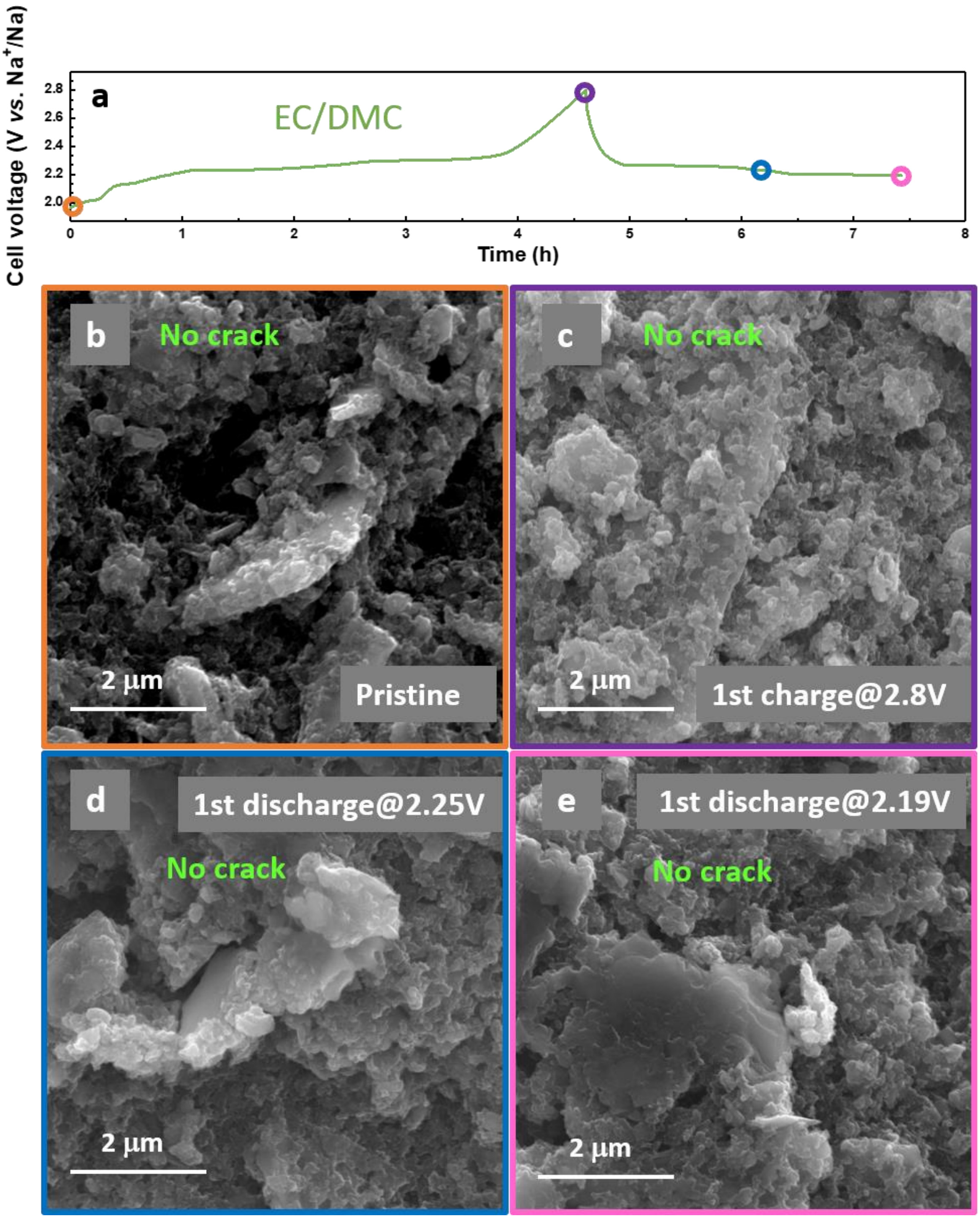

Supplementary Fig. 14 *Ex-situ* SEM of P2-Na_xTiS_2 in 1.0 M $NaPF_6$ in EC/DMC electrolyte at pristine state (b), 1st desodiation at 2.8 V *vs*. Na^+/Na, 1st sodiation at 2.25 V *vs*. Na^+/Na, and 1st sodiation at 2.19 V *vs*. Na^+/Na. No noticeable changes in the morphology of P2-Na_xTiS_2 during the desodiation and sodiation processes.

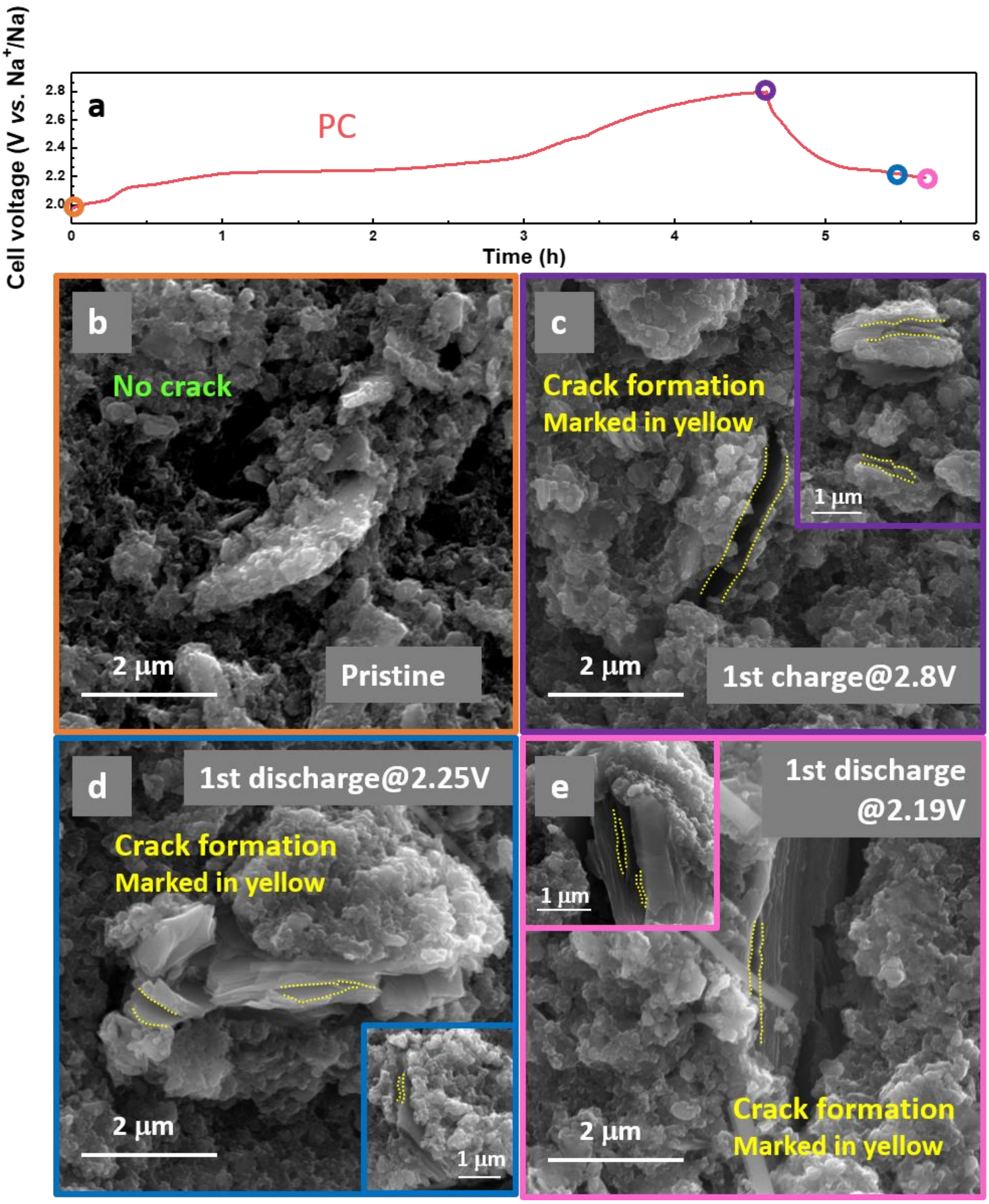

Supplementary Fig. 15 *Ex-situ* SEM of P2-Na_xTiS_2 in 0.5 M $NaPF_6$ in PC electrolyte at pristine state (b), 1st desodiation at 2.8 V *vs*. Na^+/Na, 1st sodiation at 2.25 V *vs*. Na^+/Na, and 1st sodiation at 2.19 V *vs*. Na^+/Na. It can be observed that compared to the compound at pristine state, the electrode materials at the fully desodiated state and different sodiation states at 2.25 V and 2.19 V *vs*. Na^+/Na exhibit noticeable cracks, reflecting the influence of PC co-intercalation on the Na_xTiS_2 structure. The degree of cracking is less compared to graphite,[13] but the lattice expansion is much greater in this case.

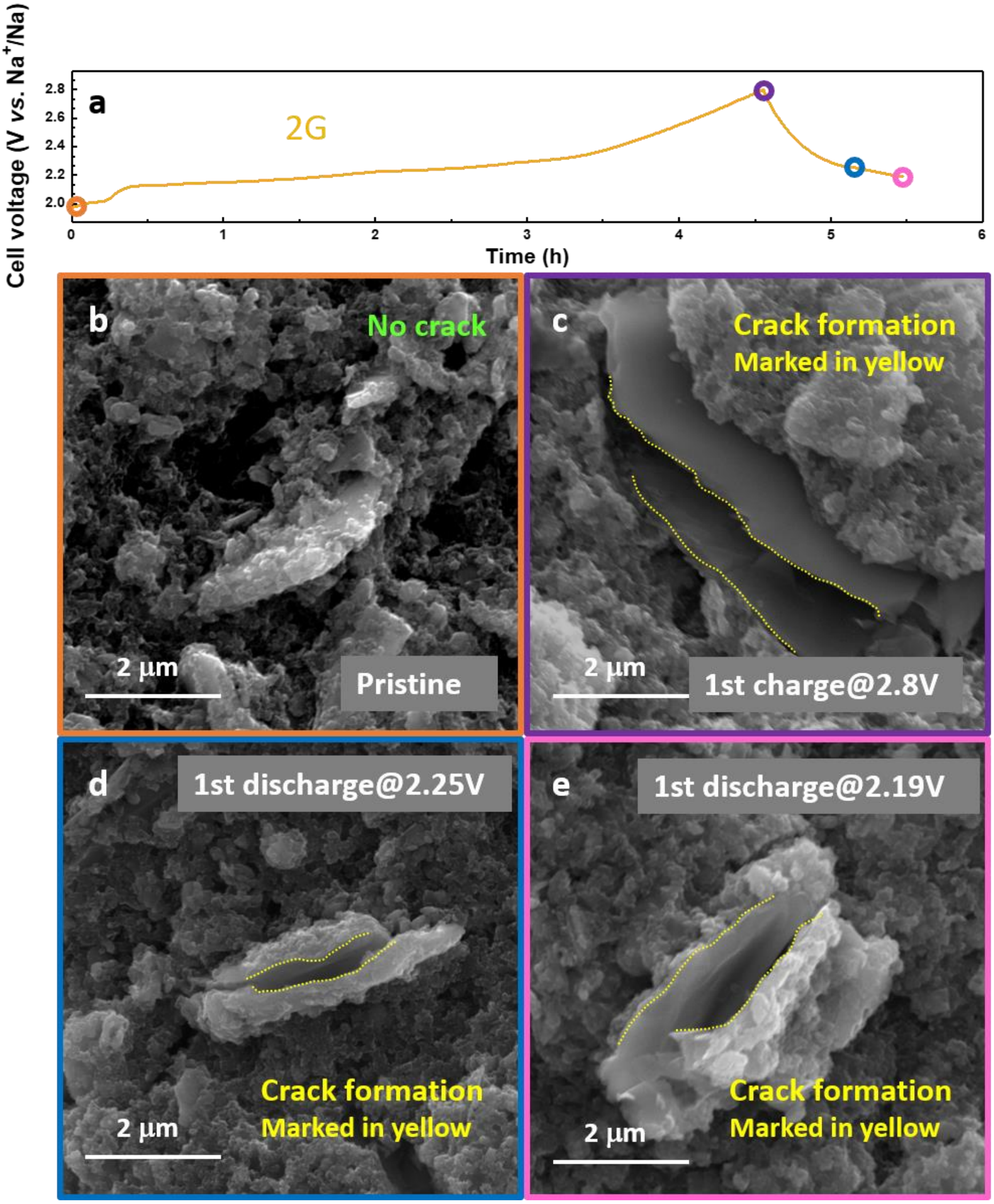

Supplementary Fig. 16 *Ex-situ* SEM of P2-Na_xTiS_2 in 1.0 M $NaPF_6$ in 2G electrolyte at pristine state (b), 1st desodiation at 2.8 V *vs*. Na^+/Na, 1st sodiation at 2.25 V *vs*. Na^+/Na, and 1st sodiation at 2.19 V *vs*. Na^+/Na. As expected from the large layer expansion, crack formation can be clearly observed. The degree of cracking is less compared to graphite,[13] but the lattice expansion is much greater in this case.

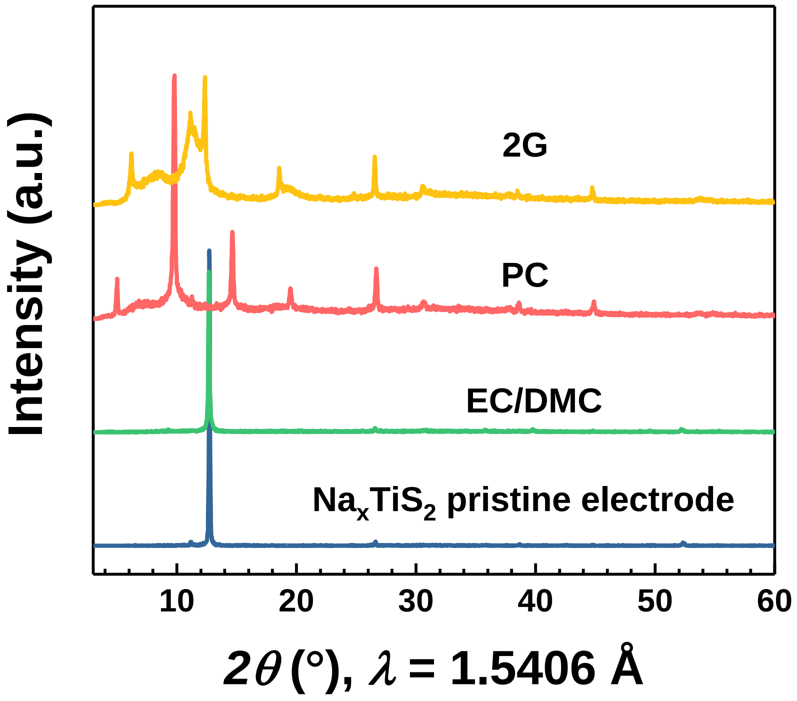

Supplementary Fig. 17 *Ex-situ* XRD of the P2-Na_xTiS_2 electrode after cycling for 20 cycles and then discharging to 2.19 V *vs*. Na^+/Na in three different electrolytes. After discharging to 2.19 V in the EC/DMC-based electrolyte, the main peak of the Na_xTiS_2 electrode at 12.7° corresponding to the P2 phase is still observable, and no signals are detected in the low-angle region. However, in PC and 2G electrolytes, the characteristic co-intercalation peaks at 5° and 6.2° (λ = 0.15406 nm), respectively, can also be observed under the same conditions, consistent with the peaks observed in the *operando* XRD analysis during the first cycle. This indicates that the co-intercalation structure is stable and maintained even after 20 cycles.

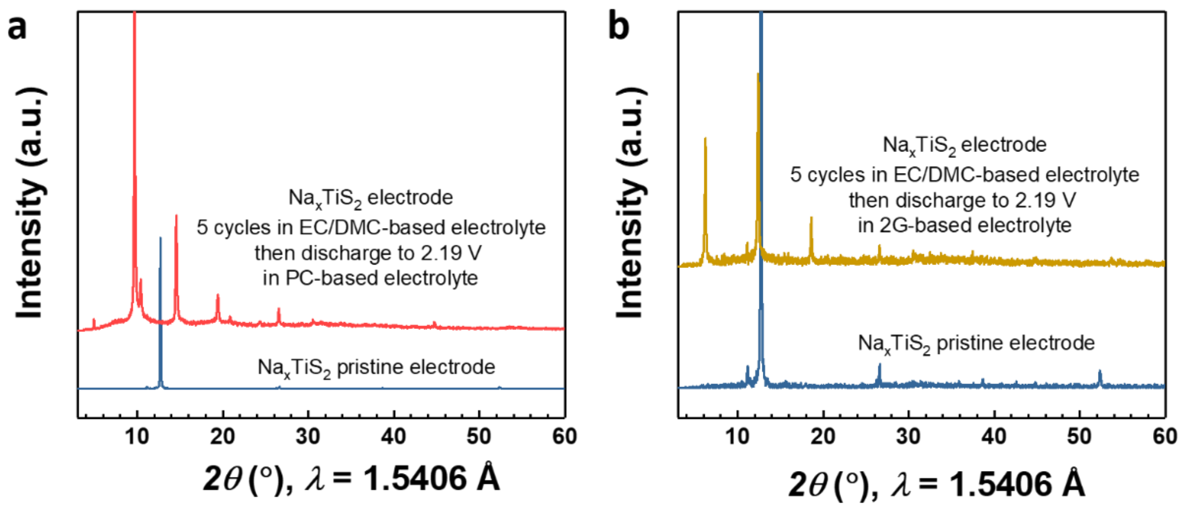

Supplementary Fig. 18 *Ex-situ* XRD of the P2-Na_xTiS_2 electrode cycled after 5 cycles in EC/DMC electrolyte and then sodiated to 2.19 V *vs*. Na^+/Na in PC (a) and 2G (b). P2-Na_xTiS_2 electrodes cycled in the EC/DMC-based electrolyte for 5 cycles were disassembled and then reassembled in both PC- and 2G-based electrolytes to cycle under the same measuring conditions. These measurements confirm that co-intercalation takes place in these cases as well, which provides clear evidence that the process only occurs for specific solvents (PC or 2G).

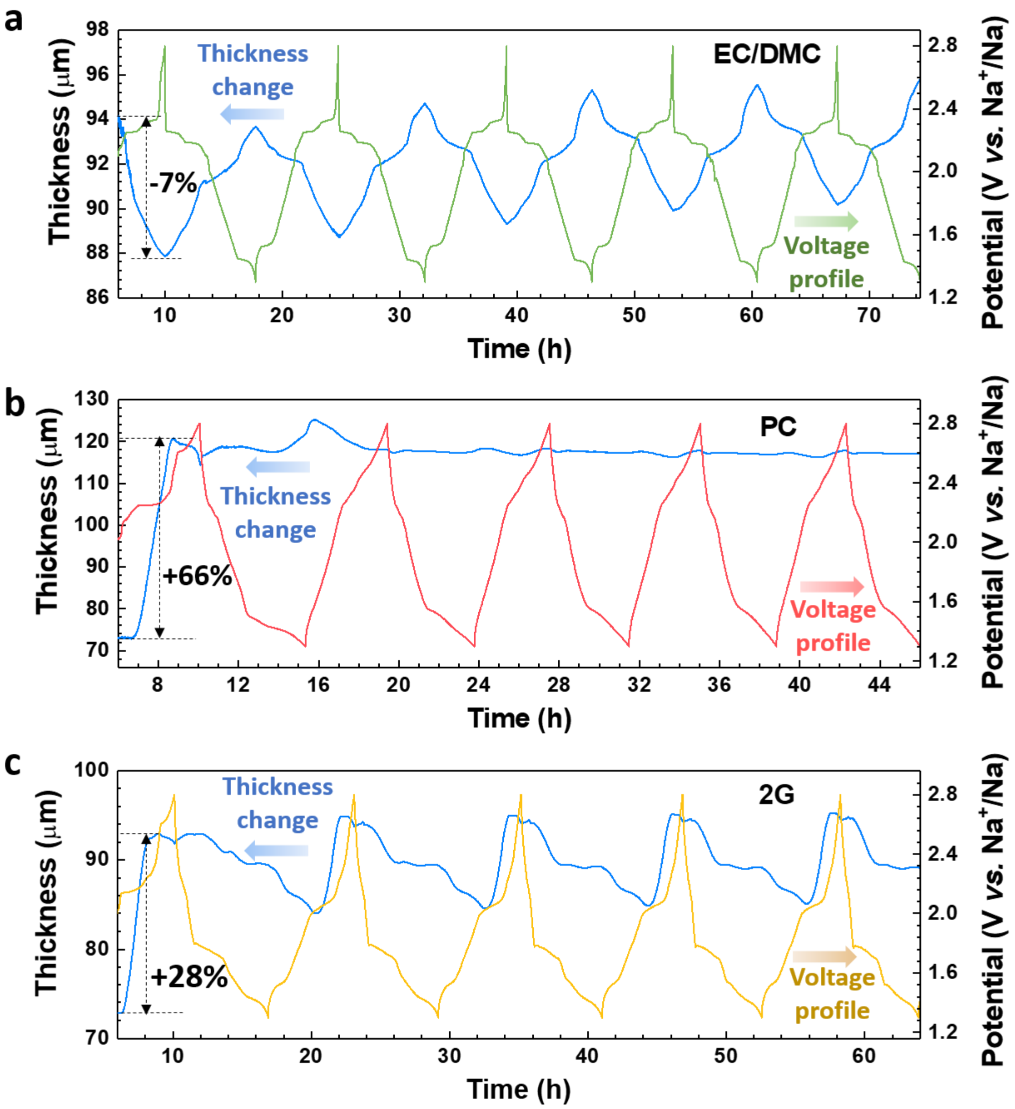

Supplementary Fig. 19 *Operando* electrochemical dilatometry results for P2-Na_xTiS_2 in different electrolytes for the first five cycles (3-electrode setup) at 0.1C. The blue curves show the thickness of the P2-Na_xTiS_2 electrode during cycling. For the PC and 2G-based electrolytes, there is only a slight change in the electrode thickness from the second cycle and the thickness of the electrode does not decrease back to the initial value after the expansion occurring in the first desodiation process, indicating that the structure remains expanded.

Solid-state NMR experiments were conducted as a direct probe of the local environment of the solvent molecules to evaluate their presence in the CAMs layers. ^{23}Na and ^{13}C NMR were measured for P2-Na_xTiS_2 at different states of charge (SoC) using 2G as solvent: pristine state, first desodiation at 2.8 V *vs*. Na^+/Na, first sodiation at 1.75 V *vs*. Na^+/Na, and first sodiation at 1.3 V *vs*. Na^+/Na.

The ^{23}Na NMR spectrum (Supplementary Fig. 20, black trace) of the pristine P2-Na_xTiS_2 material reveals two environments – a diamagnetic and a paramagnetic one. The signals in the diamagnetic region close to 0 ppm are assigned to inorganic side-phases, which are expected to be impurities from the synthesis such as inorganic sulfides. According to DFT calculations of the S-Na_2S system,[14] the most stable sodium polysulfide in this system should be Na_2S_4, as indicated by its presence at the bottom of the computed convex hull. Na_2S_4 displays two distinct sodium sites corresponding to a sharp resonance at +9.5 ppm and a broader peak at –0.4 ppm in ^{23}Na NMR spectra, exhibiting a quadrupolar coupling constant around 1.0 and 1.7 MHz, respectively.[15] This pattern matches the one observed for the diamagnetic range in the ^{23}Na MAS NMR spectra of pristine P2-Na_xTiS_2, hence supporting the assignment of these resonances to Na_2S_4 as a side phase in the sample. Na_2S_4 is a very soluble compound and is likely dissolved into the electrolyte upon preparation of the cell,[16] which would explain the disappearance of these two signals from the diamagnetic range of the spectra when comparing the pristine P2-Na_xTiS_2 sample to those measured *ex-situ* after disassembling the electrochemical cells.

An additional signal is present in the paramagnetic region with an isotropic shift at +91 ppm (see Supplementary Fig. 21 for deconvolution data of the paramagnetic site). This unusual position could be related to paramagnetic interactions with electronic spins from Ti^{3+} centers in the P2-Na_xTiS_2. The amount of sodium in the pristine P2-Na_xTiS_2 is approximately $x\sim0.65$ (according to the voltage profiles of the P2-Na_xTiS_2), which allows for both Ti^{3+} and Ti^{4+} centres to be present. Upon closer inspection, this peak shows a characteristic shape for a 2nd order quadrupolar interaction (C_Q 1.6 MHz, $\eta\sim0$), which matches previous literature on sodium intercalated in TiS_2 dichalcogenides.[17] The axially symmetric environment denoted by the asymmetry parameter $\eta\sim0$ for ^{23}Na in the pristine material can be explained by the small interlayer space, where sodium sits in the mid-point between the TiS_2 layers.

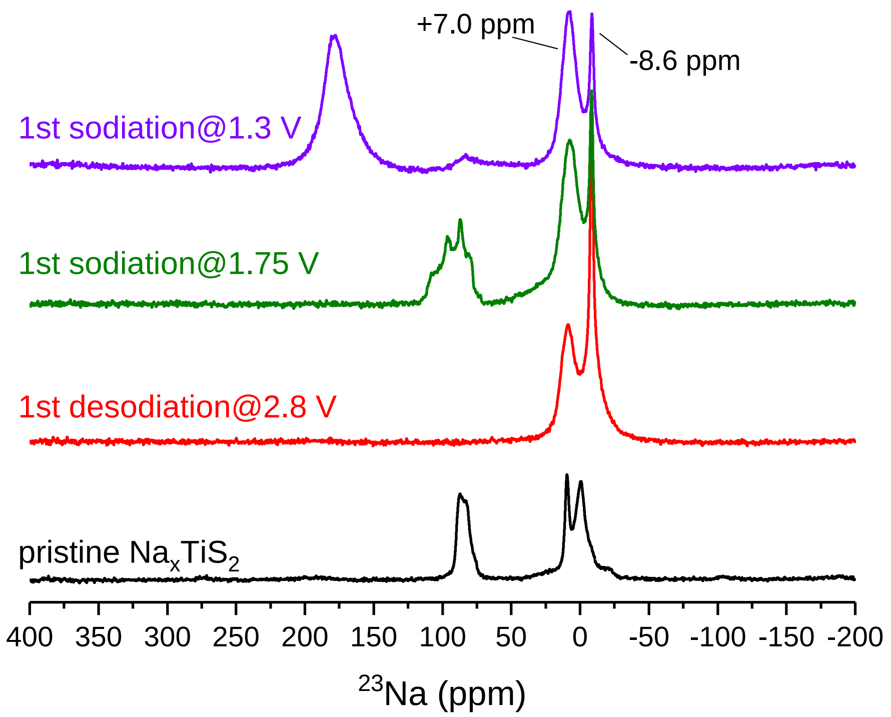

Supplementary Fig. 20 ^{23}Na MAS NMR spectra (20 kHz) of P2-Na_xTiS_2 at different SoC in 1 M $NaPF_6$ in 2G electrolyte.

During charging, the sample is desodiated resulting in P2-Na_xTiS_2 with x~0.1 at 2.8 V (Supplementary Fig. 20, red trace). Due to charge compensation, very few Ti^{3+} centers remain. This is reflected in the ^{23}Na NMR spectrum, where almost no paramagnetic contribution is observed. During desodiation, the P2-Na_xTiS_2 structure is expanded along the *c* axis and solvent/electrolyte is entering the structure. The sharp signal at –8.6 ppm in the diamagnetic region is assigned to bulk liquid electrolyte.[18] However, the assignment of the broad resonance at +7.0 ppm is more daring. The featureless line shape indicates disorder or slowed down dynamics and its position in the diamagnetic range of ^{23}Na chemical shift reflects the absence of pronounced paramagnetic interactions. If the solvent molecules can enter the structure and solvated Na^+ ions can be formed, the organic portion could "shield" paramagnetic interactions from the Ti^{3+} centres or set the distance from Na^+ ions to the layers so large as to considerably decrease the effect from this interaction. The broader peak at +7.0 ppm is therefore tentatively assigned to solvated Na^+ ions in the interlayer space of the expanded P2-Na_xTiS_2.

During the following sodiation at 1.75 V *vs*. Na^+/Na (Supplementary Fig. 20, green trace) both the bulk electrolyte outside the layered structure (–8.6 ppm) and solvated Na^+ ions inside the layered structure (+7.0 ppm) are present. The signal area (integral) of the solvated Na^+ ions (in the layered structure) is larger for this SoC than at 2.8 V *vs*. Na^+/Na, which indicates an increase in the amount of the solvated Na^+ ions in the interlayer space upon sodiation. This is in line with the increasing Na content and with the results from *operando* ECD, where the electrode is more expanded at 1.75 V *vs*. Na^+/Na (first sodiation process) compared to at 2.8 V *vs*. Na^+/Na (first desodiation process). In addition to the diamagnetic peaks, a signal re-emerges in the paramagnetic range at ~100 ppm with a similar magnitude as for the pristine sample, which is further assigned as bare Na^+ in the interlayer space. However, its peak shape indicates a change

in the electric field gradient (EFG) tensor due to a different local environment than in the pristine sample. The larger C_Q (2.8 MHz) and η ~ 0.6 point to a non-axial symmetric environment, with a larger distortion of the local environment. Such an environment could be conceived due to the absence of 2G ligands resulting in "naked" Na^+ displaced towards one of the expanded TiS_2 layers. The larger interlayer space should result in an EFG tensor dominated by anions from only one layer, which may explain the larger distortion of this environment in comparison to the pristine sample, and similar results can also be observed from operando XRD that the intensity of the characteristic peaks of the reappeared P2 phase are not as strong as the pristine P2 phase (see Fig. 2b in the main text). The similar magnitude of the paramagnetic shift between this SoC (1.75V *vs*. Na^+/Na, isotropic shift = +111 ppm) and the pristine sample (isotropic shift = +91 ppm) indicates a similar amount of Ti^{3+} centres and therefore a similar bulk magnetic susceptibility for the TiS_2 layers due to a comparable number of unpaired $3d^1$ electrons.

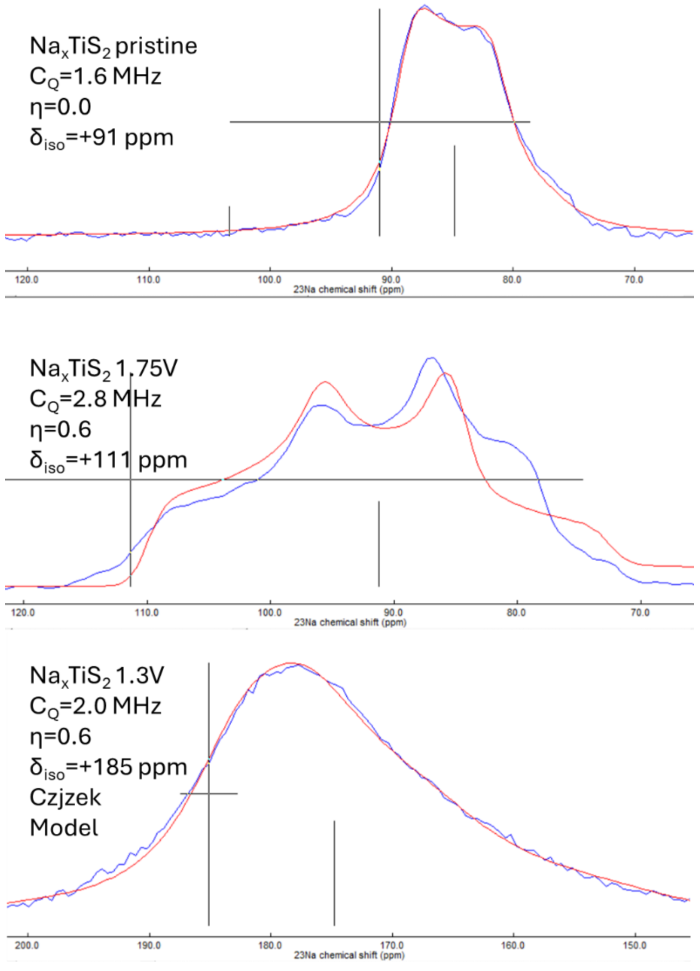

Supplementary Fig. 21 Deconvolution data of the paramagnetic site for the ^{23}Na NMR data. Spectra fitted using the software dmfit.[19]

In the fully sodiated state ($Na_{x\sim1.0}TiS_2$, 1.3 V *vs*. Na^+/Na, blue trace) the peaks in the diamagnetic region are mainly unchanged, indicating the presence of bulk electrolyte (outside the layered structure) as well as solvated Na^+ ions in the layered structure. Interestingly, the ^{23}Na NMR peak (+7.0 ppm, 1.5 kHz fwhm) assigned to the solvated Na^+ in the interlayer space is similar

in magnitude as for the 1.75 V sample (n.b. taking into account the semi-quantitative nature of the measurement, which had the same amount of active cathode material in the NMR rotor in both cases). This indicates that the solvated Na^+ ions in the layered structure reach some kind of "saturation” at a given point in the sodiation curve and more bare Na^+ ions are entering the expanded structure during the sodiation between 1.75 V and 1.3 V *vs*. Na^+/Na. For the latter, the paramagnetic signal (isotropic shift +185 ppm) is much more strongly shifted to higher shift values than in the previous samples. This can be explained by the larger concentration of Ti^{3+} centres in the sodiated state leading to a greater paramagnetic contribution to the signal. Furthermore, the relative intensity of this signal is significantly larger, which goes in line with the higher Na content for this SoC. The signal at +185 ppm is quite broadened and no clear quadrupolar features are discerned. This may be due to a less ordered local structure, in which case the line shape can be appropriately fitted with the Czjzek model with an average value of C_Q=2.0 MHz. Nevertheless, a larger paramagnetic interaction leading to either shorter T_2 values (and further homogeneous broadening the line) or effects related to bulk magnetic susceptibility (and hence inhomogeneous broadening of the line) cannot be discarded. A peak at +100 ppm is still visible, meaning that sodiation is not achieved to completion and some reminiscence of particles (or particle volumes) with intermediate SoC.

To further investigate the co-intercalation of 2G solvent and Na^+, ^{13}C NMR data at different SoC were collected. Overall, the observations from the *ex-situ* ^{13}C MAS NMR spectra indicate three different environments for 2G in the cathode samples, supporting the hypothesis of solvated Na^+ inside the P2-Na_xTiS_2 interlayer space, next to free 2G molecules (also inside the layered structure), as well as bulk electrolyte outside the cathode material.

Supplementary Fig. 22 shows the ^{13}C solution-state NMR spectrum (without ^1H decoupling) of 1M $NaPF_6$ electrolyte in 2G, where the quartet at 58.0 ppm is assigned to the CH_3 groups ($^1J_{CH}$=141 Hz), the triplet at 69.9 ppm ($^1J_{CH}$=141 Hz) to the CH_2 group closest to the central O heteroatom and an additional triplet at 71.6 ppm ($^1J_{CH}$=141 Hz) assigned to the remaining CH_2 group of the bis(2-methoxyethyl) ether $(CH_3OCH_2CH_2)_2O$ molecule. These ^{13}C NMR resonances also display finer structure that indicates scalar J_{CH} coupling further than one bond length, in the range of a few Hz, which albeit their observation in solution do not play a role in solid-state NMR measurements. Note that this spectrum was recorded at a B_0 field of 11 T. At the lower field of 9.4 T, the chemical shift separation between both CH_2 moeities (168 Hz) would be very close to the J coupling of ~140 Hz, resulting in an intricate multiplet pattern, which may complicate, but not completely hinder, the distinction of both CH_2 groups in the ^{13}C solid state NMR spectra.

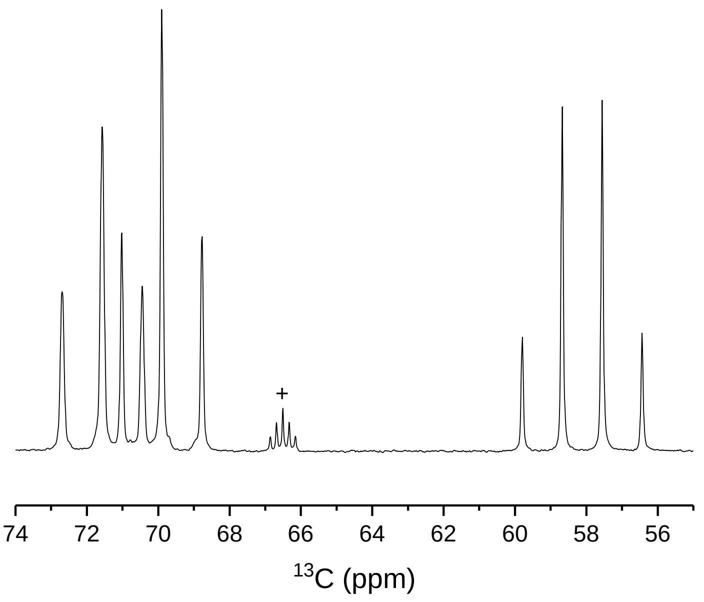

Supplementary Fig. 22 ^{13}C solution-state NMR spectrum of pure 1 M $NaPF_6$ in 2G electrolyte. + denotes d_6-THF used for locking.

In addition to that, there is enough resolution in the solid-state to discern between CH_2 and CH_3 groups. This trait is explored in the evaluation of the P2-Na_xTiS_2 samples, in order to ascertain the location of 2G molecules in the cathode composites as shown in Supplementary Fig. 23 and Supplementary Table 5.

Interestingly, at 2.8 V *vs*. Na^+/Na (see Supplementary Fig. 24 and Supplementary Table 6 for deconvolution data), signals from both CH_3 and CH_2 moieties split up each into three sub-sets of peaks. The first set of peaks (orange fit trace in Supplementary Fig. 24) exhibits very narrow lines (fwhm ~ 20 Hz) with a similar chemical shift as in the solution-state NMR (CH_3 +59.2 ppm, CH_2 +70.8 ppm; +72.5 ppm) and are hence attributed to the free 2G molecules in bulk electrolyte (i.e. outside the cathode material). The second set of peaks (green fit trace) is shifted +4.9 ppm down-field away from the chemical shift values of the narrow set of peaks, with the additional CH_3 signal displaying a clear quartet centred at 64.1 ppm and the double triplet pattern for both CH_2 groups being discerned with a chemical shift of +75.7 ppm and +77.1 ppm, respectively. The direction of this shift matches the one observed in ^{23}Na NMR due to paramagnetic (pseudo-contact) interaction with Ti^{3+} centres from the TiS_2 layers, hence supporting the assignment of the latter set of ^{13}C resonances to solvated Na^+ in the interlayer space. The smaller magnitude of the paramagnetic shift can be understood due to the larger distance to the inorganic layer for 2G molecules when compared to bare Na^+ directly coordinated to S^{2-}.

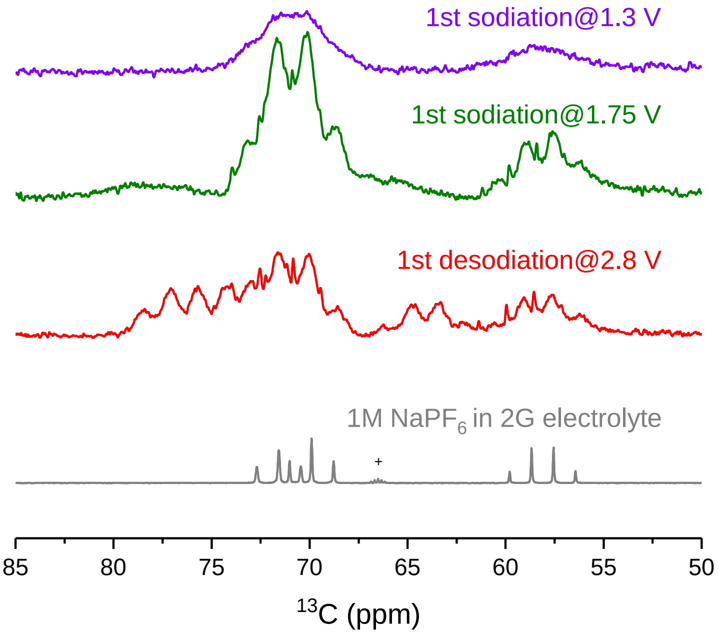

Supplementary Fig. 23 ^{13}C MAS NMR spectra of P2-Na_xTiS_2 at different SoC in 1 M $NaPF_6$ in 2G electrolyte. (Solution-state spectrum of pure 1 M $NaPF_6$ in 2G electrolyte shown in grey. (+) is d6-THF used for locking.)

A third set of signals (light blue) is broader and shifted by about −1.0 ppm from the narrow peaks (i.e. upfield shift at CH_3 +58.4 ppm, CH_2 +70.0 ppm; +71.6 ppm). Their width (fwhm ~80 Hz) and relative position to the set of peaks assigned to bulk electrolyte strongly indicate that this set of signals corresponds to 2G molecules located within the interlayer space. The fact that their signals are considerably broader (fwhm ~80 Hz) than the bulk electrolyte in the sample could either reflect their reduced mobility, which could be ascribed to the constrained environment of the interlayer space, or shorter spin-spin relaxation times T_2 due to paramagnetic interactions with the Ti^{3+} bearing layers. Leifer et al. observe a similar behavior for 2G co-intercalated Na^+ in graphite, where the solvent molecules weakly bound to the surface of graphite are slightly upfield shifted from the bulk electrolyte signal.[18] Hence, this third set of signals is assigned to free 2G solvent molecules in the layered structure.

Three sets of signals are also observed for the sample of the first sodiation at 1.75 V *vs*. Na^+/Na (see Supplementary Fig. 25 and Supplementary Table 7 for deconvolution data). Their evaluation reinforces the hypothesis of paramagnetic interaction determining line position and width for the intercalated 2G complexes and free molecules. In detail, whilst the narrow set of peaks assigned to bulk electrolyte remains in the same position and width as for the 2.8 V *vs*. Na^+/Na sample, the paramagnetic shift towards downfield of the set of peaks of solvated Na^+ in the interlayer space is even more pronounced (see Supplementary Table 5, green, +7 ppm for 1.75 V *vs*. Na^+/Na, instead of +5 ppm for 2.8 V *vs*. Na^+/Na). This trend goes in line with the increased (paramagnetic) bulk magnetic susceptibility of the P2-Na_xTiS_2 layers with increasing x value, due to the larger number of Ti^{3+} centres and the corresponding unpaired $3d^1$ electron spins, whereas both the signals assigned to bulk electrolyte outside the layered structure and

free 2G solvent molecules inside the layered structure remain in the same position. Furthermore, the width of the set of ^{13}C peaks assigned to free 2G solvent molecules in the interlayer space also increases from 80 to 100 Hz, implying a direct relation of these moieties to the SoC of the cathode material, which indicates their interaction with the TiS_2 layers.

Last, these trends are further corroborated by analysis of the sample at SoC 1.3 V *vs*. Na^+/Na (see Supplementary Fig. 26 and Supplementary Table 8 for deconvolution data). For this SoC, signals from the paramagnetic shifted peaks can no longer be observed, and are speculated to be broadened to the background. The remaining signals that can be seen are assigned to the resonances from free 2G solvent molecules in the interlayer space based on their position, and their fwhm increases further to around 160 Hz, an observation that reflects their further interaction with increasingly paramagnetic TiS_2 layers bearing an increasing amount of Ti^{3+} centers.

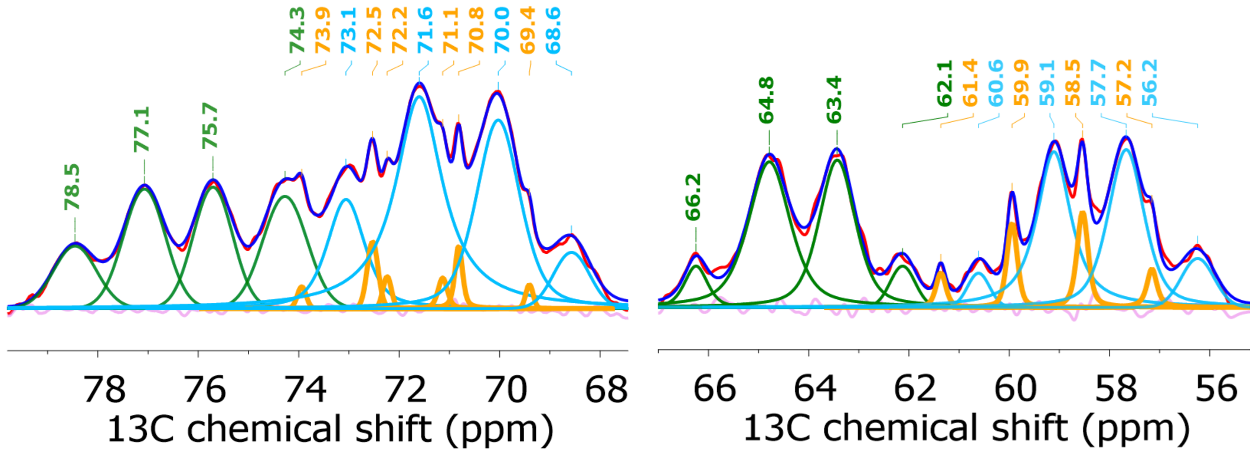

Supplementary Fig. 24 ^{13}C NMR spectrum of the P2-Na_xTiS_2 at the first desodiation at 2.8 V *vs*. Na^+/Na. Red: exp. spectrum, Blue: sum of fitted lines, Light blue: free solvent in the layered structure, Orange: bulk electrolyte outside layered structure, Green: solvated Na^+ in layered structure with evidence for paramagnetic interaction.

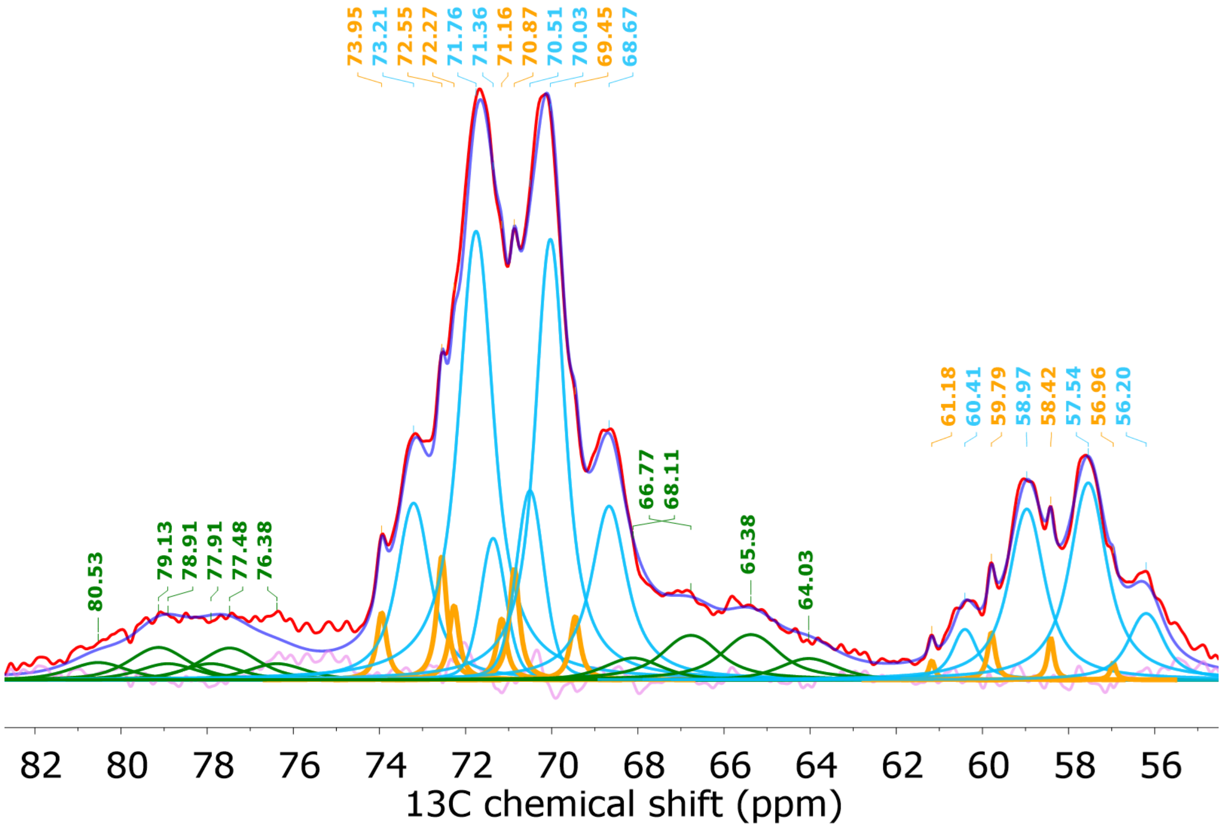

Supplementary Fig. 25 ^{13}C NMR spectrum of the P2-Na_xTiS_2 of the first sodiation at 1.75 V *vs*. Na^+/Na. Red: exp. spectrum, Blue: sum of fitted lines, Light blue: free solvent in the layered structure, Orange: bulk electrolyte outside layered structure, Green: solvated Na^+ in layered structure.

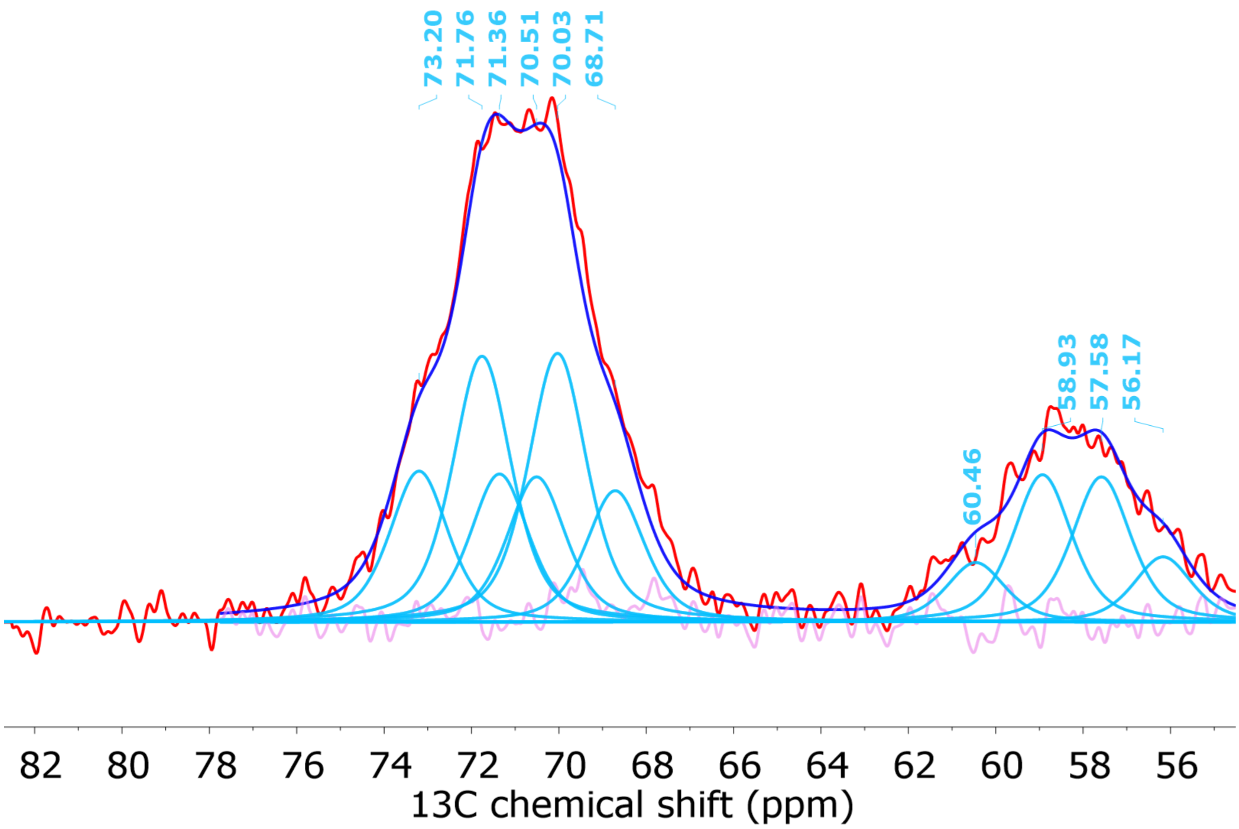

Supplementary Fig. 26 ^{13}C NMR spectrum of the P2-Na_xTiS_2 of the first sodiation at 1.3 V *vs*. Na^+/Na. Red: exp. spectrum, Blue: sum of fitted lines, Light blue: free solvent in the layered structure.

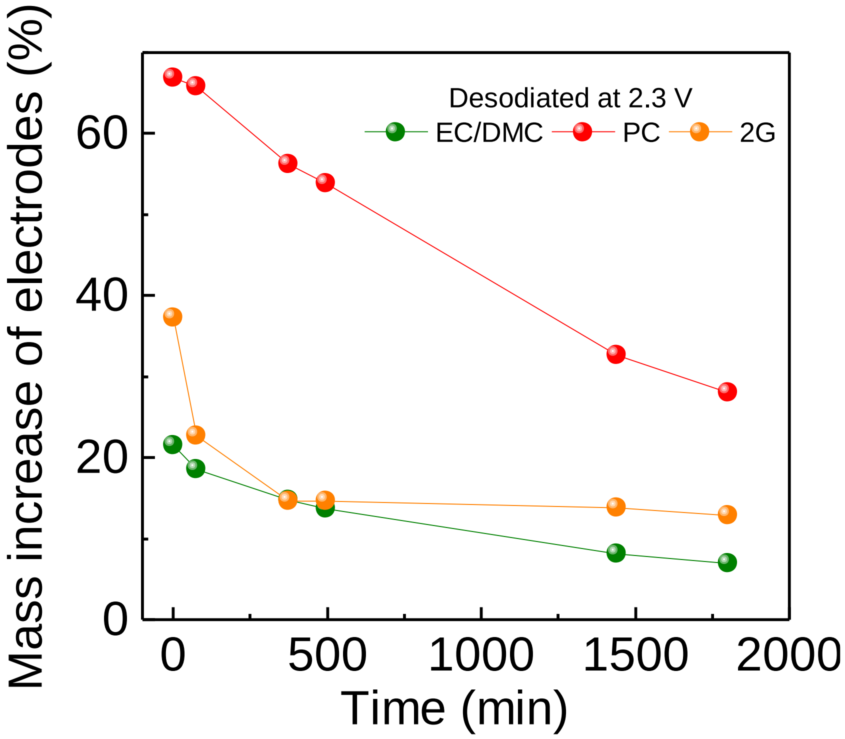

Supplementary Fig. 27 Variation with time of the mass of the P2-Na_xTiS_2 electrodes at 2.3 V vs Na^+/Na in the desodiated state for EC/DMC-, PC- and 2G-based electrolytes. To eliminate the influence of the different vapor pressures of the electrolyte solutions, the masses were measured after disassembling the cells. In the case of 2G, there is an initial removal of solvent electrolyte occurring in the first few hours, which can be attributed to its higher vapor pressure compared to PC and EC/DMC. However, the final mass variation of the PC- or 2G-based electrolyte samples is still higher than that observed for EC/DMC, suggesting that certain solvents might remain inside the electrode. The uncertainty in the weight changes of the electrodes is within ±5.9%.[20]

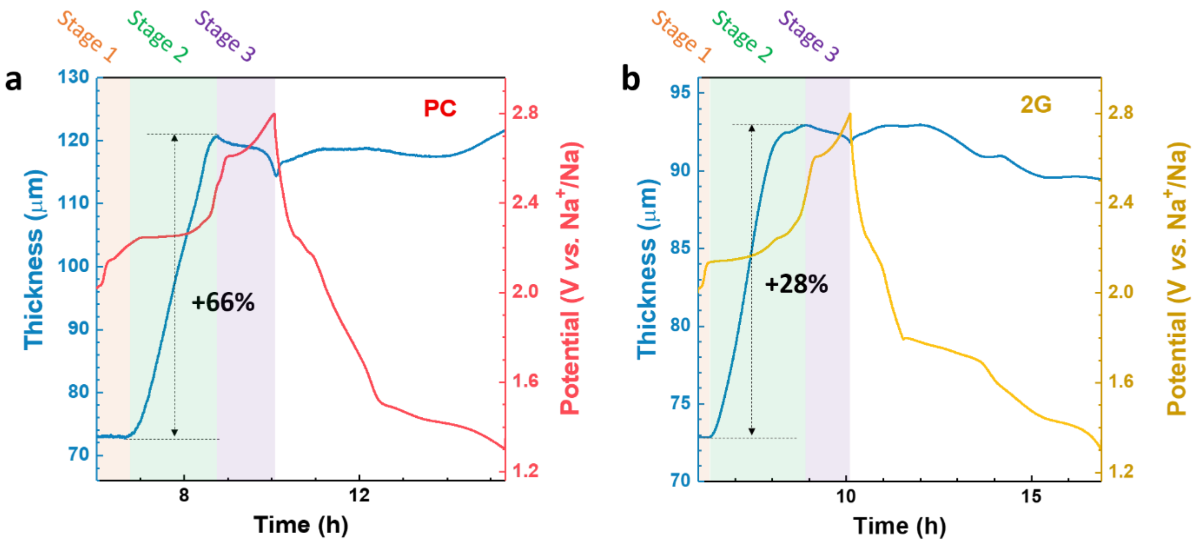

Supplementary Fig. 28 Different stages in the first desodiation and sodiation process of P2-Na_xTiS_2 cycled in 0.5 M $NaPF_6$ in PC (a) and 1 M $NaPF_6$ in 2G.

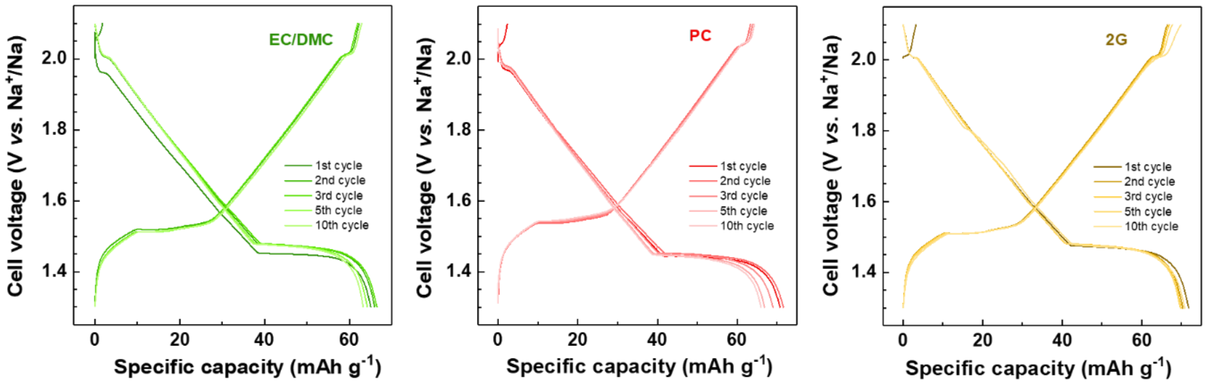

Supplementary Fig. 29 Voltage profiles of the P2-Na_xTiS_2 in different electrolytes in a voltage window of 1.3–2.1 V *vs*. Na^+/Na for the first 10 cycles at 0.1C.

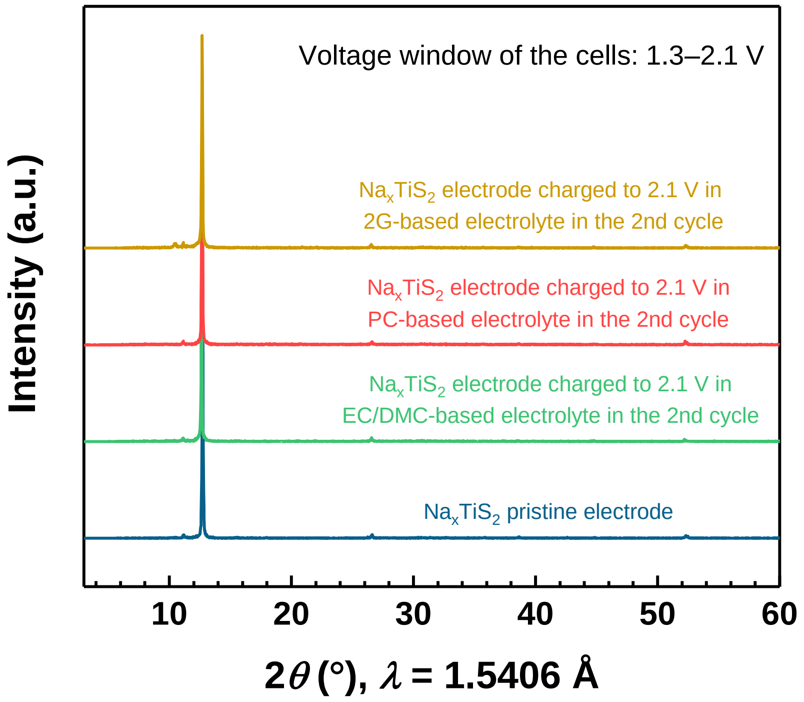

Supplementary Fig. 30 *Ex-situ* XRD of the P2-Na_xTiS_2 electrode desodiated to 2.1 V *vs*. Na^+/Na in three different electrolytes in the second cycle with a voltage window of 1.3–2.1 V. Surprisingly, in this limited voltage range, all the electrodes cycled in the three electrolytes showed identical peaks without any characteristic peaks of the expanded structure arising from co-intercalation.

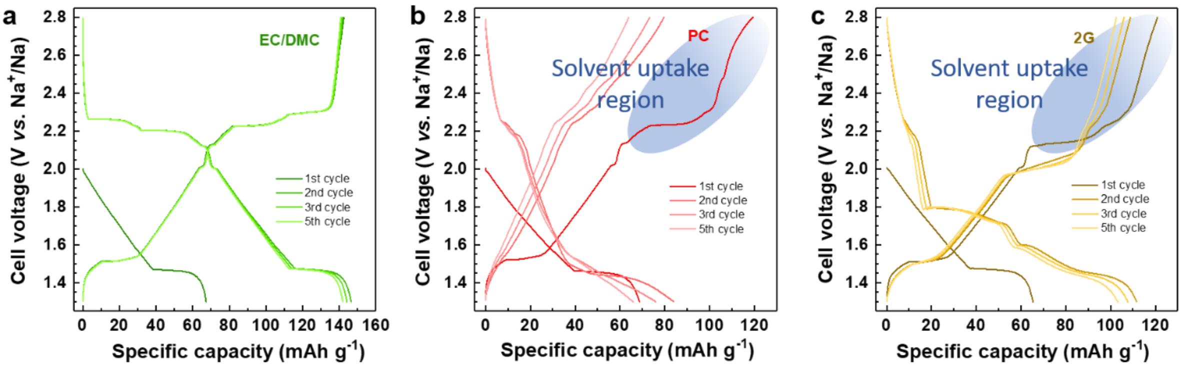

Supplementary Fig. 31 Voltage profiles of the P2-Na_xTiS_2 starting from the sodiation process in different electrolytes in a voltage window of 1.3–2.8 V *vs*. Na^+/Na at 0.1C. The area marked in blue indicates the region of solvent uptake in the first desodiation process. No difference was observed in the three electrolytes before the high-voltage region of the first desodiation process. In the subsequent desodiation and sodiation processes, the solvent uptake started (as marked in the figure), and typical electrochemical behaviors with the expanded structure were observed in both PC- and 2G-based electrolytes. This further indicates that the low sodium content in the P2-Na_xTiS_2 during cycling is a crucial factor for the diffusion of PC or 2G solvent into the layered structure.

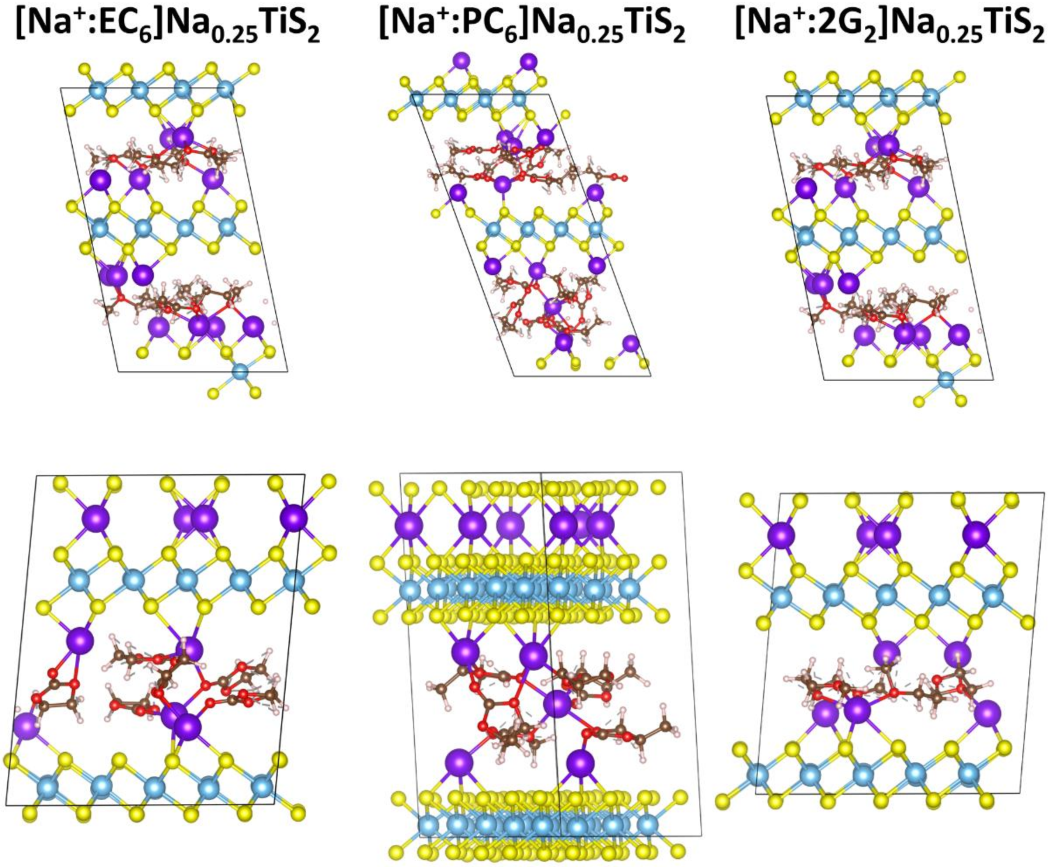

Supplementary Fig. 32 VASP optimized structures where the top row is using a 1 1 1 Γ-centred k-point mesh, using the structures seen in Supplementary Fig. 12c and 13c as starting structures. The bottom row contains only one expanded structure with a solvation shell but using a 4 4 4 Γ-centred k-point mesh. In every case, the optimization algorithm quickly started to decrease the *c* lattice parameter.

To further investigate the structure, sodium ions solvated by 6 EC, 6 PC and 2 2G molecules, respectively, were placed inside a $Na_{0.25}TiS_2$ structure with one layer expanded, and structural optimization was performed, as shown in Supplementary Fig. 32. In all cases, solvents left the solvation shell and start to coordinate to the sodium layer in the $Na_{0.25}TiS_2$, indicating a strong interaction between all solvents and the sodium layer. Some solvents preferred to interact directly with the sodium layer instead of fully solvating an ion. Thus, any solvents entering the structure would likely assist in the desodiation process and solvate sodium as they are about to leave the structure, already inside the bulk material. This would cause the structure to remain partially open, allowing neutral solvent molecules to enter the space previously occupied by the solvated ions, which is also in line with our *operando* XRD results. During the desodiation/sodiation process, there would hence be solvated ions already in the structure, as well as solvent molecules coordinating directly to the sodium layer in the layered structure. As the solvents also show a propensity to coordinate directly to the sodium layer instead of the solvated ions, the structure would always be expanded as long as there is sodium inside, consistent with the XRD and ECD observations. However, during the structural optimization, as the solvents break away from the solvated ion, the structure contracts such that they no longer

match the structures observed in XRD. This shows that a single solvation shell is not enough to cause structures expanded to such a degree as seen in *operando* XRD. Moreover, simply expanding the structure by placing a central solvation shell creates an awkward and unrealistic coordination environment for the sodium bound to the sulfur layer (Supplementary Fig. 12c and 13c). Larger structures with more solvents would be needed to investigate this further. However, due to the number of particles required for these simulations, the computational resources needed are currently unfeasible at this level of theory.

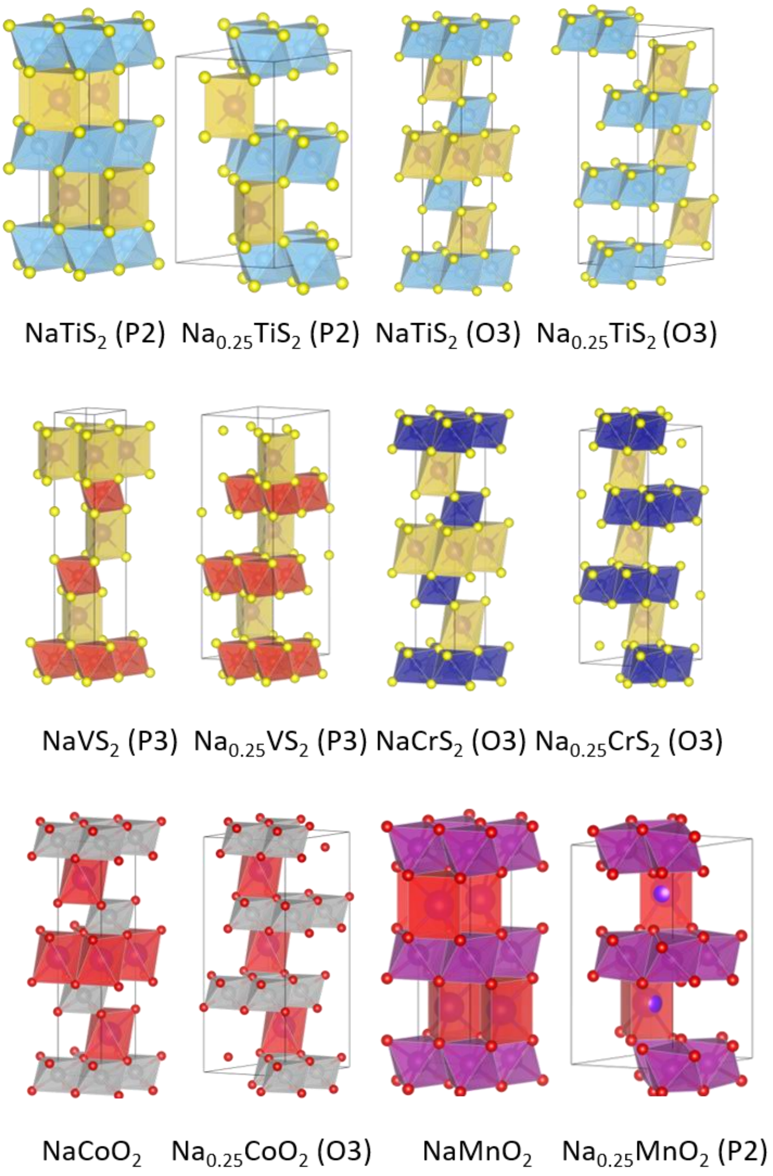

Supplementary Fig. 33 VASP optimized structures which are fully ($NaMS_2/O_2$) and partially sodiated ($Na_{0.25}MS_2/O_2$).

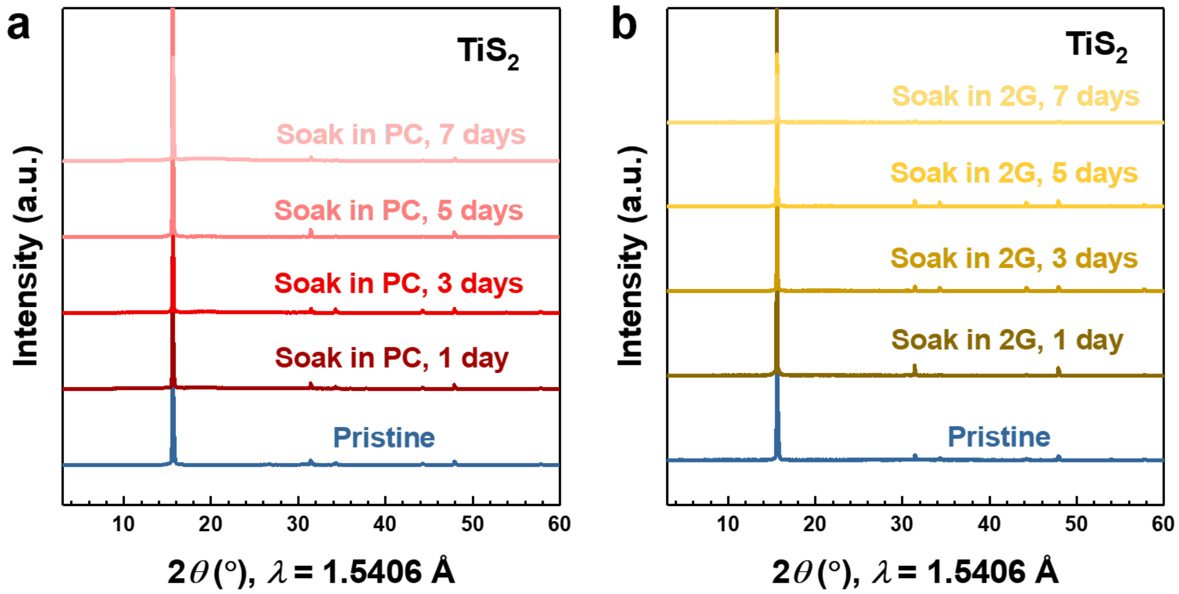

Supplementary Fig. 34 XRD patterns of TiS_2 electrodes soaked in PC or 2G solvents for 1 day, 3 days, 5 days, and 7 days. No expanded structure is observed in both cases after soaking the TiS_2 electrode in PC or 2G solvents for 7 days, indicating no chemical uptake of PC or 2G solvents for TiS_2 electrodes.

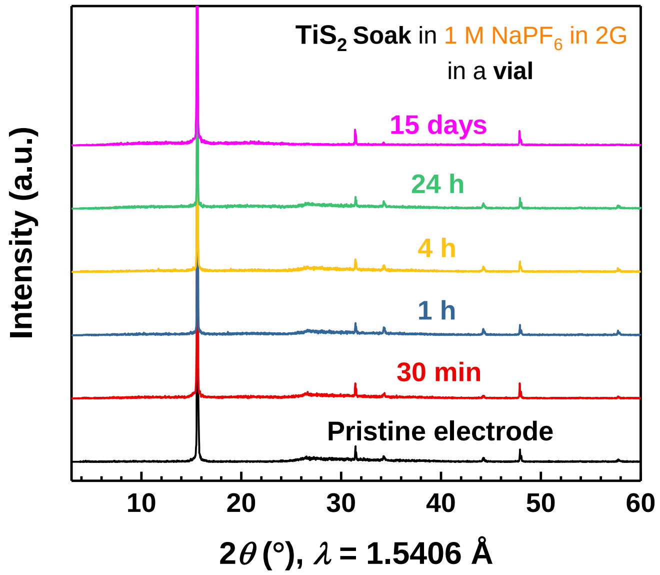

Supplementary Fig. 35 XRD patterns of TiS_2 electrodes soaked in 1.0 M $NaPF_6$ in 2G electrolyte for 30 min, 1 h, 4 h, 24 h, and 15 days. No expanded structure is observed after soaking the TiS_2 electrode in the 2G-based electrolyte even for 15 days, indicating no chemical uptake of 2G solvents for TiS_2 electrodes without reduction current (electrochemical driving force).

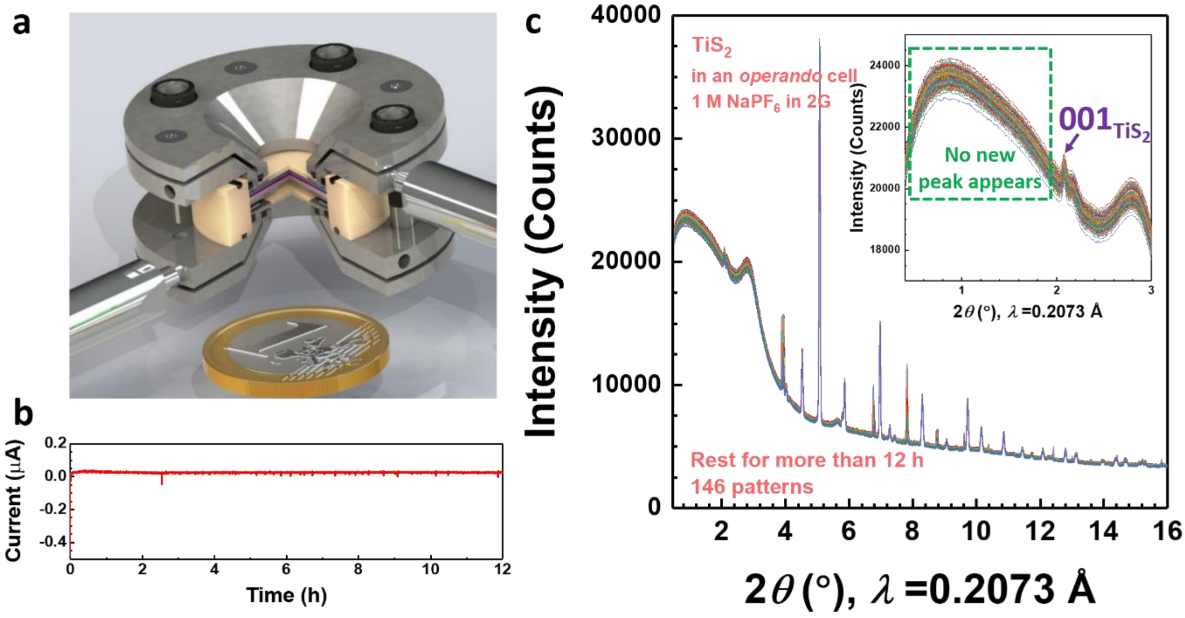

Supplementary Fig. 36 (a) Our in-house designed *operando* cell. (b) Recorded current in the OCV state of TiS_2 in 1.0 M $NaPF_6$ in 2G electrolyte assembled in the in-house designed *operando* cell for 12 h. (c) *Operando* XRD patterns of TiS_2 in 1.0 M $NaPF_6$ in 2G electrolyte assembled in the in-house designed *operando* cell for resting in the OCV state for 12 h.

An *operando* XRD at the P02.1 beamline at DESY was measured, with a prepared *operando* cell by using our in-house designed *operando* cell (Supplementary Fig. 36a), where TiS_2 was used as a working electrode, 1 M $NaPF_6$ in 2G was used as an electrolyte, and Mylar foil was used as a window material. As shown in Supplementary Fig. 36b, there is no leakage current even after resting for 12 h in the OCV state, and the current in the OCV state is near 0 but stay positive (< +0.03 μA) in the whole resting time. As expected, no new peak/phase appears, and the TiS_2 kept the pristine structure after resting for 12 h at OCV (as shown in Supplementary Fig. 36c, where all the patterns during the 12 h resting time were included in the figure).

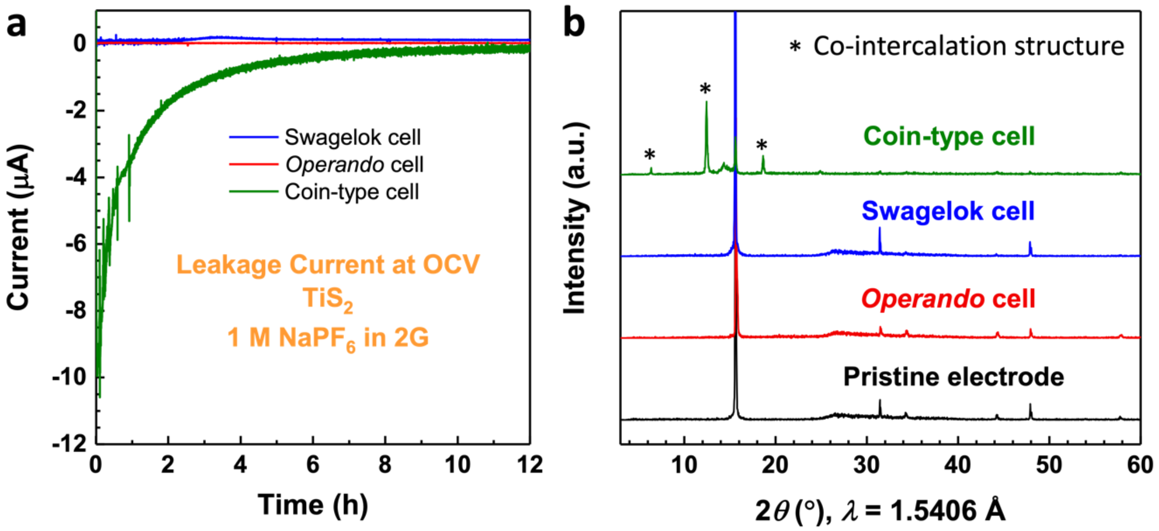

Supplementary Fig. 37 (a) Recorded current in the OCV state of TiS_2 in 1.0 M $NaPF_6$ in 2G electrolyte assembled in three different types of cells. (b) *Ex-situ* XRD patterns of TiS_2 in 1.0 M $NaPF_6$ in 2G electrolyte assembled in three different types of cells for resting in the OCV state.

The leakage current of different cell types using otherwise identical conditions (Na as counter electrode, TiS_2 as working electrode, 1 M $NaPF_6$ in 2G as electrolyte solution) is compared. We surprisingly found that in the OCV state of all the prepared cells, the coin cell showed a huge negative leakage current (–10 μA at the beginning, as shown in Supplementary Fig. 37a), which is already large enough to reduce the TiS_2, i.e. causing its sodiation. The Swagelok-type cell and our in-house designed *operando* cell showed only negligible currents (< +0.1 μA, i.e. 100 times smaller than that in the coin cell and also of opposite sign), indicating there is no leakage current at the OCV state in these two types of cells. *Ex-situ* XRD measurements were then made for the three types of cells. As shown in Supplementary Fig. 37b, the original TiS_2 structure remains unchanged for the Swagelok-type and *operando* cell, evidencing that TiS_2 is stable and does not take up solvent by itself. Only for the coin cell, a co-intercalation phase was found. Combining these results, it becomes clear that the leakage current is the origin for the formation of a co-intercalation phase. In conclusion, we do not find any evidence for any chemical uptake of 2G by TiS_2. Instead, the uptake is very likely caused by a leakage current caused by faulty crimping. This leakage current causes reduction of TiS_2 along with the intercalation of Na^+ and solvents – with the formation of a co-intercalation compound as a result. We reproduced the results several times and the findings are also supported by the *operando* XRD results obtained from the synchrotron radiation beamline as shown in Supplementary Fig. 36c.

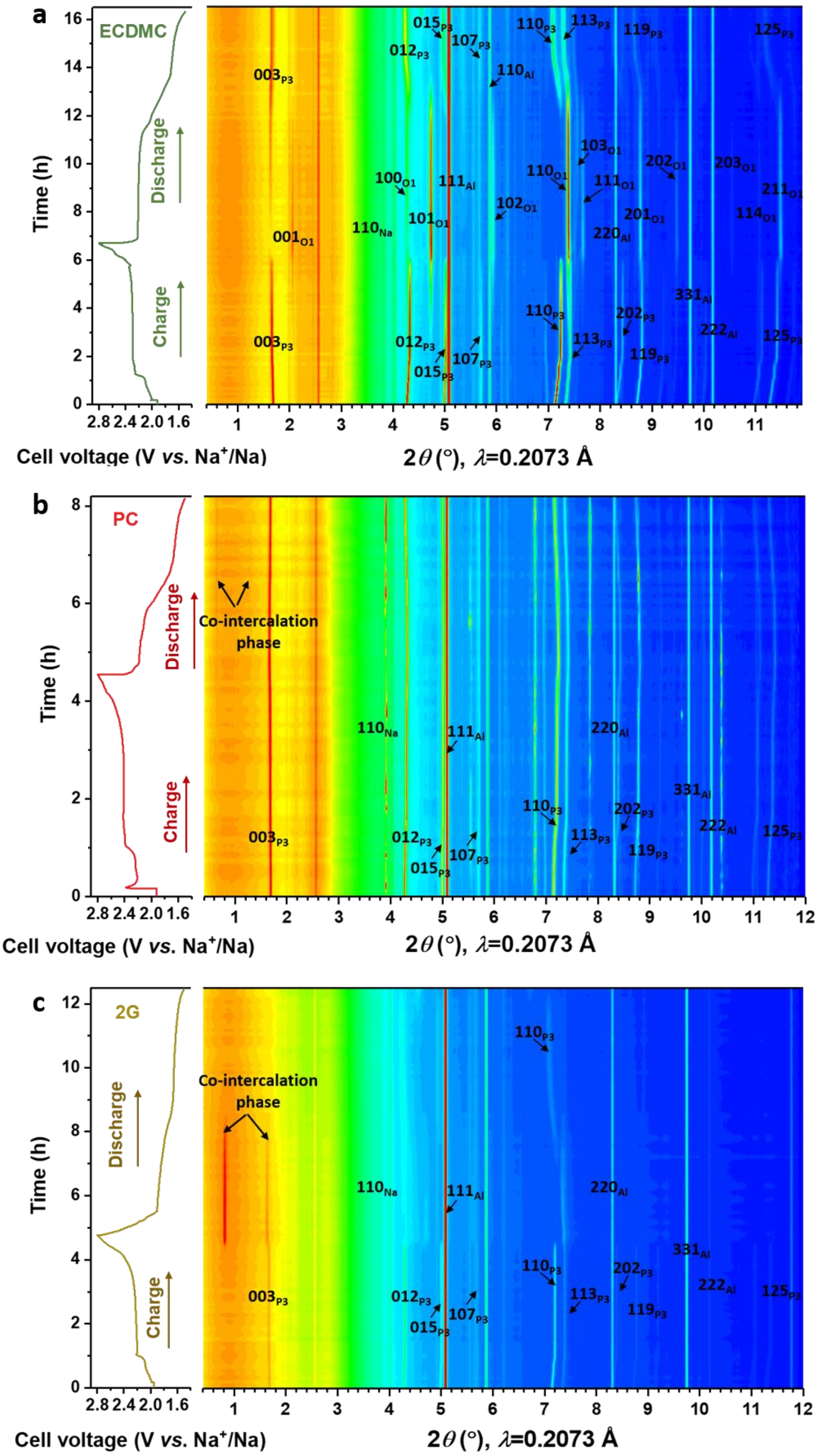

Supplementary Fig. 38 *Operando* XRD of Na_xVS_2 in 1 M $NaPF_6$ in EC/DMC (a), 0.5 M $NaPF_6$ in PC (b), and 1 M $NaPF_6$ in 2G (c) electrolytes for the first cycle at 0.125C. Similar to P2-Na_xTiS_2, only intercalation with a phase transition of P3 to O1 occurs in the EC/DMC-based electrolyte, while co-intercalation occurs in both PC- and 2G-based electrolytes.

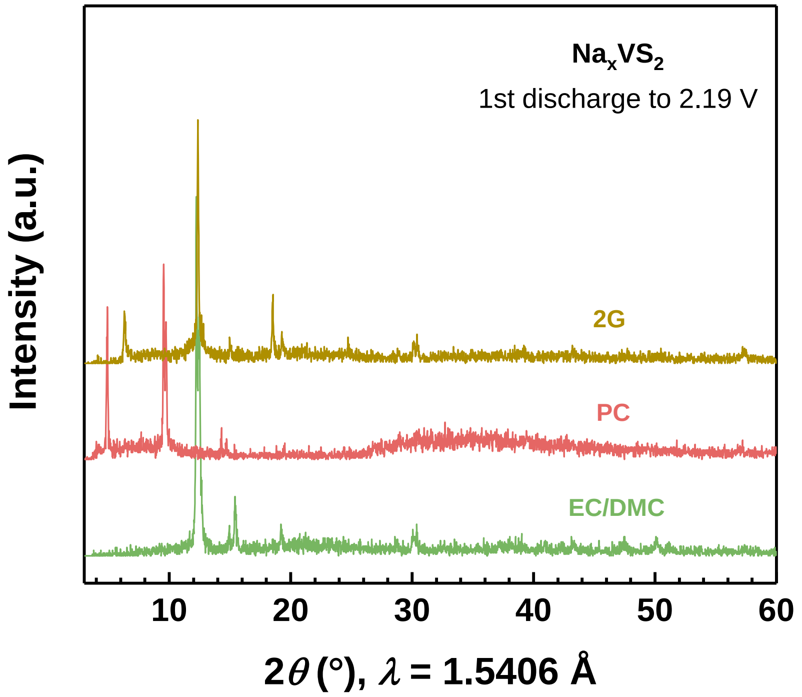

Supplementary Fig. 39 *Ex-situ* XRD of the P3-Na_xVS_2 electrode sodiated to 2.19 V *vs*. Na^+/Na in three different electrolytes in the first cycle with a voltage window of 1.5–2.8 V *vs*. Na^+/Na.

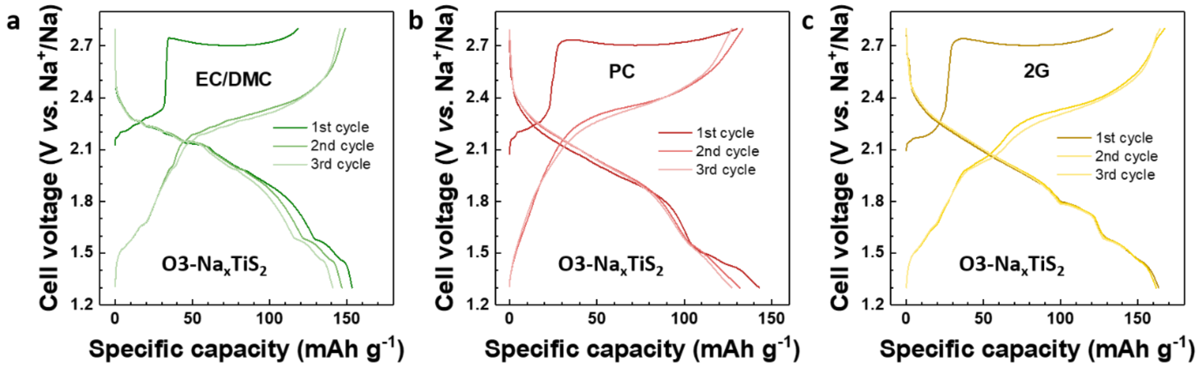

Supplementary Fig. 40 Voltage profiles of the O3-Na_xTiS_2 in different electrolytes with a voltage window of 1.3–2.8 V *vs*. Na^+/Na at 0.1C.

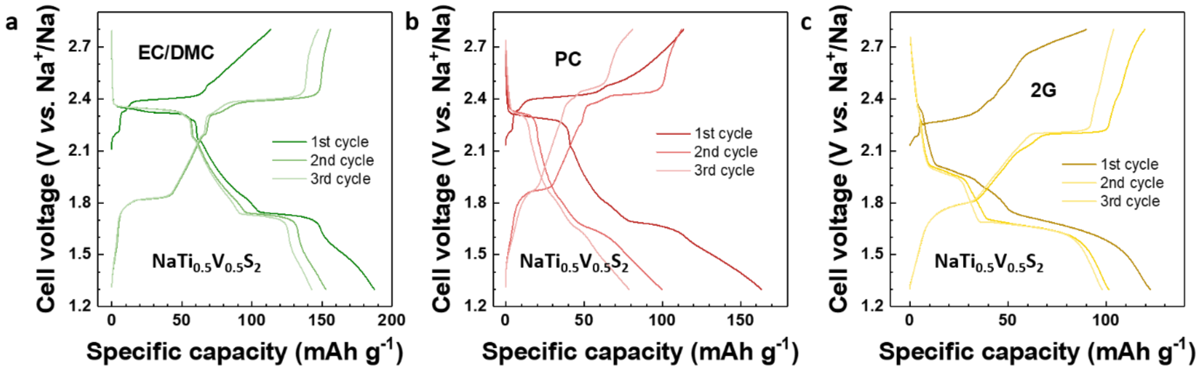

Supplementary Fig. 41 Voltage profiles of the $NaTi_{0.5}V_{0.5}S_2$ in different electrolytes with a voltage window of 1.3–2.8 V *vs.* Na^+/Na at 0.1C.

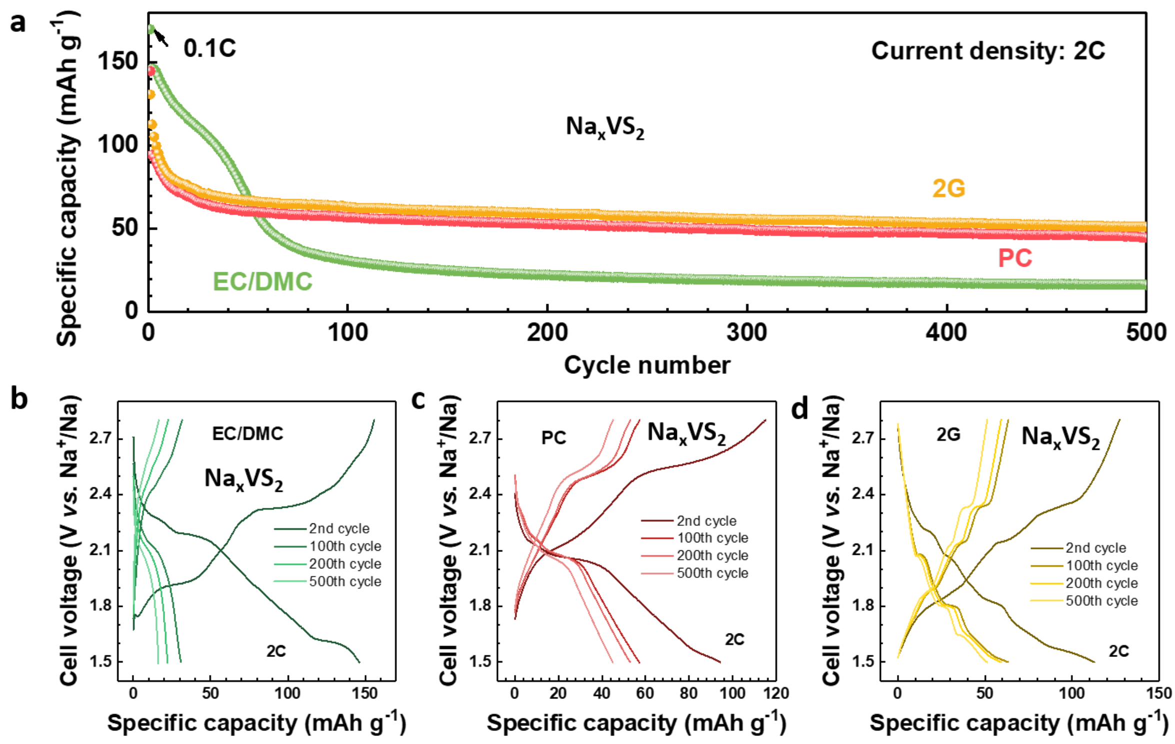

Supplementary Fig. 42 (a) Long-term cycling of Na_xVS_2 in different electrolytes at a current density of 2C. (b–d) Voltage profiles of Na_xVS_2 in different electrolytes for the 2nd, 100th, 200th, and 500th cycles at 2C. 1C corresponds to 0.2 A g^{-1}.

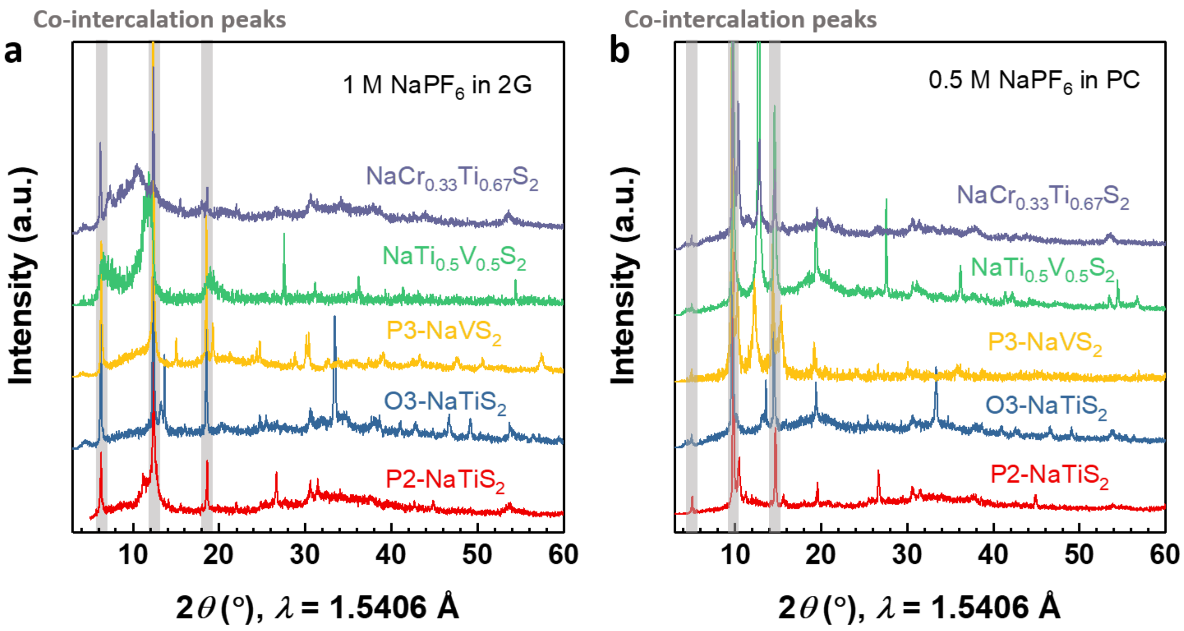

Supplementary Fig. 43 *Ex-situ* XRD of different layered compounds (P2-Na_xTiS_2, O3-Na_xTiS_2, P3-Na_xVS_2, $NaTi_{0.5}V_{0.5}S_2$, and $NaCr_{0.33}Ti_{0.67}S_2$) in (a) 1 M $NaPF_6$ in 2G electrolyte and (b) 0.5 M $NaPF_6$ in PC electrolyte. Different compounds show similar interlayer spacing with the same electrolyte solvent (either 2G or PC).

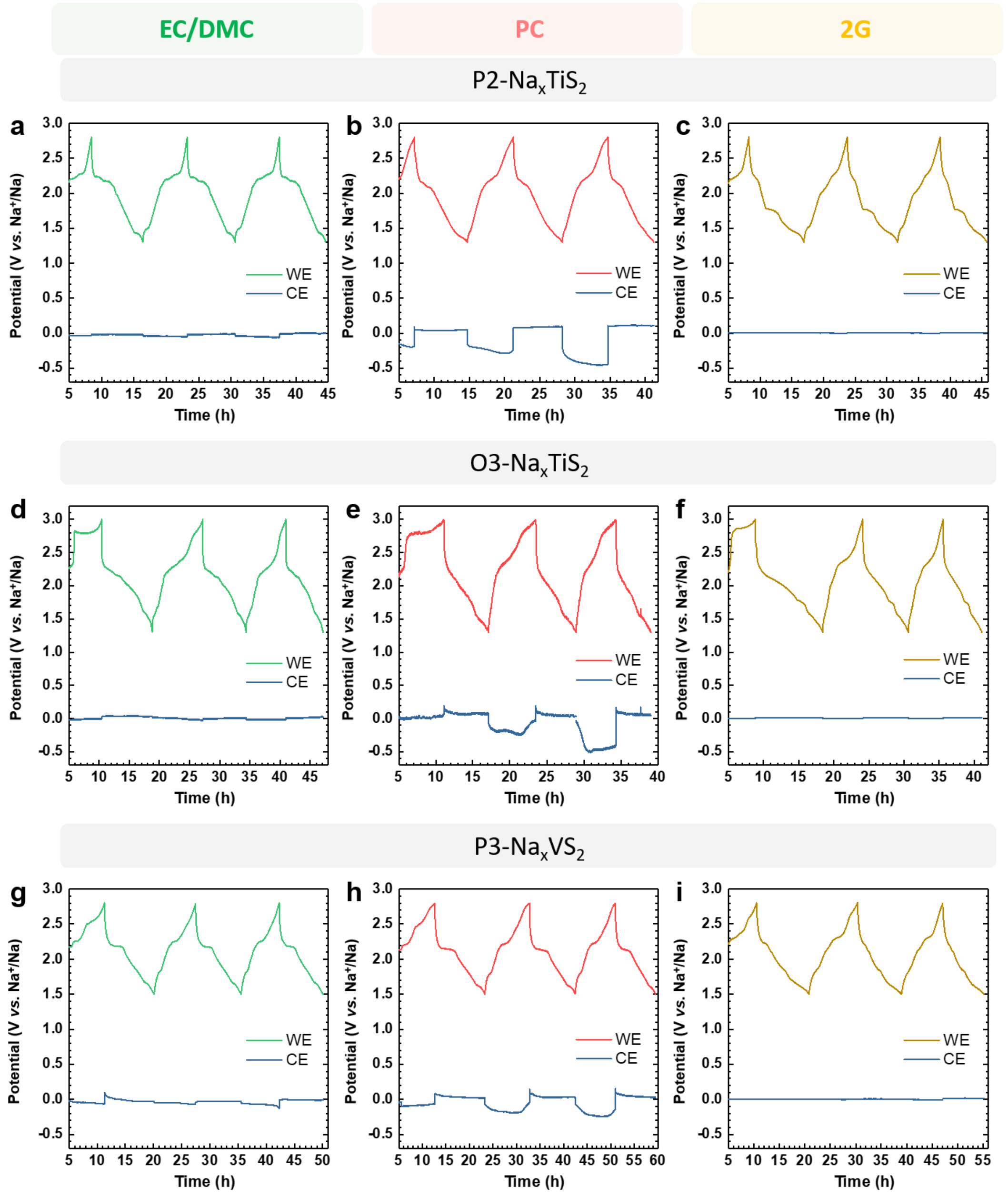

Supplementary Fig. 44 Voltage profiles of three-electrode cells of P2-Na_xTiS_2 (a–c), O3-Na_xTiS_2 (d–f), and P3-Na_xVS_2 (g–i) in 1 M $NaPF_6$ in EC/DMC electrolyte, 0.5 M $NaPF_6$ in PC electrolyte, and 1.0 M $NaPF_6$ in 2G electrolyte at 0.1C.

Results for three different solvents (EC/DMC, PC, 2G) and three different structures (P2-Na_xTiS_2, O3-Na_xTiS_2, P3-Na_xVS_2) over three cycles are shown in Supplementary Fig. 44. It can be clearly seen that Na counter electrode shows very different behavior in the different electrolytes. For 2G and EC/DMC, the polarization of the Na counter electrode is negligible in 2G and relatively small in EC/DMC, indicating that the hysteresis is largely due to the working electrode. This means that results from coin cells do provide a clear picture on the co-intercalation behavior in these two electrolytes. For PC, the situation is different. In PC electrolytes, the Na counter electrode shows a strong and rising polarization. The behavior in PC is also somewhat erratic but polarization even up to around 500 mV is found which overall indicates an unstable and resistive interface. This is known of PC electrolytes.[21] For cells

containing PC as solvent, this means that the hysteresis found for coin-cell experiments is partially caused by the Na counter electrode. It is worth noting that PC-based electrolyte also shows more side reactions compared to 2G-based electrolytes as shown by DEMS.[21] Overall, the influence of the counter electrode on the voltage hysteresis can be ranked PC >> EC/DMC > 2G.

The voltage hysteresis for the different combinations of solvents and materials are compared in Supplementary Fig. 45 and 46 (coin cells). It can be understood that co-intercalation causes hysteresis, probably because of limited diffusion. In case of PC, additional polarization occurs due to the counter electrode which overall leads to the largest hysteresis. Furthermore, intercalation, as it is the case for the EC/DMC electrolyte, enables fast diffusion and hence the smallest hysteresis (P2-Na_xTiS_2). On the other hand, also intercalation reactions can show polarization when considering the results for the O3 and P3 structure. In fact, the difference in hysteresis between intercalation (EC/DMC) and co-intercalation (2G) for these compounds becomes very small. Overall, the influence of co-intercalation on the degree of voltage hysteresis is related to the type of layered structure, with the P2 structure being more affected than the O3 or P3 structure.

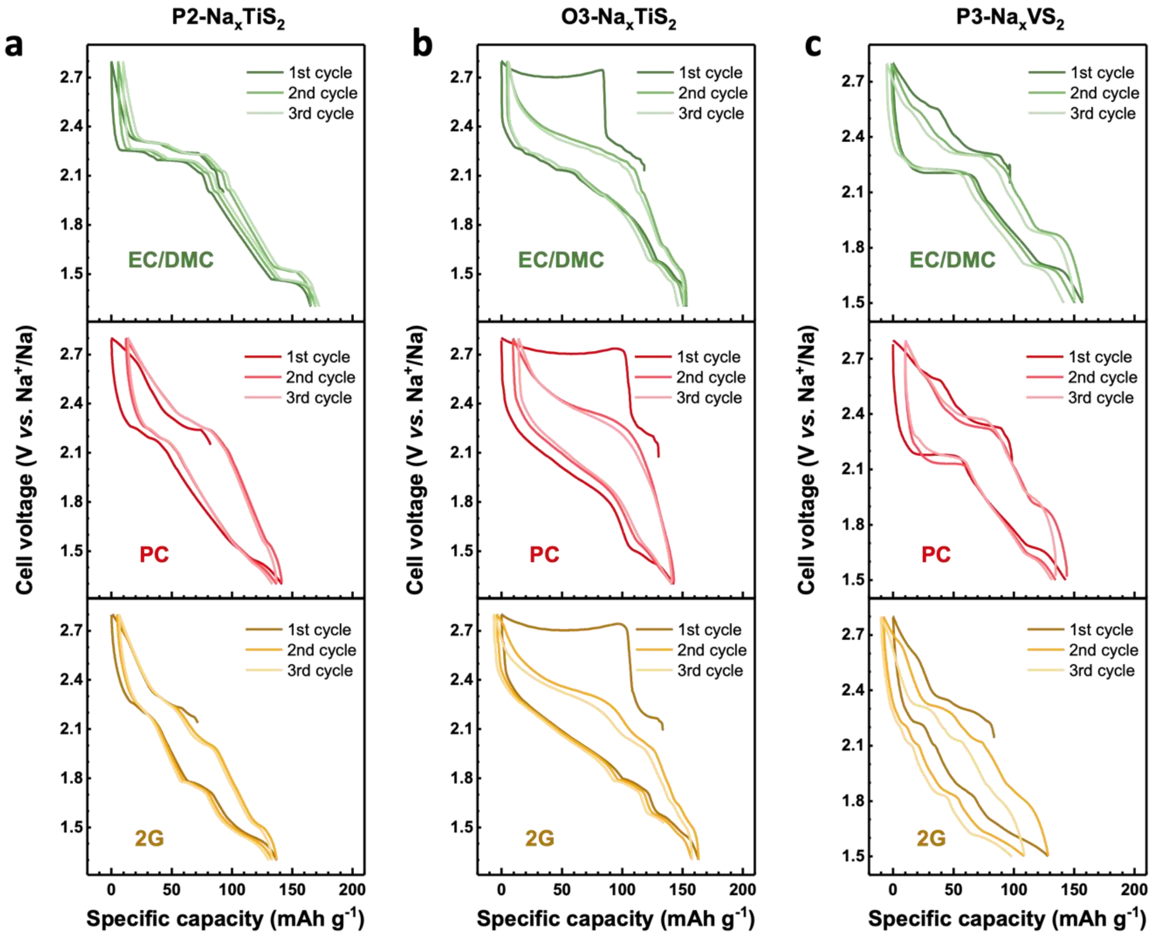

Supplementary Fig. 45 Voltage hysteresis of P2-Na_xTiS_2 (a), O3-Na_xTiS_2 (b), and P3-Na_xVS_2 (c) in 1 M $NaPF_6$ in EC/DMC electrolyte, 0.5 M $NaPF_6$ in PC electrolyte, and 1.0 M $NaPF_6$ in 2G electrolyte at 0.1C.

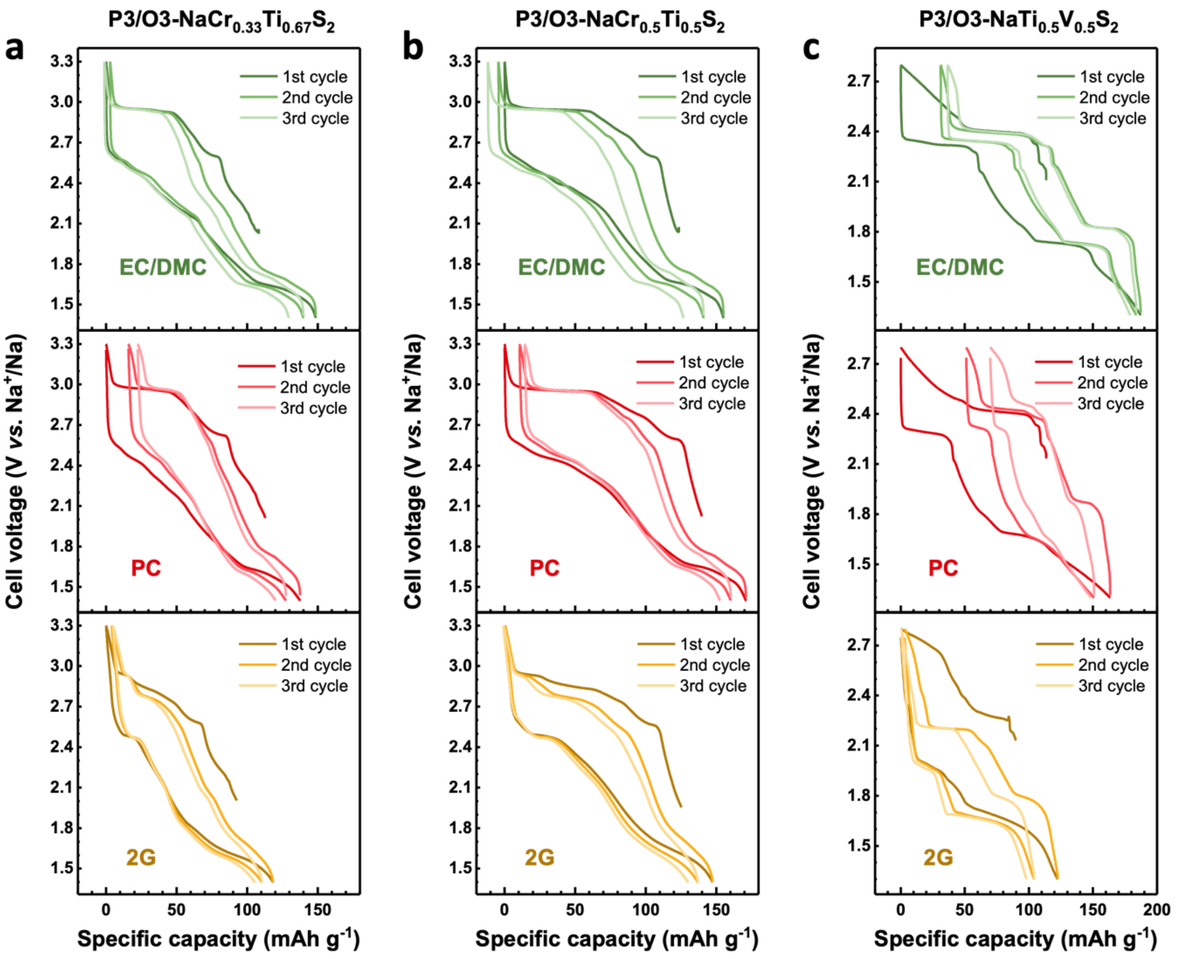

Supplementary Fig. 46 Voltage hysteresis of P3/O3-$NaCr_{0.33}Ti_{0.67}S_2$ (a), P3/O3-$NaCr_{0.5}Ti_{0.5}S_2$ (b), and P3/O3-$NaTi_{0.5}V_{0.5}S_2$ (c) in 1 M $NaPF_6$ in EC/DMC electrolyte, 0.5 M $NaPF_6$ in PC electrolyte, and 1.0 M $NaPF_6$ in 2G electrolyte at 0.1C.

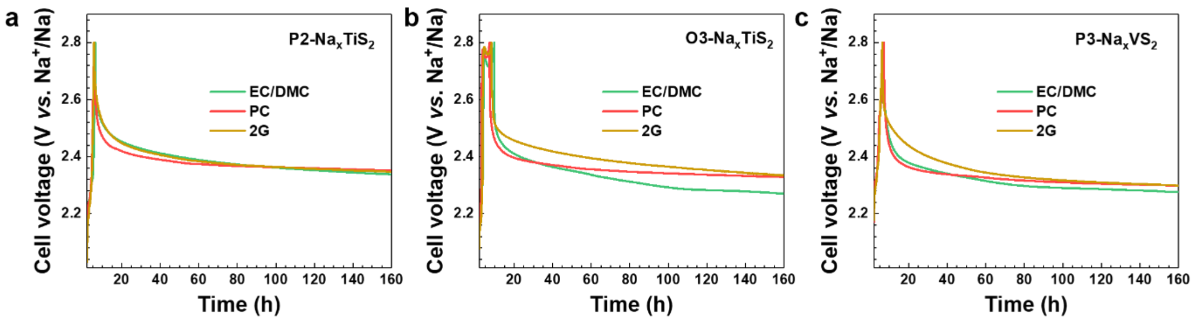

Supplementary Fig. 47 Voltage retention of P2-Na_xTiS_2 (a), O3-Na_xTiS_2 (b), and P3-Na_xVS_2 (c) in 1 M $NaPF_6$ in EC/DMC, 0.5 M $NaPF_6$ in PC, and 1.0 M $NaPF_6$ in 2G electrolytes when resting the cells at fully desodiated state (2.8 V *vs.* Na^+/Na).

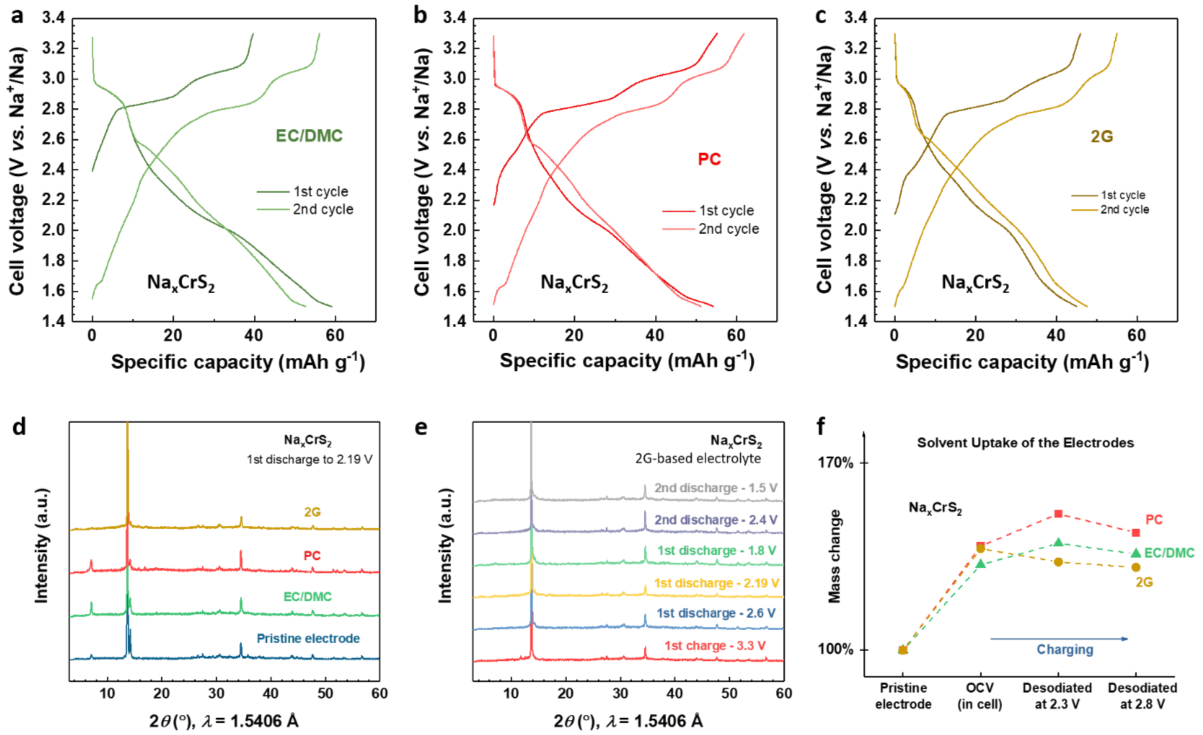

Supplementary Fig. 48 (a–c) Voltage profiles of the Na_xCrS_2 in different electrolytes with a voltage window of 1.5–3.3 V *vs*. Na^+/Na at 0.1C. *Ex-situ* XRD of the Na_xVS_2 electrode sodiated to 2.19 V *vs*. Na^+/Na in three different electrolytes in the first cycle (d) and at different charge states in the 2G-based electrolytes (e). (f) Mass uptake at different desodiation states in Na_xCrS_2.

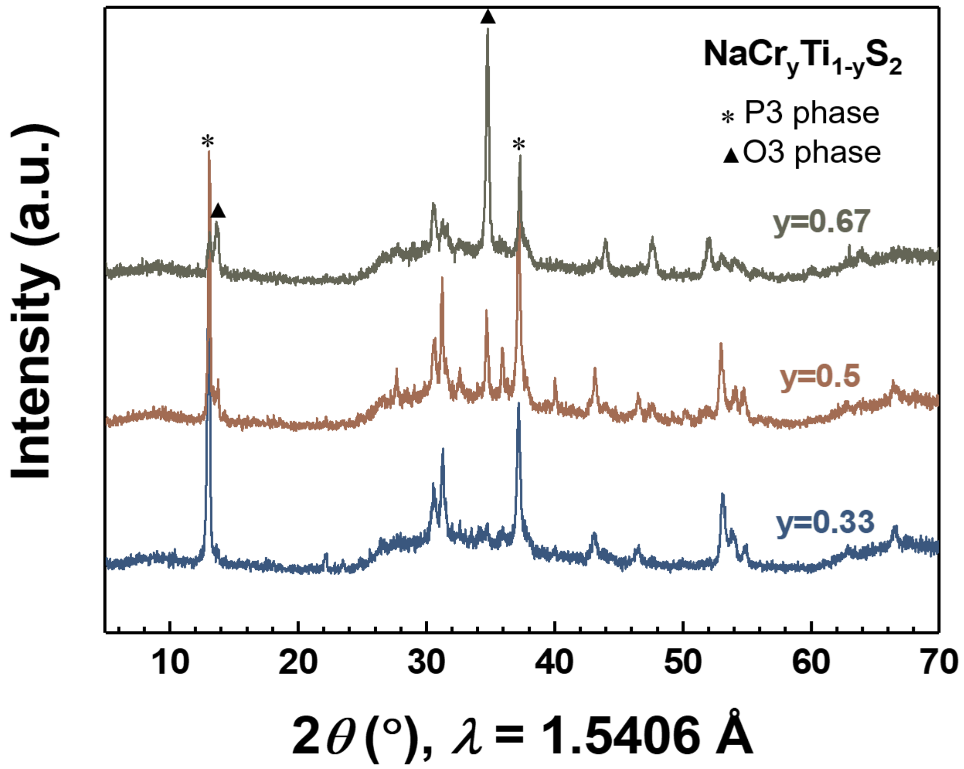

Supplementary Fig. 49 XRD patterns of the synthesized $NaCr_yTi_{1-y}S_2$ (y = 0.33, 0.5, or 0.67) powder.

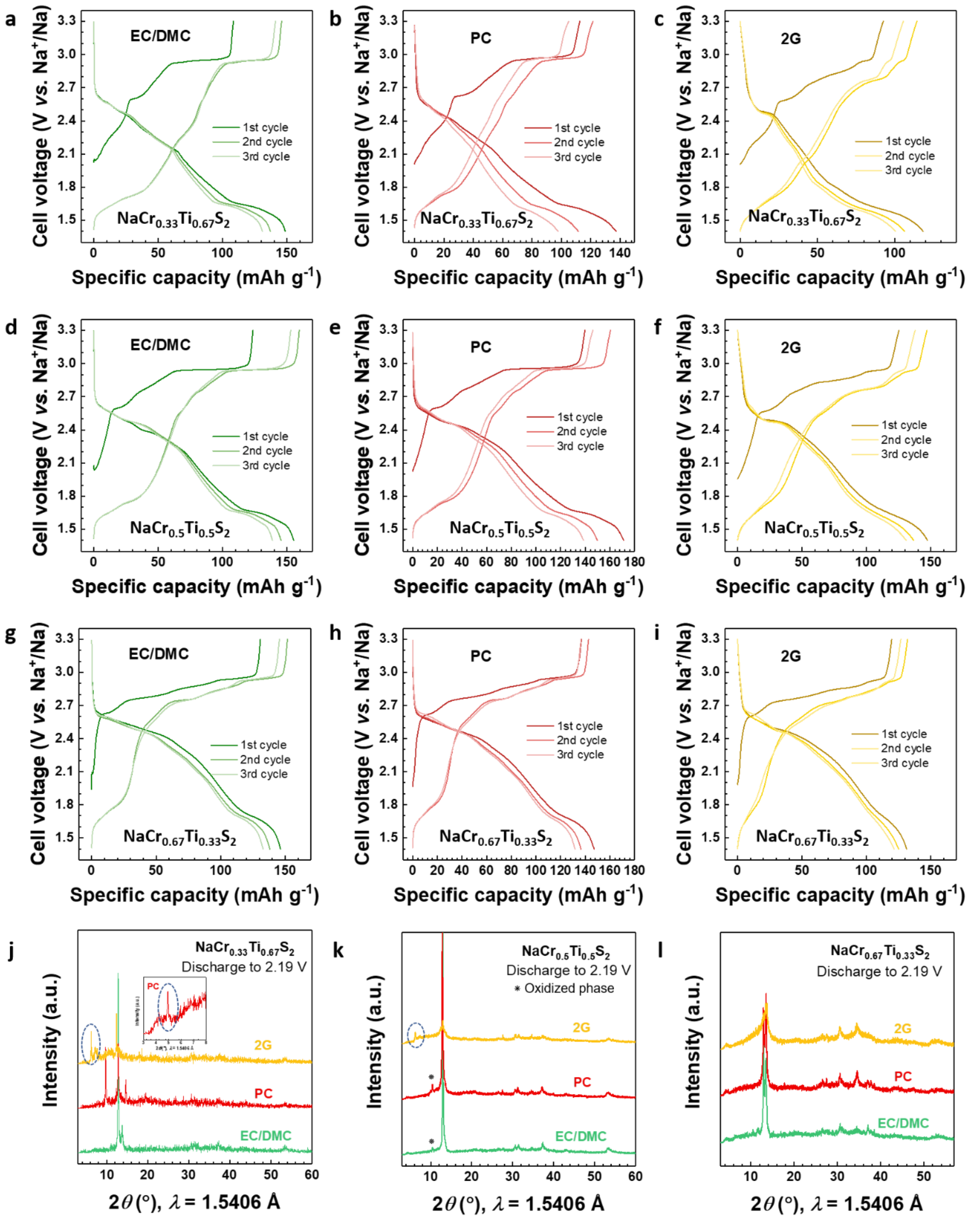

Supplementary Fig. 50 (a–i) Voltage profiles of the $NaCr_yTi_{1-y}S_2$ (y = 0.33, 0.5, or 0.67) in different electrolytes with a voltage window of 1.4–3.3 V *vs*. Na^+/Na at 0.1C. (j–l) *Ex-situ* XRD of the $NaCr_yTi_{1-y}S_2$ (y = 0.33, 0.5, or 0.67) electrode sodiated to 2.19 V *vs*. Na^+/Na in three different electrolytes.

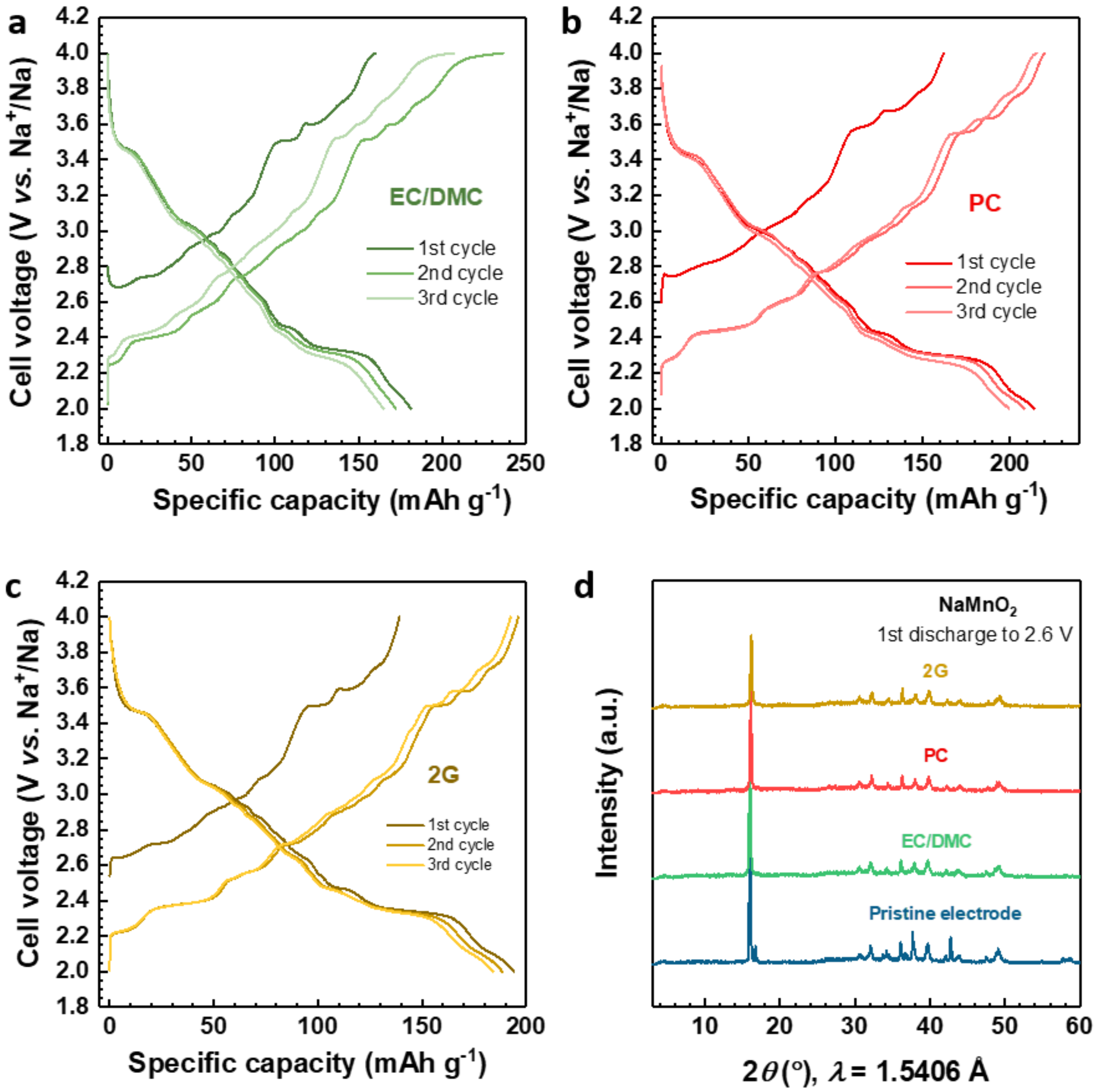

Supplementary Fig. 51 (a–c) Voltage profiles of the $NaMnO_2$ in different electrolytes with a voltage window of 1.3–2.8 V at 0.1C. (d) *Ex-situ* XRD of the $NaMnO_2$ electrode sodiated to 2.6 V *vs*. Na^+/Na in three different electrolytes in the first cycle.

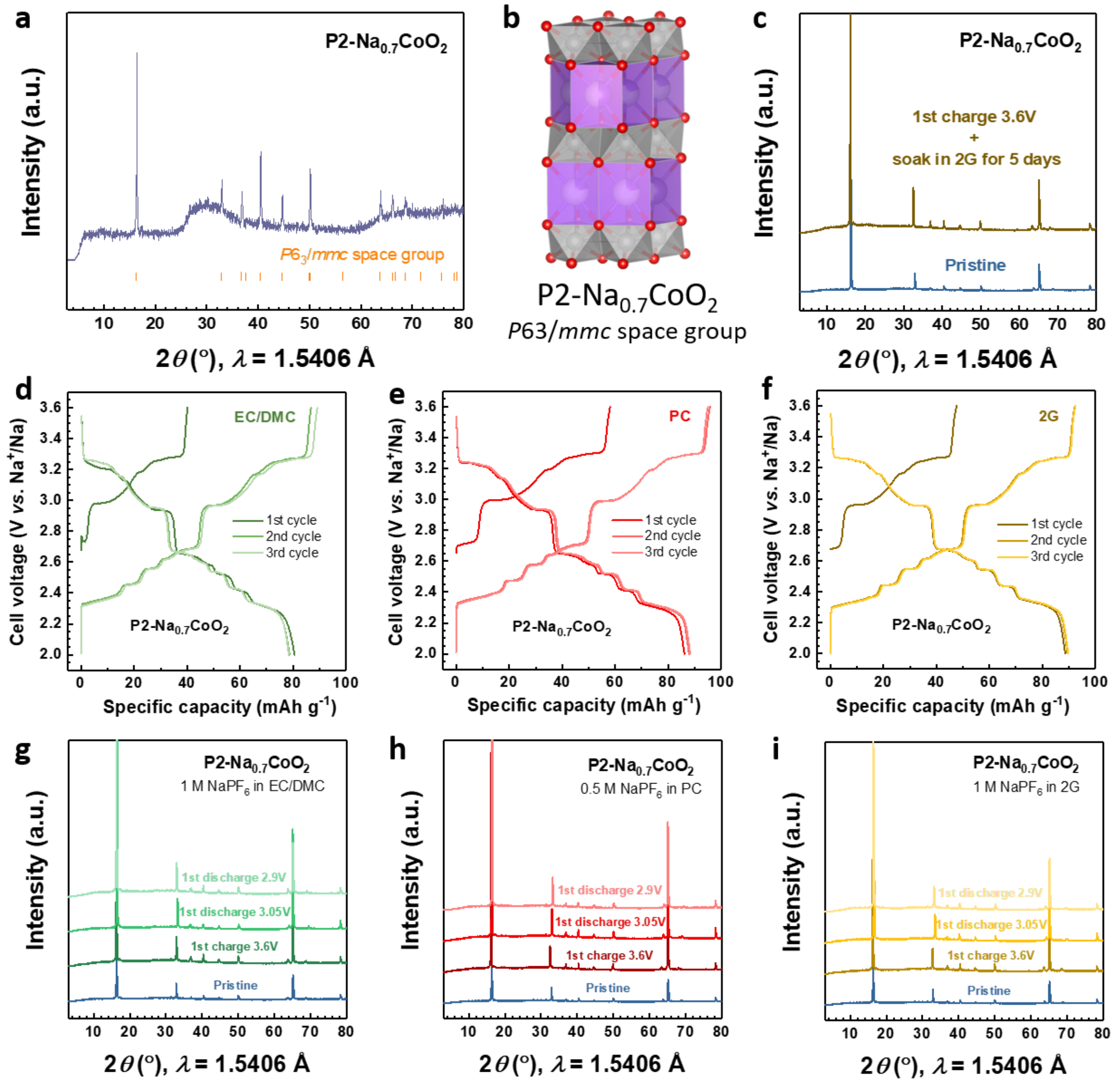

Supplementary Fig. 52 (a) XRD pattern of the synthesized $Na_{0.7}CoO_2$. The synthesized compound has a single P2 phase. (b) Crystal structure of P2-$Na_{0.7}CoO_2$ with *P*6_3/*mmc* space group (ICSD 163249). (c) XRD pattern of the P2-$Na_{0.7}CoO_2$ electrode desodiated to 3.6 V *vs*. Na^+/Na in the 2G-based electrolyte and then soaked in the 2G solvent for 5 days. (d–f) Voltage profiles of the P2-$Na_{0.7}CoO_2$ in different electrolytes at 0.1C. (g–i) *Ex-situ* XRD of the P2-$Na_{0.7}CoO_2$ electrode at different desodiation/sodiation states (pristine at OCV, desodiated to 3.6 V *vs*. Na^+/Na, sodiated to 3.05 V *vs*. Na^+/Na, and sodiated to 2.9 V *vs*. Na^+/Na) in three different electrolytes in the first cycle. No intercalation is observed in all three electrolytes.

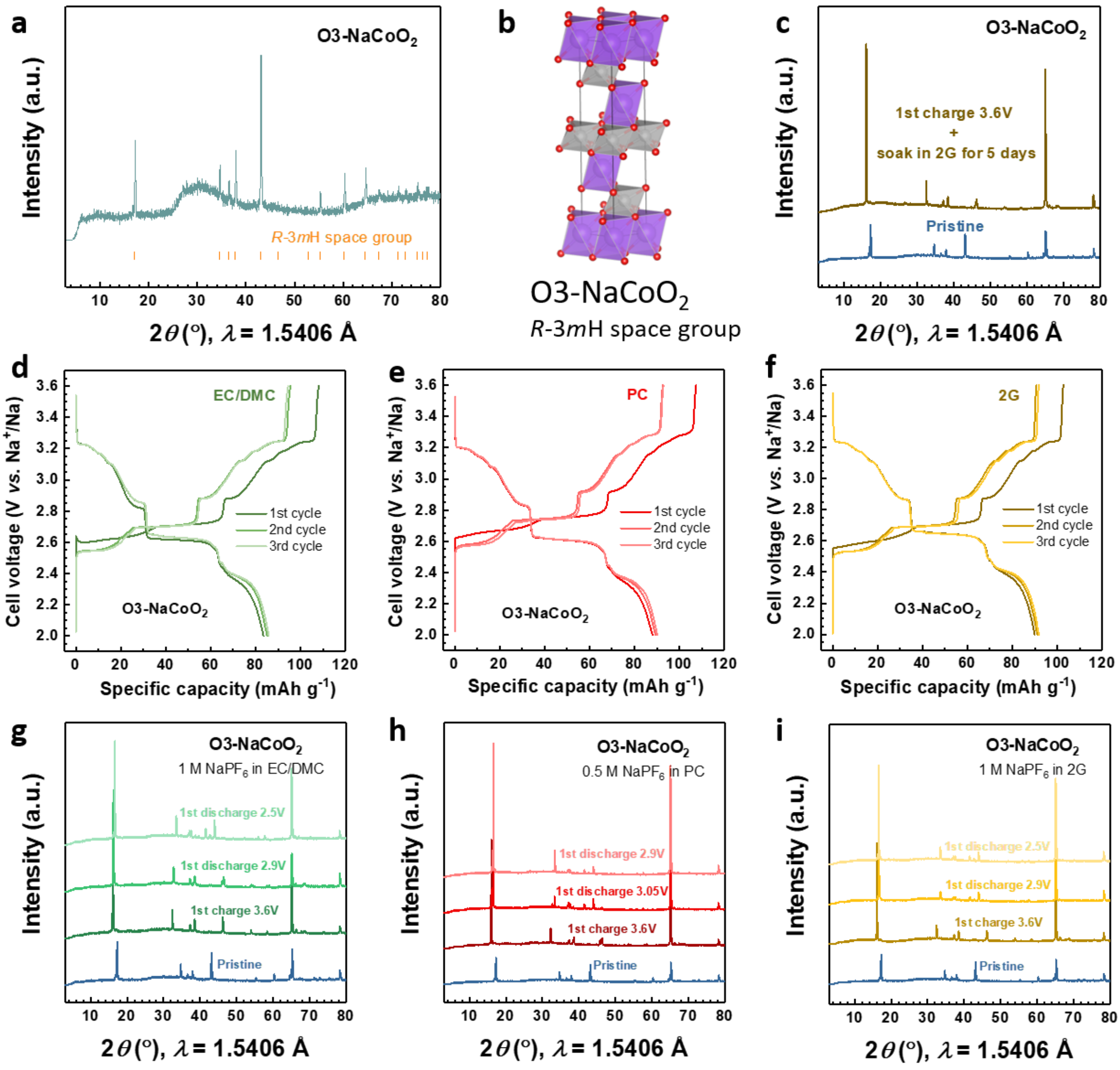

Supplementary Fig. 53 (a) XRD pattern of the synthesized $NaCoO_2$. The synthesized compound has a single O3 phase. (b) Crystal structure of O3-$NaCoO_2$ with *R*-3*mH* space group (ICSD 96428). (c) XRD pattern of the O3-$NaCoO_2$ electrode desodiated to 3.6 V *vs*. Na^+/Na in the 2G-based electrolyte and then soaked in the 2G solvent for 5 days. (d–f) Voltage profiles of the O3-$NaCoO_2$ in different electrolytes at 0.1C. (g–i) *Ex-situ* XRD of the O3-$NaCoO_2$ electrode at different desodiation/sodiation states (pristine at OCV, desodiated to 3.6 V *vs*. Na^+/Na, sodiated to 3.05 V *vs*. Na^+/Na, and sodiated to 2.9 V *vs*. Na^+/Na) in three different electrolytes in the first cycle. No intercalation is observed in all three electrolytes.

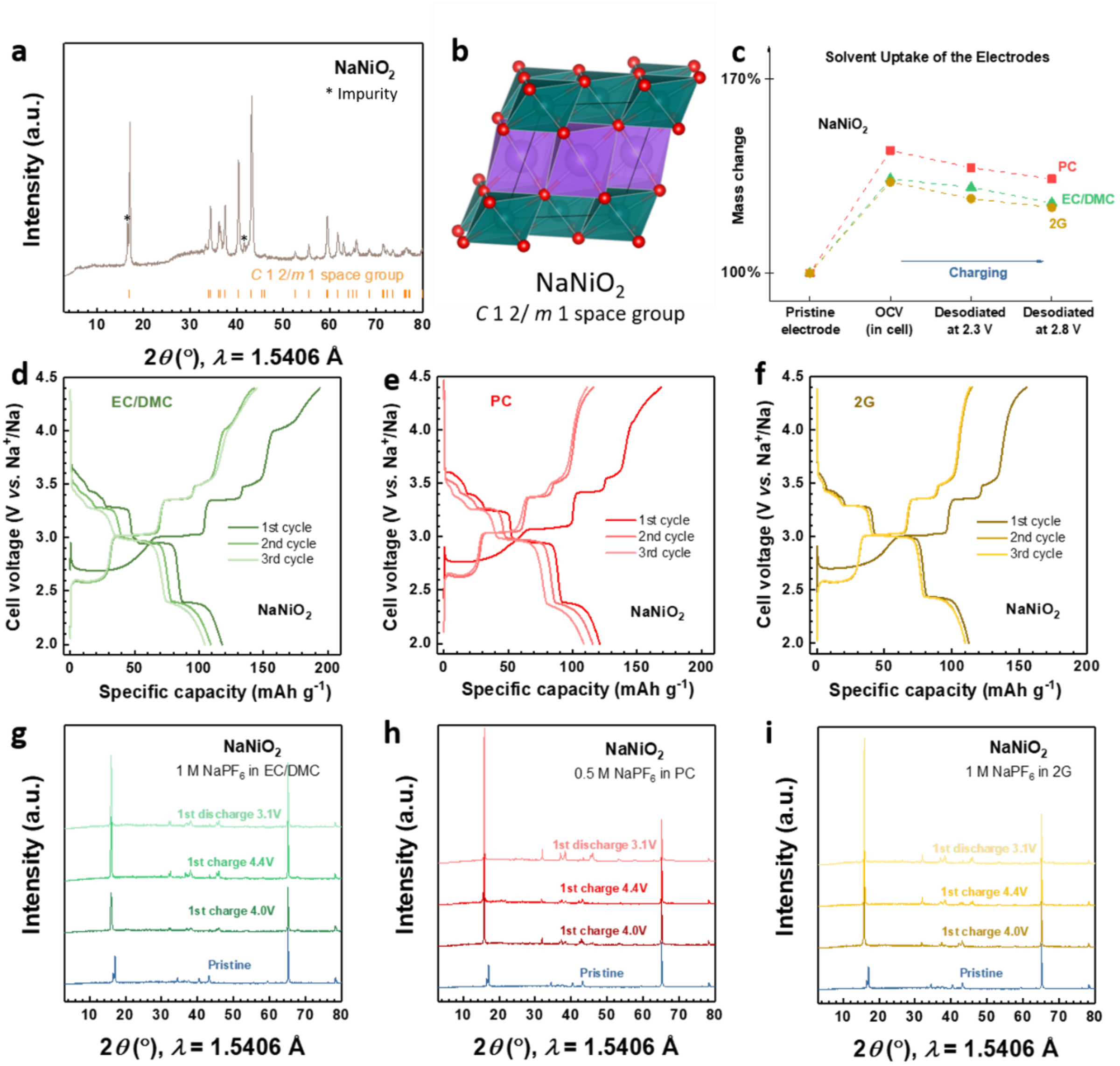

Supplementary Fig. 54 (a) XRD pattern of the synthesized $NaNiO_2$. An impurity phase is marked with "*", which is attributed to some Na loss during synthesis. (b) Crystal structure of $NaNiO_2$ with *C* 1 2/ *m* 1 space group (ICSD 154172). (c) Mass uptake at different desodiation states in $NaNiO_2$. (d–f) Voltage profiles of the $NaNiO_2$ in different electrolytes at 0.1C. (g–i) *Ex-situ* XRD of the $NaNiO_2$ electrode at different desodiation/sodiation states (pristine at OCV, desodiated to 4.0 V *vs.* Na^+/Na, desodiated to 4.4 V *vs.* Na^+/Na, and sodiated to 3.1 V *vs.* Na^+/Na) in three different electrolytes in the first cycle. No intercalation is observed in all three electrolytes.

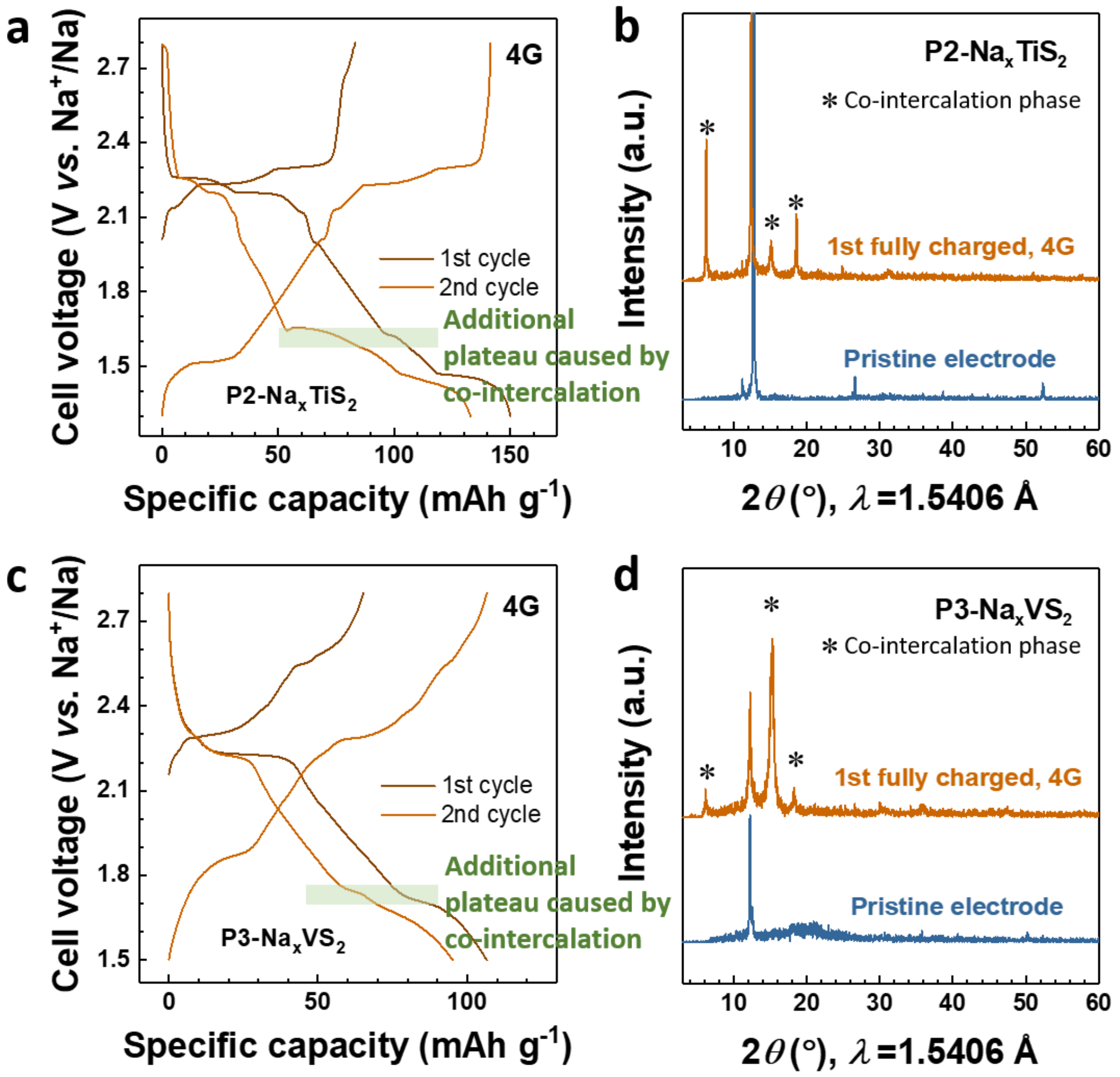

Supplementary Fig. 55 Voltage profiles (cycled at 0.1C) and *ex-situ* XRD patterns of P2-Na_xTiS_2 (a–b) and P3-Na_xVS_2 (c–d) in 1 M $NaPF_6$ in 4G electrolyte.

Apart from the investigated 2G co-intercalation in CAMs, co-intercalation was also explored and found in the 4G-based electrolyte. As shown in Supplementary Fig. 55, both P2-Na_xTiS_2 and P3-Na_xVS_2 show different electrochemical behaviors and an expanded structure in the fully desodiated state in the first cycle. Therefore, in addition to 2G, 4G that belong to the ethereal family also shows the property for the occurrence of the co-intercalation in layered sulfides.

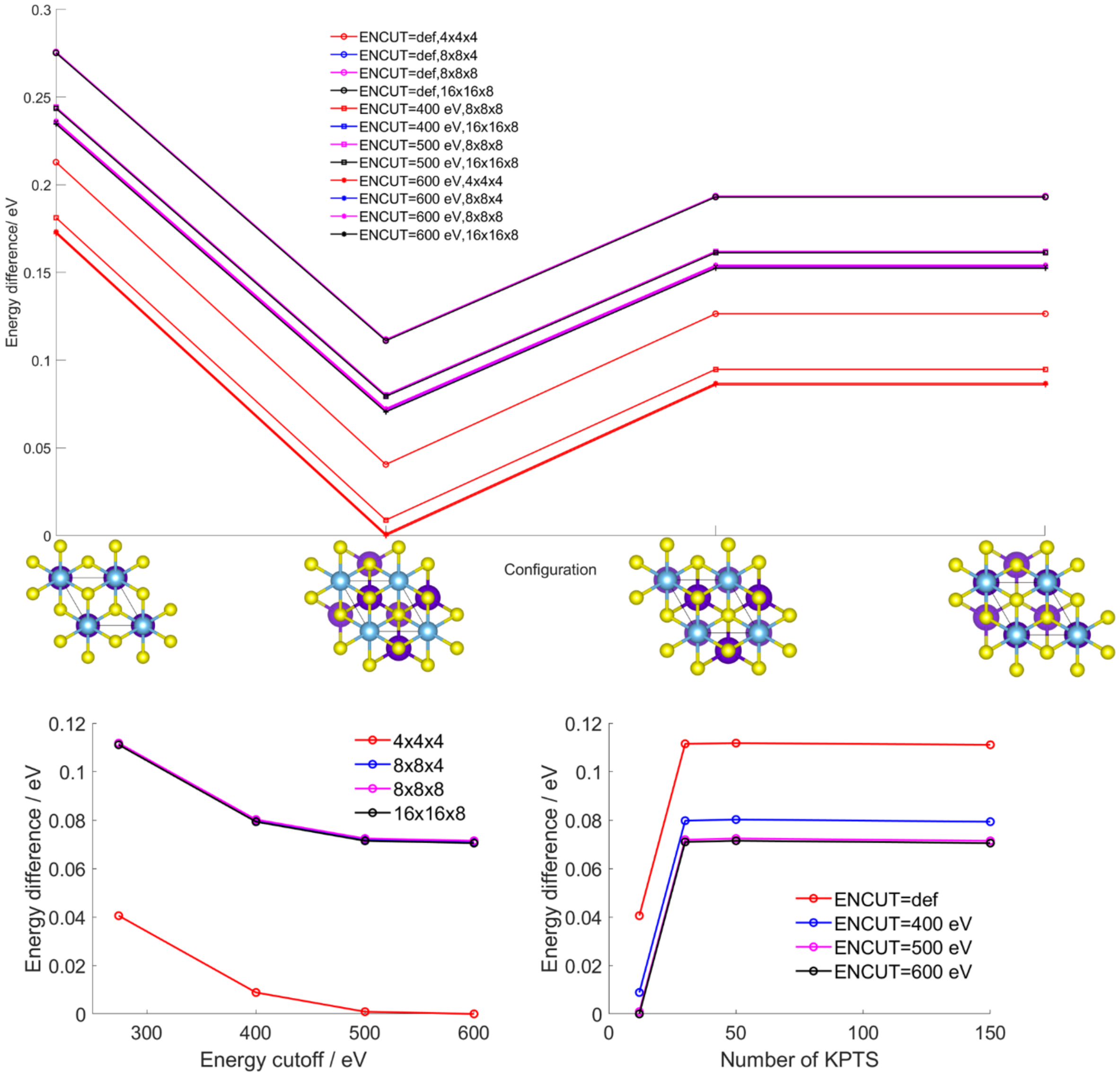

Supplementary Fig. 56 (a) Energies of the 4 starting configurations at the tested cutoff energies and k-point grids. (b) Energy differences with respect to energy cutoff, (c) energy differences with respect to k-point grid sampling.

Supplementary Tables

Supplementary Table 1 Structural parameters for the pristine P2-Na_xTiS_2 compound obtained from Rietveld refinement of the XRD pattern.

**Space group: *P* 6$_3$/*m m c***					
a	***b***	***c***	***α***	***β***	***γ***
3.461(84) Å	3.461(84) Å	13.920(56) Å	90°	90°	120°
**Atoms**	***x***	***y***	***z***	**Occ.**	**B_{iso}**
**Ti1**	0	0	0	0.989	0.012
**Na1**	0.3333	0.6667	0.75	0.370	0.0156
**Na2**	0	0	0.25	0.293	0.011
**S1**	0.3333	0.6667	0.09881	1.004	0.0099
**R_{wp}**	1.585%		**GOF**	4.86	

Supplementary Table 2 Inductively Coupled Plasma Optical Emission spectroscopy (ICP-OES) results for pristine Na_xMS_2 (M = Ti, V, or Cr) compounds.

Compounds	Relative Na content *vs*. transition metal (M)
**P2-Na_xTiS_2**	0.94
**P3-Na_xVS_2**	0.73
**O3-Na_xTiS_2**	0.87
**O3-Na_xCrS_2**	0.80

Supplementary Table 3 Structural parameters for the P2-Na_xTiS_2 electrode with an OP4 phase obtained from Rietveld refinement of the XRD pattern.

**Space group: *P* 6₃/*m m c***					
a	***b***	***c***	***α***	***β***	***γ***
3.410(8) Å	3.410(8) Å	25.657(16) Å	90°	90°	120°
**Atoms**	***x***	***y***	***z***	**Occ.**	**B_{iso}**
**Ti1**	0.666667	0.333333	0.3822	0.894	0.0546
**S1**	0.333333	0.666667	0.44925	1.027	0.0382
**S2**	0	0	0.3209	0.967	0.0455
**Na1**	0.666667	0.333333	0.25	1.077	0.0459
**Na2**	0	0	0.5	0.642	0.0892
R_{wp}	2.313%		**GOF**	2.98	

Supplementary Table 4 Structural parameters for the P2-Na_xTiS_2 electrode with an O2 phase obtained from Rietveld refinement of the XRD pattern.

**Space group: *P* 6$_3$ *m c***					
a	***b***	***c***	***α***	***β***	***γ***
3.403(51) Å	3.403(51) Å	11.694(37) Å	90°	90°	120°
**Atoms**	***x***	***y***	***z***	**Occ.**	**B_{iso}**
**Ti1**	0.333333	-0.333333	-0.00887	1.114	0.00232
**Na1**	0.333333	-0.333333	0.256148	0.182	0.04221
**S1**	0.666667	0.333333	0.094583	1.012	0.01498
**S3**	1	0	0.908698	0.992	0.01498
**R_{wp}**	1.744%		**GOF**	2.25	

Supplementary Table 5 ^{13}C NMR shift values (in ppm) for the CH_2 and CH_3 groups of diglyme and the shift difference (Δδ) to the bulk electrolyte (outside layered structure) peak for 2.8 V and 1.75 V *vs*. Na^+/Na.

**2.8 V *vs.* Na^+/Na**	**CH_2**	**Δδ**	**CH_2**	**Δδ**	**CH_3**	**Δδ**
**Bulk electrolyte (outside layered structure)**	72.5	-	70.8	-	59.2	-
**Solvated Na^+ (in layered structure)**	77.1	**4.6**	75.7	**4.9**	64.1	**4.9**
**Free solvent (in layered structure)**	71.6	–0.9	70.0	–0.8	58.3	–0.8
**1.75 V *vs.* Na^+/Na**	**CH_2**	**Δδ**	**CH_2**	**Δδ**	**CH_3**	**Δδ**
**Bulk electrolyte (outside layered structure)**	72.6	-	70.9	-	59.1	-
**Solvated Na^+ (in layered structure)**	79.1	**6.5**	77.5	**6.6**	66.1	**7.0**
**Free solvent (in layered structure)**	71.8	–0.8	70.0	–0.9	58.3	–0.8

Supplementary Table 6 Fitting of Peaks for ^{13}C MAS NMR spectrum of 2.8V sample

#	ppm	Height	Width(Hz)	L/G	Area
1	78.46	10048.53	102	0.00	341836646
2	77.07	19189.89	99	0.00	638868988
3	75.71	19511.43	93	0.00	605840370
4	74.27	18023.98	105	0.00	632908864
5	73.94	3531.82	21	0.00	24361677
6	73.06	17534.57	88	0.53	649019479
7	72.53	10791.50	25	0.04	93781721
8	72.24	5190.39	20	0.00	34362901
9	71.60	34038.78	114	0.00	1924847866
10	71.13	4972.88	22	0.45	44293370
11	70.81	10193.32	18	0.56	76550701
12	70.03	30286.59	104	0.41	1256696881
13	69.41	3881.06	17	0.01	22560294
14	68.57	9025.39	85	0.00	258268856
15	66.25	3633.71	54	0.44	79525715
16	64.79	12777.93	99	0.53	531938557
17	63.43	12935.39	86	0.46	452570989
18	62.13	3624.16	59	0.00	71827274
19	61.36	3059.65	23	0.52	29915925
20	60.61	3001.61	52	0.00	52413871
21	59.94	7421.52	25	0.40	73218731
22	59.11	13667.65	81	0.83	520101727
23	58.54	8440.23	26	0.52	92176723
24	57.67	13853.09	85	0.47	482626160
25	57.15	3412.59	24	0.75	36828425
26	56.24	4293.33	78	0.00	111528084

Supplementary Table 7 Fitting of Peaks for ^{13}C MAS NMR spectrum of 1.75V sample

#	ppm	Height	Width(Hz)	L/G	Area
1	80.53	2182.83	160	0.50	144762119
2	79.13	4082.09	160	0.50	270718272
3	78.91	2036.47	160	0.50	135055871
4	77.91	2036.47	160	0.50	135055871
5	77.48	4005.08	160	0.50	265611172
6	76.38	2036.47	160	0.50	135055871
7	73.95	8514.37	28	0.00	116632115
8	73.21	22238.80	92	0.40	956002988
9	72.55	15643.72	28	0.00	216440785
10	72.27	9410.44	28	0.00	130199367
11	71.76	56447.50	97	0.30	2603744270
12	71.36	17819.30	69	0.32	476432038
13	71.16	7728.66	28	0.00	106930982
14	70.87	14087.87	28	0.00	194914562
15	70.51	23810.61	76	0.31	696807373
16	70.03	55452.67	84	0.36	2182473828
17	69.45	8035.72	28	1.00	111179241
18	68.67	21858.98	100	0.13	1061735177
19	66.77	5597.11	160	0.50	412868789
20	68.11	2748.40	160	0.50	182270155
21	65.38	5692.19	160	0.50	419882570
22	64.03	2690.28	160	0.50	178415328
23	61.18	2467.84	17	0.00	20175042
24	60.41	6356.55	69	0.41	204221888
25	59.79	6048.20	21	0.00	63567143
26	58.97	21496.37	100	0.24	1031119178
27	58.42	5394.72	18	0.00	46959514
28	57.54	24789.42	104	0.25	1231944160
29	56.96	2210.87	15	0.00	16441855
30	56.20	8356.82	97	0.50	374772189

Supplementary Table 8 Fitting of Peaks for ^{13}C MAS NMR spectrum of 1.3V sample

#	ppm	Height	Width(Hz)	L/G	Area
1	73.20	7528.33	160	0.50	499267978
2	71.76	13261.00	160	0.50	879450279
3	71.36	7385.90	160	0.50	489822455
4	70.51	7243.48	160	0.50	480376932
5	70.03	13403.42	160	0.50	888895802
6	68.71	6552.65	160	0.50	434562389
7	60.46	2970.68	160	0.50	197011241
8	58.93	7347.95	160	0.50	487305559
9	57.58	7243.48	160	0.50	480376932
10	56.17	3255.53	160	0.50	215902287

Supplementary Table 9 NMR parameters used for MAS NMR data collection

**Nucleus**	^{13}**C**	^{23}**Na**
**Recycle Delay (s)**	5	0.1
**Transients**	31931–51626	33440
**π/2 Pulse Length (µs)**	4	1.7

References

[1] Lei, Y., Li, X., Liu, L. & Ceder, G. Synthesis and stoichiometry of different layered sodium cobalt oxides. *Chem. Mater.* **26**, 5288–5296 (2014).

[2] Viciu, L., Bos, J. W. G., Zandbergen, H. W., Huang, Q., Foo, M. L., Ishiwata, S., Ramirez, A. P., Lee, M., Ong, N. P. & Cava, R. J. Crystal structure and elementary properties of Na_xCoO_2 (x = 0.32, 0.51, 0.6, 0.75, and 0.92) in the three-layer $NaCoO_2$ family. *Phys. Rev. B* **73**, 174104 (2006).

[3] Bianchini, M., Wang, J., Clément, R. J., Ouyang, B., Xiao, P., Kitchaev, D., Shi, T., Zhang, Y., Wang, Y., Kim, H., Zhang, M., Bai, J., Wang, F., Sun, W. & Ceder, G. The interplay between thermodynamics and kinetics in the solid-state synthesis of layered oxides. *Nat. Mater.* **19**, 1088–1095 (2020).

[4] Wiedemann, D. E., Lerch, M. & Suard, E. Structural complexities and sodium-ion diffusion in the intercalates Na_xTiS_2: move it, change it, re-diffract it. *RSC Adv.* **9**, 27780–27788 (2019).

[5] Lin, C.-H., Topsakal, M., Sun, K., Bai, J., Zhao, C., Dooryhee, E., Northrup, P., Gan, H., Lu, D., Stavitski, E. & Chen-Wiegart, Y. K. Operando structural and chemical evolutions of TiS_2 in Na-ion batteries. *J. Mater. Chem. A* **8**, 12339–12350 (2020).

[6] Lu, Z. & Dahn, J. R. In situ X-ray diffraction study of P2–$Na_{2/3}[Ni_{1/3}Mn_{2/3}]O_2$. *J. Electrochem. Soc.* **148**, A1225–A1229 (2001).

[7] Yabuuchi, N., Kajiyama, M., Iwatate, J., Nishikawa, H., Hitomi, S., Okuyama, R., Usui, R., Yamada, Y. & Komaba, S. P2-type $Na_x[Fe_{1/2}Mn_{1/2}]O_2$ made from earth-abundant elements for rechargeable Na batteries. *Nat. Mater.* **11**, 512–517 (2012).

[8] Wang, P.-F., You, Y., Yin, Y.-X. & Guo, Y.-G. Layered oxide cathodes for sodium-ion batteries: phase transition, air stability, and performance. *Adv. Energy Mater.* **8**, 1701912 (2018).

[9] Somerville, J. W., Sobkowiak, A., Tapia-Ruiz, N., Billaud, J., Lozano, J. G., House, R. A., Gallington, L. C., Ericsson, T., Häggström, L., Roberts, M. R., Maitra, U. & Bruce, P. G. Nature of the “Z”-phase in layered Na-ion battery cathodes. *Energy Environ. Sci.* **12**, 2223–2232 (2019).

[10] Hwang, J.-Y., Kim, J., Yu, T.-Y. & Sun, Y.-K. A new P2-type layered oxide cathode with extremely high energy density for sodium-ion batteries. *Adv. Energy Mater.* **9**, 1803346 (2019).

[11] Wang, F., Peng, B., Zeng, S., Zhao, L., Zhang, X., Wan, G., Zhang, H. & Zhang, G. Activating oxygen redox in layered Na_xMnO_2 to suppress intrinsic deficient behavior and enable phase-transition-free sodium ion cathode. *Adv. Funct. Mater.* **32**, 2202665 (2022).

[12] Wang, X., Zhang, Q., Zhao, C., Li, H., Zhang, B., Zeng, G., Tang, Y., Huang, Z., Hwang, I., Zhang, H., Zhou, S., Qiu, Y., Xiao, Y., Cabana, J., Sun, C.-J., Amine, K., Sun, Y., Wang, Q., Xu, G.-L., Gu, L., Qiao, Y. & Sun, S.-G. Achieving a high-performance sodium-ion pouch cell by regulating intergrowth structures in a layered oxide cathode with anionic redox. *Nat. Energy* **9**, 184–196 (2024).

[13] Goktas, M., Bolli, C., Berg, E. J., Novák, P., Pollok, K., Langenhorst, F., von Roeder, M., Lenchuk, O., Mollenhauer, D. & Adelhelm, P. Graphite as cointercalation electrode for sodium-ion batteries: electrode dynamics and the missing solid electrolyte interphase (SEI). *Adv. Energy Mater.* **8**, 1702724 (2018).

[14] Mali, G., Patel, M. U. M., Mazaj, M. & Dominko, R. Stable crystalline forms of Na polysulfides: experiment versus ab initio computational prediction. *Chem. Eur. J.* **22**, 3355–3360 (2016).

[15] Tegman, R. The crystal structure of sodium tetrasulphide, Na_2S_4. *Acta Cryst. B* **29**, 1463–1469 (1973).

[16] Wang, L., Wang, T., Peng, L., Wang, Y., Zhang, M., Zhou, J., Chen, M., Cao, J., Fei, H., Duan, X., Zhu, J. & Duan, X. The promises, challenges and pathways to room-temperature sodium–sulfur batteries. *Natl. Sci. Rev.* **9**, nwab050 (2022).

[17] Molinie, P., Trichet, L., Rouxel, J., Berthier, C., Chabre, Y. & Segransan, P. The Na–TiS_2 system: a critical discussion of the phase boundaries, structural and NMR studies. *J. Phys. Chem. Solids* **45**, 105–112 (1984).

[18] Leifer, N., Greenstein, F. M., Mor, A., Aurbach, D. & Goobes, G. NMR-detected dynamics of sodium co-intercalation with diglyme solvent molecules in graphite anodes linked to prolonged cycling. *J. Phys. Chem. C* **122**, 21172–21184 (2018).

[19] Massiot, D., Fayon, F., Capron, M., King, I., Le Calvé, S., Alonso, B., Durand, J.-O., Bujoli, B., Gan, Z. & Hoatson, G. Modelling one- and two-dimensional solid-state NMR spectra. *Magn. Reson. Chem.* **40**, 70–76 (2002).

[20] Åvall, G., Ferrero, G. A., Janßen, K. A., Exner, M., Son, Y. & Adelhelm, P. In situ pore formation in graphite through solvent co-intercalation: a new model for the formation of ternary graphite intercalation compounds bridging batteries and supercapacitors. *Adv. Energy Mater.* **13**, 2301944 (2023).

[21] Geisler, J., Pfeiffer, L., Ferrero, G. A., Axmann, P. & Adelhelm, P. Setup design and data evaluation for DEMS in sodium ion batteries, demonstrated on a Mn-rich cathode material. *Batteries & Supercaps* **7**, e202400006 (2024).
